# Site-Selective Electrochemical Intramolecular C(sp^2^)–H Selenylation for Dibenzoselenophene Synthesis

**DOI:** 10.1021/acs.joc.5c01600

**Published:** 2025-08-18

**Authors:** Indrajit Karmakar, Yi-Xuan Jian, Wan-Lin Cheng, Rekha Bai, Yu-Hsuan Chen, Chin-Fa Lee

**Affiliations:** † Department of Chemistry, 34916 National Chung Hsing University, Taichung 402, Taiwan, R.O.C; ‡ i-Center for Advanced Science and Technology (iCAST), National Chung Hsing University, Taichung 402, Taiwan, R.O.C; § Innovation and Development Center of Sustainable Agriculture (IDCSA), National Chung Hsing University, Taichung 402, Taiwan, R.O.C

## Abstract

We report a robust and eco-conscious electrosynthetic protocol for efficiently constructing structurally diverse and biologically relevant dibenzoselenophenes. This transformation proceeds *via* electrochemical intramolecular C–H activation of bis­(biaryl) diselenides, employing LiClO_4_ as a green electrolyte and tetrabutylammonium bromide as an additive, conducted in DMSO at 70 °C under a constant current of 10 mA using Pt/Pt electrodes. The newly developed method offers several notable advantages, including mild and energy-efficient conditions, transition-metal-free catalysis, short reaction times, high to excellent yields, broad substrate tolerance, scalability to gram-scale synthesis, operational simplicity, and environmental friendliness.

## Graphical abstract

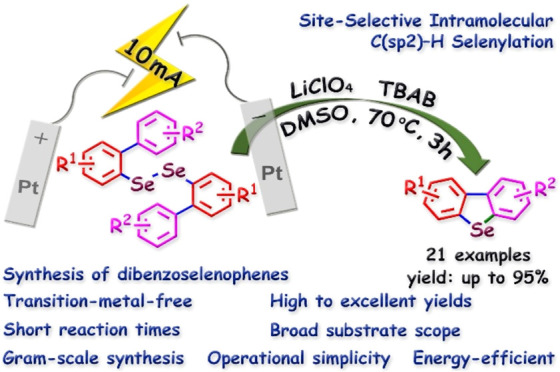

## 1 Introduction

Organoselenium compounds hold a significant place among both natural and synthetic bioactive molecules.^1^ Dibenzoselenophenes are recognized as valuable scaffolds in materials science, agricultural science, and pharmaceutical research due to their unique electronic properties and biological relevance.^2^

A few marketed drugs featuring selective selenium-containing heterocyclic structures are illustrated in Figure 1.^3^ McCullough *et al*. in 1950 utilized toxic liquid bromine to convert bis­(biaryl) diselenides into dibenzoselenophenes over a 48 h period.^4^ Yoshikai developed a versatile and flexible approach to benzoselenophene synthesis by integrating cobalt-catalyzed migratory arylzincation with subsequent cyclization reactions.^5^ Takimiya achieved the efficient synthesis of benzoselenopheno­[3,2-*b*]­benzoselenophene from bis­(biphenyl-4-yl)­acetylene by developing a straightforward and effective selenocyclization strategy.^6^ Jiang demonstrated an efficient approach for the synthesis of diaryl-annulated sulfides and selenides via S–I and Se–I exchange processes, accomplished without the use of transition metal catalysts.^7^ Hu developed a one-pot, three-step cascade hexadehydro-Diels–Alder reaction of tetraynes for the efficient synthesis of fused, multifunctionalized dibenzoselenophenes.^8^ Aganda and Lee have synthesized dibenzo­[*b*,*d*]­selenophenes through a two-step process involving silver catalysis under visible light irradiation.^9^ To address these limitations, including the use of toxic metal catalysts, hazardous chlorinated solvents, high temperatures, elimination of bulky leaving groups, long reaction times, and narrow substrate scope, we are investigating a more universal synthetic strategy for this class of biologically relevant molecules, aligning with our green chemistry research initiatives.^10^ C–H activation is a powerful strategy in modern organic synthesis that enables direct functionalization of typically inert carbon–hydrogen bonds. It offers atom economy and efficiency by eliminating the need for prefunctionalized substrates.^11^ The field of organic synthesis is increasingly embracing electrosynthetic approaches as promising alternatives to traditional methods.^12^ Only a limited number of electrochemical selenylation reactions have been reported in the literature, and it remains a significant challenge for organic chemists.^13^ Site-selective electrochemical intramolecular C­(sp^2^)-H functionalization enables precise modification of organic molecules by activating specific C–H bonds under mild, sustainable conditions. This approach offers an efficient route to complex structures without the need for prefunctionalization.

**Figure 1:**
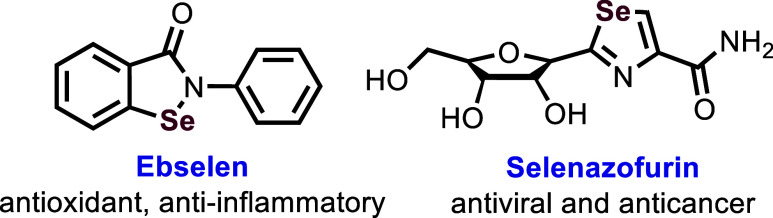
Selective selenium-containing heterocyclic drugs.

In line with our objectives, we have developed an efficient and practical electrosynthetic method for generating a wide range of diversely substituted dibenzo­[*b*,*d*]­selenophene derivatives. This is achieved via electrochemical intramolecular C–H activation of bis­(biaryl) diselenides in an undivided cell, employing constant direct current, lithium perchlorate (LiClO_4_) as the electrolyte, and tetrabutylammonium bromide (TBAB) as an additive in DMSO at 70 °C (Scheme 1). This newly developed electrochemical strategy surpasses earlier methods with respect to a broader substrate range, higher yields, shorter reaction times, and improved scalability.

**Scheme 1:**
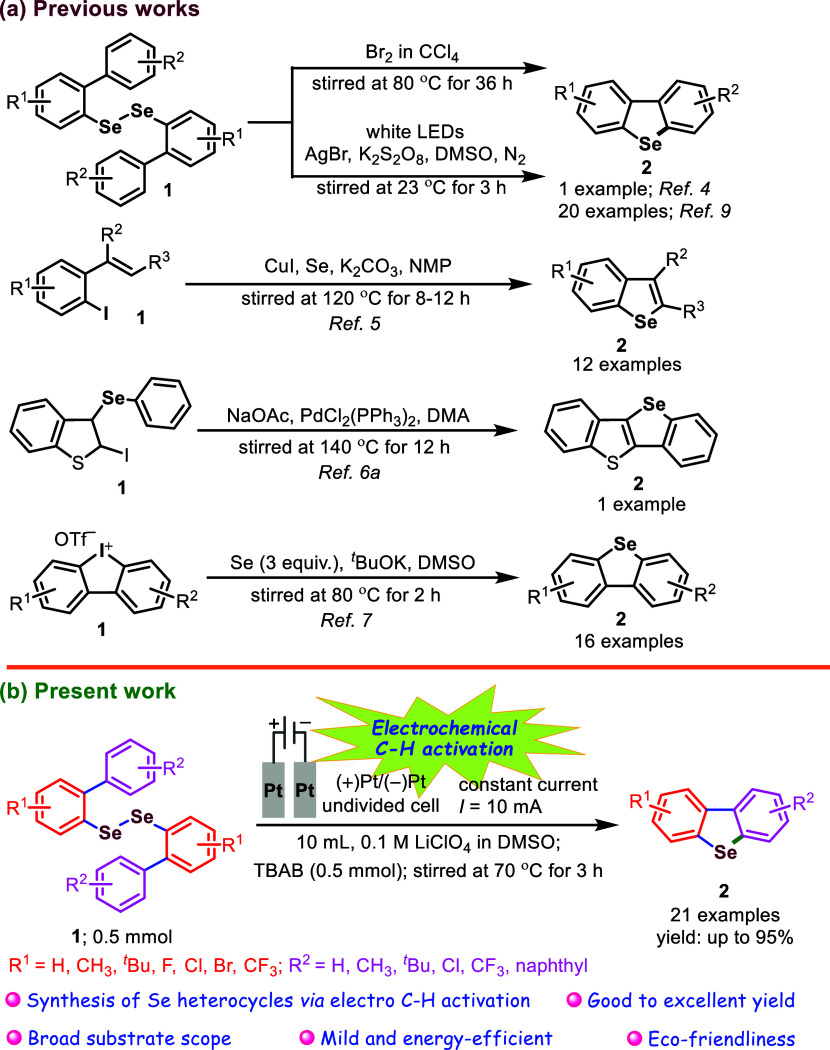
Electrochemical Synthesis of Diversely Substituted Dibenzoselenophenes (**2**)

## 2 Results and Discussion

To optimize the conditions for this electrosynthetic intramolecular C–H activation, we initiated our study with several test reactions employing the model compound 1,2-di­([1,1′-biphenyl]-2-yl)­diselane (**1aa**). As a preliminary test, we stirred a mixture of **1aa** and tetrabutylammonium bromide (TBAB) in DMSO at 70 °C, but no reaction was observed even after 3 h (Table 1, entry 1). A similar outcome was obtained when the reaction was carried out with 0.1 M lithium perchlorate (LiClO_4_) in DMSO under a constant current of 10 mA using Pt/Pt as undivided electrodes at 70 °C, but without TBAB (Table 1, entry 2). Proceeding further, we employed **1aa** (0.5 mmol) with TBAB (0.5 mmol) under electrochemical conditions in an undivided cell using Pt/Pt electrodes and a constant current of 10 mA, with 0.1 M LiClO_4_ in DMSO (10 mL) at 70 °C. Gratifyingly, this setup afforded dibenzo­[*b*,*d*]­selenophene (**2aa**) in 91% yield after 2 h (Table 1, entry 3). Maintaining all parameters except temperature, which was adjusted to 100 °C and room temperature, led to **2aa** yields of 88% and trace levels, respectively (Table 1, entries 4–5). Deviations from the optimal current flow led to diminished yields passing 20 mA current afforded 85% yield in 3 h (Table 1, entry 6), and 5 mA gave only 69% in 3 h (Table 1, entry 7); the reaction did not proceed at all without current for 3 h (Table 1, entry 8). To assess the effect of electrolyte concentration, we performed the model reaction using TBAB (0.5 mmol), Pt/Pt electrodes in an undivided cell at 10 mA DC, and isolated **2aa** in 90% yield at 3 h with 0.2 M LiClO_4_, and in 86% yield at 3 h with 0.05 M LiClO_4_, under 70 °C in DMSO (Table 1, entries 9 and 10). Additionally, we examined how changing the quantity of TBAB affected the model reaction, keeping all other parameters constant: Pt/Pt electrodes, 10 mA DC, 0.1 M LiClO_4_ in DMSO, and 70 °C. This resulted in **2aa** yields of 89 and 59% at 3 h (Table 1, entries 11 and 12). We also examined the effect of changing the electrode pair to combinations such as Pt/C, C/Pt, C/C, Pt/Cu, Pt/Ni, and Pt/Zn (Table 1, entries 13–18), which all proved to be ineffective. Additionally, five reactions were carried out using different solvents—CH_3_CN, CH_3_NO_2_, DMF, DCE, and water—while maintaining the rest of the reaction parameters unchanged; none of these reactions proceeded even after 3 h (Table 1, entries 19–23). To determine the optimal reaction conditions, we initially tested different supporting electrolytes (such as KI and NH_4_I) at a concentration of 0.1 M in DMSO, while keeping all other parameters constant (Table 1, entries 24–25). Subsequently, we explored the effect of various supporting additives (such as TBAI and TBAC) under the same conditions (Table 1, entries 26–27), which resulted in only trace amounts of product yield.

**Table 1:**
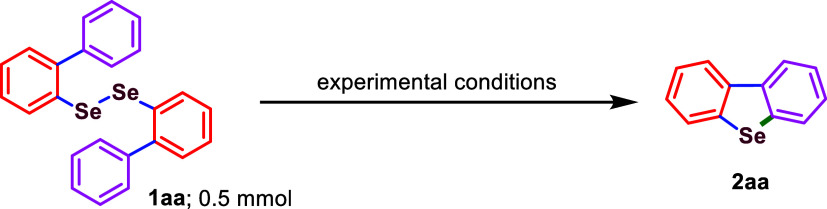
Optimization of Reaction Conditions

ent.	electrolyte (M)	additive (mmol)	cell (+/−)	solvent (10 mL)	temp. (°C)	curr. (mA)	time (h)	yield (%)^a^ ^,^ ^b^
1		TBAB (0.5)		DMSO	70		3	
2	LiClO_4_ (0.1)		Pt/Pt	DMSO	70	10	3	
**3**	**LiClO**_**4**_ **(0.1)**	**TBAB (0.5)**	**Pt/Pt**	**DMSO**	**70**	**10**	**3**	**91**
4	LiClO_4_ (0.1)	TBAB (0.5)	Pt**/**Pt	DMSO	100	10	3	88
5	LiClO_4_ (0.1)	TBAB (0.5)	Pt**/**Pt	DMSO	rt	10	3	trace
6	LiClO_4_ (0.1)	TBAB (0.5)	Pt**/**Pt	DMSO	70	20	3	85
7	LiClO_4_ (0.1)	TBAB (0.5)	Pt**/**Pt	DMSO	70	5	3	69
8	LiClO_4_ (0.1)	TBAB (0.5)	Pt/Pt	DMSO	70		3	
9	LiClO_4_ (0.2)	TBAB (0.5)	Pt/Pt	DMSO	70	10	3	90
10	LiClO_4_ (0.05)	TBAB (0.5)	Pt/Pt	DMSO	70	10	3	86
11	LiClO_4_ (0.1)	TBAB (1.0)	Pt/Pt	DMSO	70	10	3	89
12	LiClO_4_ (0.1)	TBAB (0.2)	Pt/Pt	DMSO	70	10	3	59
13	LiClO_4_ (0.1)	TBAB (0.5)	Pt**/**C	DMSO	70	10	3	56
14	LiClO_4_ (0.1)	TBAB (0.5)	C**/**Pt	DMSO	70	10	3	61
15	LiClO_4_ (0.1)	TBAB (0.5)	C**/**C	DMSO	70	10	3	63
16	LiClO_4_ (0.1)	TBAB (0.5)	Pt**/**Cu	DMSO	70	10	3	trace
17	LiClO_4_ (0.1)	TBAB (0.5)	Pt/Ni	DMSO	70	10	3	trace
18	LiClO_4_ (0.1)	TBAB (0.5)	Pt**/**Zn	DMSO	70	10	3	trace
19	LiClO_4_ (0.1)	TBAB (0.5)	Pt**/**Pt	CH_3_CN	70	10	3	trace
20	LiClO_4_ (0.1)	TBAB (0.5)	Pt**/**Pt	CH_3_NO_2_	70	10	3	
21	LiClO_4_ (0.1)	TBAB (0.5)	Pt**/**Pt	DMF	70	10	3	trace
22	LiClO_4_ (0.1)	TBAB (0.5)	Pt**/**Pt	DCM	70	10	3	
23	LiClO_4_ (0.1)	TBAB (0.5)	Pt/Pt	H_2_O	70	10	3	
24	KI (0.1)	TBAB (0.5)	Pt/Pt	DMSO	70	10	3	
25	NH_4_I (0.1)	TBAB (0.5)	Pt/Pt	DMSO	70	10	3	trace
26	LiClO_4_ (0.1)	TBAI (0.5)	Pt/Pt	DMSO	70	10	3	trace
27	LiClO_4_ (0.1)	TBAC (0.5)	Pt/Pt	DMSO	70	10	3	trace

a Reaction conditions: A mixture of 1,2-di­([1,1′-biphenyl]-2-yl)­diselane (1aa; 0.5 mmol) dissolved in various electrolyte solutions, in the presence or absence of additives, and subjected to electrolysis at a constant current in an undivided cell with a 0.5 cm electrode gap at varying temperatures. Electrode size: Pt, C, Ni, Cu, and Zn plates: 0.7 cm × 0.7 cm × 0.2 cm.

b Isolated yields. 1.22 F/mol for entry 3. M = molarity.

Finally, we established the standard reaction conditions for the electrochemical intramolecular C–H activation and functionalization of 1,2-di­([1,1′-biphenyl]-2-yl)­diselane (**1aa**, 0.5 mmol) as a model substrate, achieving optimal yield and reaction time. The reaction was conducted in an undivided cell using platinum plates as both the anode and cathode, with TBAB (0.5 mmol) as an additive and 0.1 M LiClO_4_ in DMSO (10 mL) as the electrolyte. Under a constant direct current of 10 mA at 70 °C, the desired product, dibenzo­[*b*,*d*]­selenophene (**2aa**), was obtained in 91% yield within 2 h (Table 1, entry 3). Table 1 summarizes all of the trial reactions conducted for the electrosynthetic process. Detailed spectral analysis (^1^H NMR and ^13^C NMR) was used to fully characterize compound **2aa**, whose physical and spectral properties were consistent with those previously reported in the literature.^9^ Further structural confirmation for compound **2bf** was done from its single X-ray analysis (Figure 2: *ORTEP* view; CCDC 2450651; unit cell parameters: *a* = 6.3622 (3) Å, *b* = 8.8554 (5) Å, *c* = 20.2197 (11) Å, α = 90°, β = 90°, γ = 90°; space group: *P*2_1_2_1_2_1_; see Supporting Information).

**Figure 2:**
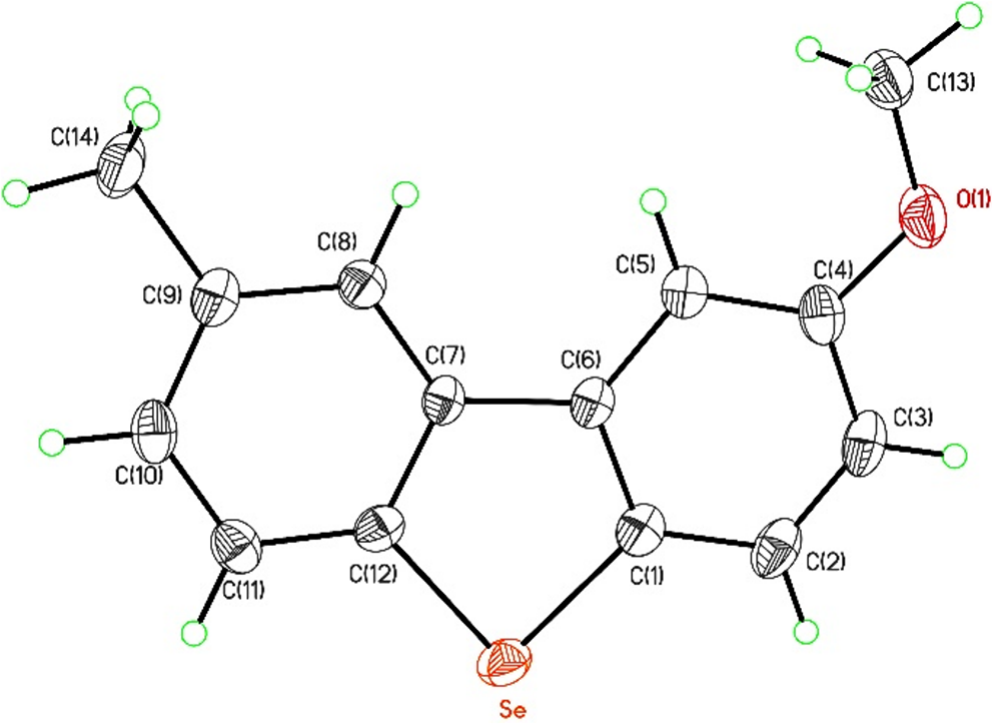
*ORTEP* view of compound **2bf**.

With the standard reaction conditions established, we proceeded to explore the substrate scope of this electrochemical protocol using six monosubstituted bis­(biaryl) diselenide analogues (**1ba**–**1ga**), bearing either electron-donating groups—such as methyl (**1ba**) and *tert*-butyl (**1ca**)—or electron-withdrawing groups—such as fluoro (**1da**), chloro (**1ea**), bromo (**1fa**), and trifluoromethyl (**1ga**)—at the 5-position of the aryl ring. Each substrate was subjected to the electrochemical reaction under the optimized conditions, and all underwent smooth transformation to afford the corresponding substituted dibenzo­[*b*,*d*]­selenophene derivatives (**2ba**-**2ga**) in excellent yields ranging from 80 to 95% within 3 h (Table 2, compounds **2ba**-**2ga**). Interestingly, when starting from a disubstituted bis­(biaryl) diselenide system such as 1,2-bis­(3′,5-dimethyl-[1,1′-biphenyl]-2-yl)­diselane (**1bb**), a 2:1 mixture of two regioisomeric products—2,8-dimethyldibenzo­[*b*,*d*]­selenophene (**2bb**) and 2,6-dimethyldibenzo­[*b*,*d*]­selenophene (**2bb′**)—is obtained in 72% yield within 3 h (Table 2, compounds **2bb** and **2bb′**). Encouraged by these results, we explored three different disubstituted bis­(biaryl) diselenide systems (**1bc**–**1be**) in which one phenyl ring bears a methyl group, while the other contains a *tert*-butyl (**1bc**), chloro (**1bd**), or trifluoromethyl (**1be**) substituent. These combinations allowed us to probe the influence of both steric and electronic effects on the reaction outcome. Subjecting these to identical reaction conditions afforded the corresponding dibenzoselenophene derivatives **2bc–2be** in good to excellent yields from 61–89%, within 3 h (Table 2, compounds **3ba–3cf**). Building on these findings, the reaction of a 3,5-disubstituted bis­(biaryl) diselenide—specifically, 1,2-bis­(3′-methoxy-5-methyl-[1,1′-biphenyl]-2-yl)­diselane (**1bf**)—under identical conditions furnished a 3:1 mixture of two regioisomeric products: 2-methoxy-8-methyldibenzo­[*b*,*d*]­selenophene (**2bf**) and 6-methoxy-2-methyldibenzo­[*b*,*d*]­selenophene (**2bf′**). The transformation proceeded smoothly, affording the isomeric mixture in 70% overall yield within 3 h (Table 2, compounds **2bf** and **2bf′**). To further broaden the scope of this transformation, we investigated seven additional disubstituted bis­(biaryl) diselenide substrates (**1bg**–**1gb**), incorporating a diverse array of functional groups. These included both electron-donating (*e.g*., methyl) and electron-withdrawing substituents (*e.g*., fluoro, chloro, bromo, trifluoromethyl), as well as extended aromatic systems such as naphthalene (**1bg**, **1dg**). Several compounds featured mixed substitution patterns (*e.g*., fluoro and methyl in **1db**; fluoro and chloro in **1dd**; chloro and methyl in **1eb**; bromo and methyl in **1fb** and trifluoromethyl and methyl in **1gb**), allowing for the evaluation of combined electronic and steric influences under the established electrochemical protocol. Remarkably, each substrate underwent smooth conversion, delivering the desired dibenzoselenophene derivatives **2bg–2gb** in good to excellent yields ranging from 60 to 95% within 3 h (Table 2, compounds **2bg–2gb**).

**Table 2:**
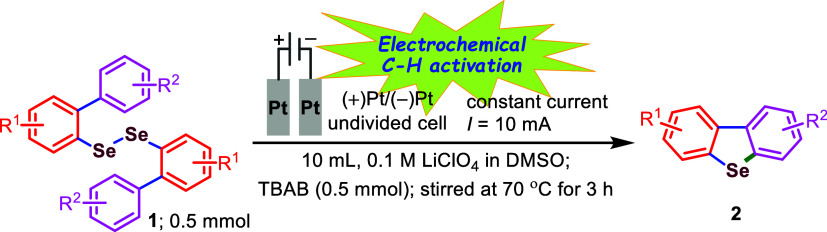

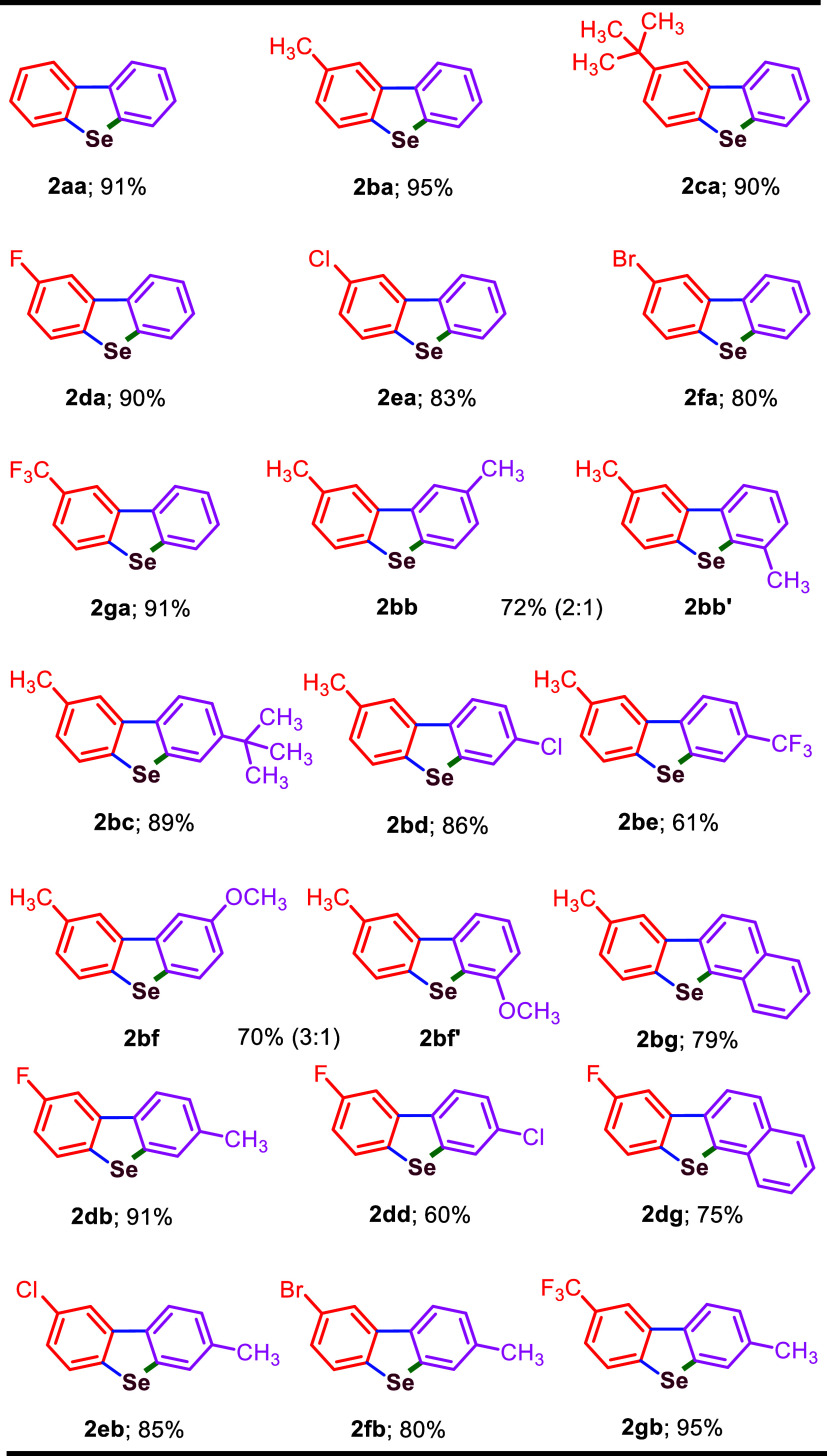
Electrochemical Synthesis of Diversely Functionalized Dibenzoselenophenes (**2**)­^a^ ^,^ ^b^

a Reaction conditions: A mixture of 1,2-di­([1,1′-biaryl]-2-yl)­diselane (**1**; 0.5 mmol) and TBAB (0.5 mmol) dissolved in 0.1 M LiClO4 in DMSO (10 mL) at a constant current (*I* = 10 mA) in an undivided Pt/Pt cell (effective size: 0.7 cm × 0.7 cm × 0.2 cm) with a 0.5 cm electrode gap at 70 °C temperatures for 3 h.

b Isolated yields.

We further applied this protocol to larger-scale synthetic reactions (2.5 mmol scale; a 5-fold increase, see Scheme 2) for two representative compounds, **1aa** and **1gb**. The method performed well in both cases, affording yields of 73 and 77% for compounds **2aa** and **2gb**, respectively (see the Experimental Section). Although the yields on the larger scale were slightly lower compared to those obtained on the smaller (millimolar) scale, the reactions proceeded effectively, albeit requiring slightly longer reaction times.

**Scheme 2:**
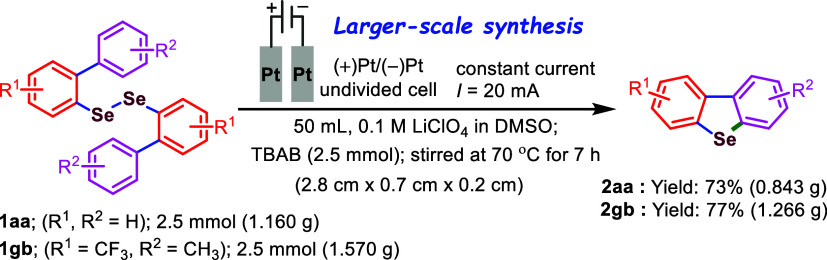
Larger-Scale Synthetic Applications

At this stage, we intend to investigate the possible mechanism underlying the intramolecular electrochemical ortho-selective selenylation that affords dibenzoselenophenes (**2**). To gain insight into the mechanistic pathway, we first conducted a series of control experiments using our model reaction (Scheme 3). When the reaction was carried out under standard conditions in the presence of various radical-trapping agents—such as TEMPO, BHT, and *p*-benzoquinone—the formation of the target product **2aa** was completely suppressed (Scheme 3a), suggesting that the transformation proceeds via a radical pathway. Intermediate **3** was also detected via high-resolution mass spectrometry (HRMS) analysis (Scheme 3b), supporting our hypothesis on the formation of potential radical intermediates **1′a** and the bromine radical.

**Scheme 3:**
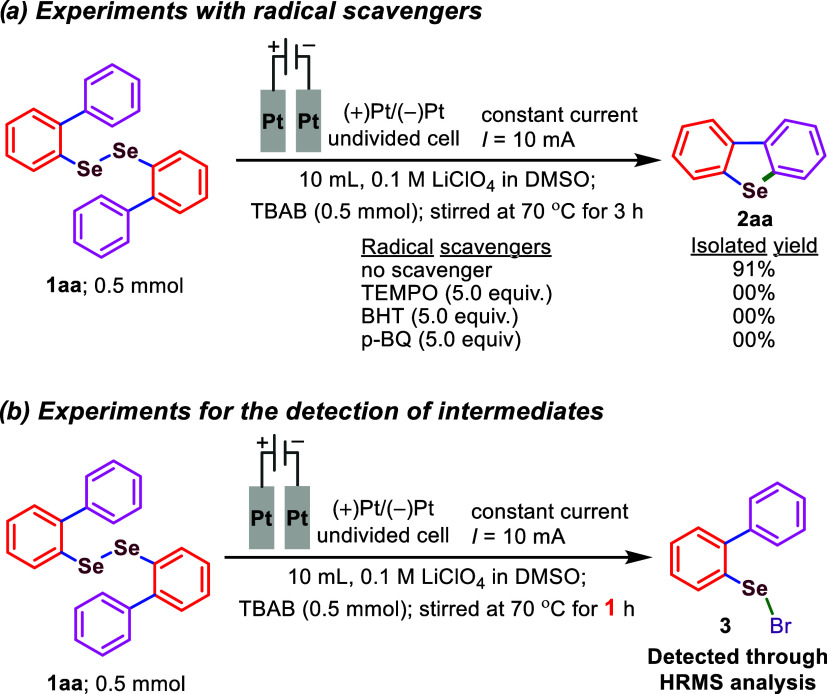
Control Experiments

Based on detailed control experiments and cyclic voltammetry studies of the reaction components, we propose a tentative mechanism for the intramolecular electrochemically promoted transformation, as illustrated in Scheme 4.^14^ Cyclic voltammetry studies (Figures 3 and 4) of the model substrate **1aa** (*i.e*., 1,2-di­([1,1′-biphenyl]-2-yl)­diselane) revealed an oxidation potential of +1.42 V (vs Ag/Ag^+^). This indicates that **1aa** can be readily oxidized at the anode surface, losing one electron to form radical cation intermediate **1′**. The Se–Se bond in **1′** then cleaves to generate a selenium-centered radical (**1′a**) and a selenium cation (**1′b**), respectively. Cyclic voltammetry of TBAB shows two distinct oxidation peaks at +0.95 and +1.31 V (vs Ag/Ag^+^), suggesting that bromide ions (Br^–^) undergo anodic oxidation to form bromine radicals. Radical heterocoupling between intermediates **1′a** and the bromine radical leads to the formation of intermediate **3** (also detected by HRMS). Notably, the oxidation peak shifts from +1.31 to +1.61 V (vs Ag/Ag^+^), which indirectly supports the generation of intermediate **3**. Subsequently, intermediate **3** undergoes intramolecular nucleophilic attack by the ortho-carbon of the aryl ring, leading to bromine substitution and the formation of intermediate **4**. In the final step, intermediate **4** undergoes facile aromatization to yield the desired product **2** through the loss of one proton. At the cathode, protons are reduced to produce hydrogen gas, and simultaneously, **1′b** undergoes a one-electron reduction to regenerate **1aa**, as evidenced by the cyclic voltammogram of 1aa, which shows a reduction potential at +1.35 V vs Ag/Ag^+^ (Figure 4 and Scheme 4).

**Figure 3:**
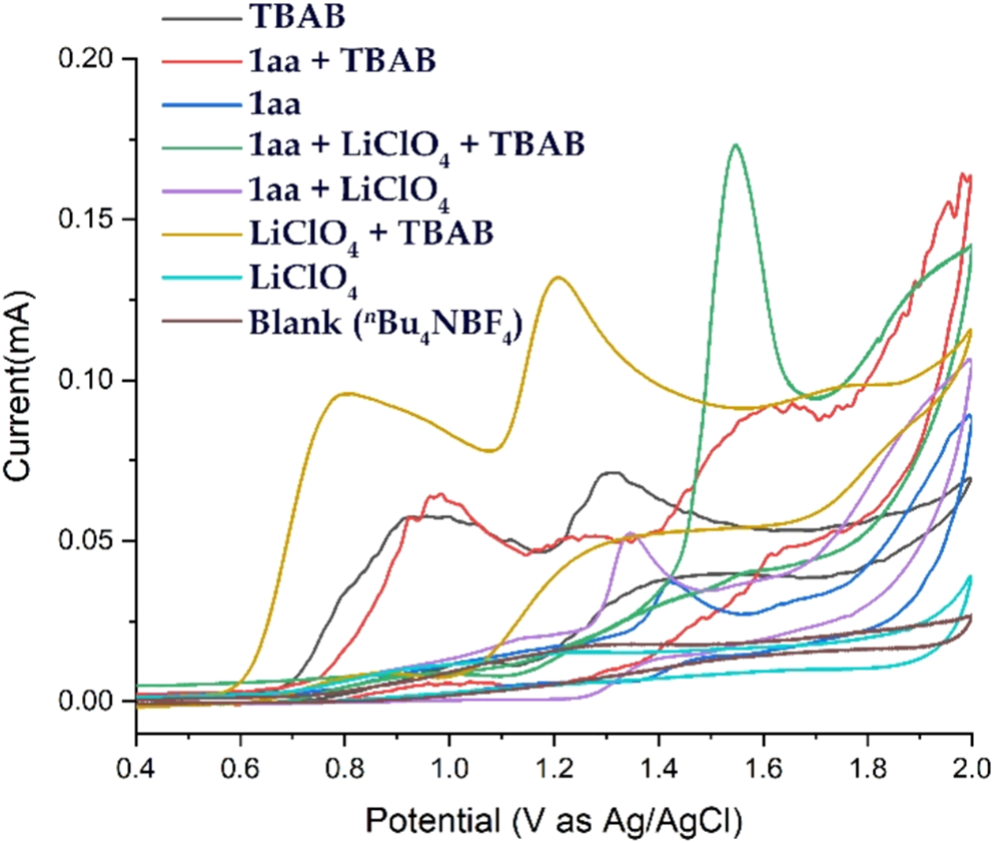
Cyclic voltammetry diagram. CV plotting convension: IUPAC; working electrode: BAS glassy carbon; counter electrode: Platinum wire; reference electrodes: Ag/Ag^+^ electrode; solvent: CH_3_CN (10 mL); Electrolyte: ^***n***^ **Bu**_**4**_ **NBF**_**4**_ (0.09 M); analyte: **1aa** (0.0002 M); **LiClO**_**4**_ (0.0009 M); **TBAB** (0.0003 M); temperature: Room temperature; starting point: 0.0; direction of scan: +Direction; the potential scan ranged from −2.0 to +2.0 V at a scan rate of 0.1 V/s for each case.

**Figure 4:**
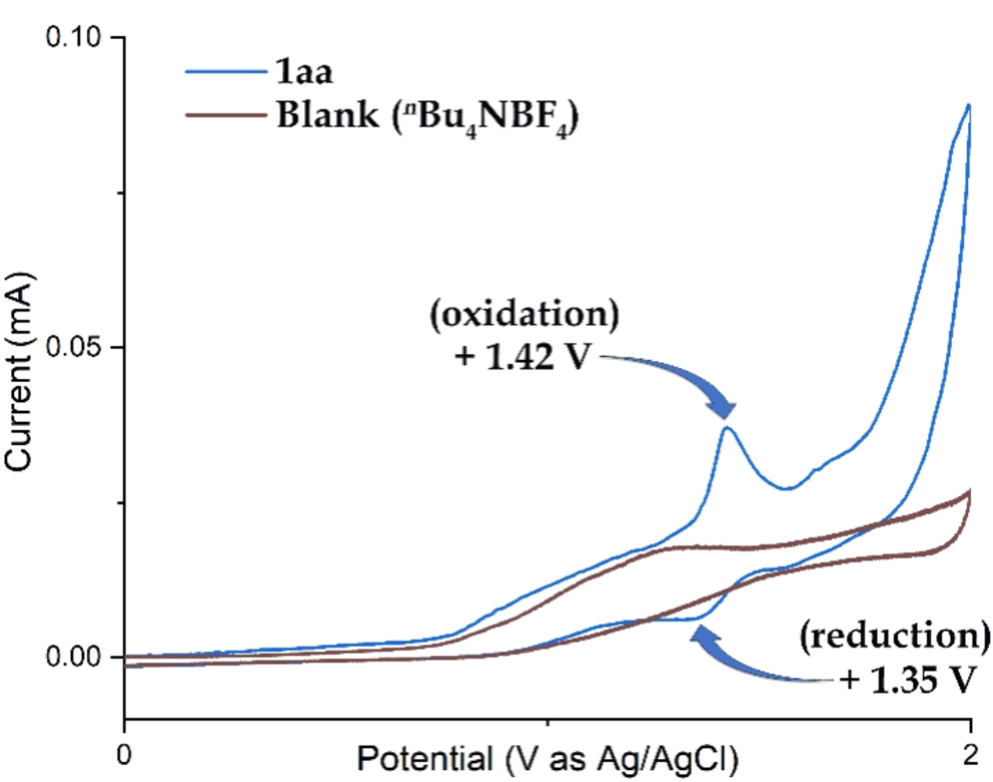
Cyclic voltammetry diagram of **1aa**; solvent: CH_3_CN (10 mL); electrolyte: ^***n***^ **Bu**_**4**_ **NBF**_**4**_ (0.09 M); analyte: **1aa** (0.0002 M).

**Scheme 4:**
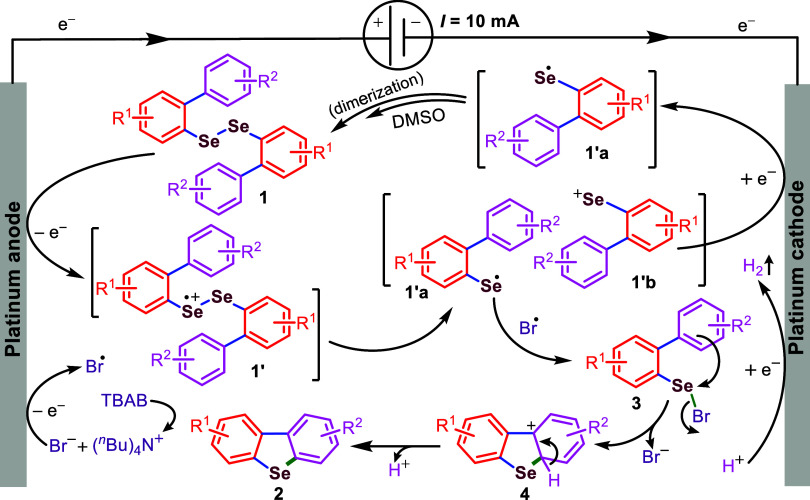
Proposed Mechanism for the Electrochemical Intramolecular C­(sp^2^)–H Selenylation

## 3 Conclusions

In conclusion, we have developed a robust and environmentally friendly electrosynthetic protocol for the efficient intramolecular construction of structurally diverse and biologically relevant dibenzoselenophenes from bis­(biaryl) diselenides *via* site-selective C­(sp^2^)–H selenylation. This transformation proceeds through electrochemical intramolecular C–H selenylation, using LiClO_4_ as a green electrolyte and tetrabutylammonium bromide as an additive, in DMSO at 70 °C under a constant current of 10 mA with Pt/Pt electrodes. The newly established method offers several significant advantages, including mild and energy-efficient conditions, transition-metal-free catalysis, short reaction times, high to excellent yields, broad substrate scope, gram-scale scalability, operational simplicity, and environmental sustainability. We are currently conducting further investigations into such electrochemical functionalization processes in our laboratory.

## 4 Experimental Section

### 4.1 General Method

All solvents used in this study were distilled and dried before use according to standard procedures. ^1^H, ^13^C, and ^19^F NMR spectra were collected at Varian Unity Inova-600 or a Varian Mercury-400 NMR instrument using CDCl_3_ as a solvent. Chemical shifts were reported in δ (ppm), relative to the internal standard, TMS. The signals observed are described as s (singlet), d (doublet), t (triplet), and m (multiplet). Coupling constants are reported as *J* values in Hz. Mass spectrometry was obtained using a Jeol JMS-HX 110 spectrometer. The X-ray diffraction measurements were carried out using a Bruker D8 VENTURE XRD instrument. Cyclic voltammetry was performed on a CHI Instruments 750A potentiostat using acetonitrile as the solvent. The melting points were recorded on a Büchi 535 melting point apparatus and are uncorrected. Thin-Layer Chromatography (TLC) was performed using silica gel 60 F_254_ (Merck) plates. An undivided electrochemical cell equipped with platinum electrodes (IKA) was used, with dimensions of 0.7 cm × 0.7 cm × 0.2 cm for small-scale reactions and 2.8 cm × 0.7 cm × 0.2 cm for larger-scale reactions. A GW Instek GPS-2303 Laboratory DC Power Supply (350 W, 450 VA, 50/60 Hz) was employed as the power source.

### 4.2 General Procedure for the Synthesis of Dibenzoselenophenes in the Presence of an Electrochemical Cell

An oven-dried glass vessel was sequentially charged with bis­(biaryl) diselenides (**1**; 0.5 mmol), TBAB (0.5 mmol), 10 mL of a 0.1 M LiClO_4_ electrolyte solution in DMSO, and a magnetic stir bar. The vessel was then sealed with a pair of platinum plate electrodes (each measuring 0.7 cm × 0.7 cm × 0.2 cm) positioned 0.5 cm apart, forming an undivided electrochemical cell. A constant direct current of 10 mA was applied across the reaction mixture while maintaining a temperature of 70 °C. The mixture was stirred continuously at ambient pressure for 3 h. The progress of the reaction was monitored by TLC. Upon completion of the reaction, 20 mL of a 3:1 (v/v) mixture of ethyl acetate and water was added to the reaction mixture and transferred to a separating funnel. The mixture was thoroughly shaken, and the organic layer was separated and dried over anhydrous sodium sulfate. The solvent was then removed under reduced pressure, and the resulting crude product was purified by column chromatography using ethyl acetate/hexane mixtures as eluents. This process afforded the desired product dibenzoselenophenes **2** (**2aa**–**2gb**). All of the synthesized compounds **2** (**2aa**–**2gb**; a total of 21 compounds) were purified by flash column chromatography (see the Experimental Section). Except for **2aa**, **2ba**, **2da**, and **2bd**, all other synthesized compounds are new. Each synthesized compound was fully characterized based on its detailed spectral studies, including ^1^H NMR, ^13^C NMR, ^19^F NMR (for **2da**, **2ga**, **2be**, **2db**, **2dd**, **2dg**, and **2gb**), and HRMS (see the Experimental Section). Further, single-crystal X-ray analysis (CCDC 2450651) (see the Supporting Information) for the entry, 2-methoxy-8-methyldibenzo­[*b*,*d*]­selenophene (**2bf**; Table 2) fully supports the chemical structure as deduced based on its spectral analyses.

### 4.3 Large-Scale Synthesis of Compounds **2aa** and **2gb** under an Electrochemical Cell

An oven-dried glass vessel was sequentially charged with 1,2-di­([1,1′-biphenyl]-2-yl)­diselane/1,2-bis­(4′-methyl-5-(trifluoromethyl)-[1,1′-biphenyl]-2-yl)­diselane (**1aa/1gb**; 2.5 mmol, 1.160 g/1.570 g), TBAB (2.5 mmol, 0.805 g), 50 mL of a 0.1 M LiClO_4_ (0.530 g) electrolyte solution in DMSO, and a magnetic stir bar. The vessel was then sealed with a pair of platinum plate electrodes (each measuring 2.8 cm × 0.7 cm × 0.2 cm) positioned 0.5 cm apart, forming an undivided electrochemical cell. A constant direct current of 20 mA was applied across the reaction mixture while maintaining a temperature of 70 °C. The mixture was stirred continuously at ambient pressure for 7 h. The progress of the reaction was monitored by TLC. Upon completion of the reaction, 80 mL of a 3:1 (v/v) mixture of ethyl acetate and water was added to the reaction mixture and transferred to a separating funnel. The mixture was thoroughly shaken, and the organic layer was separated and dried over anhydrous sodium sulfate. The solvent was then removed under reduced pressure, and the resulting crude product was purified by column chromatography using ethyl acetate–hexane mixtures as eluents. This process afforded the desired product dibenzo­[*b*,*d*]­selenophene/7-methyl-2-(trifluoromethyl)­dibenzo­[*b*,*d*]­selenophene (**2aa/2gb**) with 73/77% yield (0.843 g/1.266 g).

### 4.4 Physical and Spectral Data of All of the Synthesized Compounds **2** (**2aa**–**2gb**)

#### Dibenzo­[*b*,*d*]­selenophene (**2aa**)^9^

It was synthesized following the general procedure using 1,2-di­([1,1′-biphenyl]-2-yl)­diselane (**1aa**; 0.5 mmol, 0.232 g), TBAB (0.5 mmol, 0.161 g), LiClO_4_ (0.1 M, 0.106 g), and 10 mL of DMSO, and the crude product was then purified by column chromatography (SiO_2_, 10–15% ethyl acetate in hexanes) to provide **2aa** as a white solid; yield: 91% (210 mg; 0.5 mmol scale); mp = 60–61 °C. ^1^H NMR (CDCl_3_, 400 MHz): δ = 8.15 (d, *J* = 7.7 Hz, 2H), 7.90 (d, *J* = 7.9 Hz, 2H), 7.50–7.46 (m, 2H), 7.42–7.38 (m, 2H) ppm. ^13^C­{^1^H} NMR (CDCl_3_, 100 MHz): δ = 139.4, 138.4, 126.9 (2C), 126.2 (2C), 124.9 (2C), 122.9 (2C) ppm.

#### 2-Methyldibenzo­[*b*,*d*]­selenophene (**2ba**)^9^

It was synthesized following the general procedure using 1,2–1,2-bis­(5-methyl-[1,1′-biphenyl]-2-yl)­diselane (**1ba**; 0.5 mmol, 0.246 g), TBAB (0.5 mmol, 0.161 g), LiClO_4_ (0.1 M, 0.106 g), and 10 mL of DMSO, and the crude product was then purified by column chromatography (SiO_2_, 10–15% ethyl acetate in hexanes) to provide **2ba** as a white solid; yield: 95% (233 mg; 0.5 mmol scale); mp = 74–75 °C. ^1^H NMR (CDCl_3_, 400 MHz): δ = 8.11 (d, *J* = 7.8 Hz, 1H), 7.95 (s, 1H), 7.88 (d, *J* = 8.3 Hz, 1H), 7.77 (d, *J* = 8.1 Hz, 1H), 7.47–7.43 (m, 1H), 7.40–7.36 (m, 1H), 7.23 (d, *J* = 8.1 Hz, 1H), 2.52 (s, 3H) ppm. ^13^C­{^1^H} NMR (CDCl_3_, 100 MHz): δ = 139.7, 138.5, 138.3, 136.0, 134.7, 128.4, 126.8, 126.2, 125.8, 124.9, 123.3, 122.9, 21.6 ppm.

#### 2-(*tert*-Butyl)­dibenzo­[*b*,*d*]­selenophene (**2ca**)

It was synthesized following the general procedure using 1,2-bis­(5-(*tert*-butyl)-[1,1′-biphenyl]-2-yl)­diselane (**1ca**; 0.5 mmol, 0.289 g), TBAB (0.5 mmol, 0.161 g), LiClO_4_ (0.1 M, 0.106 g), and 10 mL of DMSO, and the crude product was then purified by column chromatography (SiO_2_, 10–15% ethyl acetate in hexanes) to provide **2ca** as a yellow oil; yield: 90% (258 mg; 0.5 mmol scale). ^1^H NMR (CDCl_3_, 400 MHz): δ = 8.23–8.22 (m, 2H), 7.93 (d, *J* = 7.9 Hz, 1H), 7.86 (d, *J* = 8.4 Hz, 1H), 7.54–7.49 (m, 2H), 7.44–7.40 (m, 1H), 1.52 (s, 9H) ppm. ^13^C­{^1^H} NMR (CDCl_3_, 100 MHz): δ = 148.2, 139.8, 138.6, 138.2, 136.2, 126.7, 126.3, 125.7, 125.0, 124.8, 122.8, 119.4, 34.9, 31.7 (3C) ppm. HRMS (EI) calcd for C_16_H_16_Se [M]^+^ 288.0417, found 288.0412.

#### 2-Fluorodibenzo­[*b*,*d*]­selenophene (**2da**)^9^

It was synthesized following the general procedure using 1,2-bis­(5-fluoro-[1,1′-biphenyl]-2-yl)­diselane (**1da**; 0.5 mmol, 0.250 g), TBAB (0.5 mmol, 0.161 g), LiClO_4_ (0.1 M, 0.106 g), and 10 mL of DMSO, and the crude product was then purified by column chromatography (SiO_2_, 10–15% ethyl acetate in hexanes) to provide **2da** as a white solid; yield: 90% (224 mg; 0.5 mmol scale); mp = 83 °C. ^1^H NMR (CDCl_3_, 400 MHz): δ = 8.04 (d, *J* = 7.8 Hz, 1H), 7.88 (d, *J* = 7.7 Hz, 1H), 7.81–7.77 (m, 2H), 7.49–7.39 (m, 2H), 7.18–7.13 (m, 1H) ppm. ^13^C­{^1^H} NMR (CDCl_3_, 100 MHz): δ = 161.6 (d, *J*_CF_ = 242 Hz), 140.6, 139.9 (d, *J*_CF_ = 8 Hz), 137.7, 133.8, 127.4, 127.1 (d, *J*_CF_ = 8 Hz), 126.3, 125.0, 123.2, 115.0 (d, *J*_CF_ = 24 Hz), 109.4 (d, *J*_CF_ = 23 Hz) ppm. ^19^F NMR (CDCl_3_, 376 MHz): δ = −117.86 ppm.

#### 2-Chlorodibenzo­[*b*,*d*]­selenophene (**2ea**)^7^

It was synthesized following the general procedure using 1,2-bis­(5-chloro-[1,1′-biphenyl]-2-yl)­diselane (**1ea**; 0.5 mmol, 0.267 g), TBAB (0.5 mmol, 0.161 g), LiClO_4_ (0.1 M, 0.106 g), and 10 mL of DMSO, and the crude product was then purified by column chromatography (SiO_2_, 10–15% ethyl acetate in hexanes) to provide **2ea** as a white solid; yield: 83% (221 mg; 0.5 mmol scale); mp = 72–73 °C. ^1^H NMR (CDCl_3_, 400 MHz): δ = 8.06 (d, *J* = 2.1 Hz, 1H), 8.04 (dd, *J* = 7.9 and 1.4 Hz, 1H), 7.88–7.85 (m, 1H), 7.76 (d, *J* = 8.4 Hz, 1H), 7.48–7.39 (m, 2H), 7.34 (dd, *J* = 8.4 and 2.1 Hz, 1H) ppm. ^13^C­{^1^H} NMR (CDCl_3_, 100 MHz): δ = 140.2, 139.8, 137.2 (2C), 131.3, 127.5, 127.0, 126.9, 126.2, 125.1, 123.1, 122.8 ppm.

#### 2-Bromodibenzo­[*b*,*d*]­selenophene (**2fa**)

It was synthesized following the general procedure using 1,2-bis­(5-bromo-[1,1′-biphenyl]-2-yl)­diselane (**1fa**; 0.5 mmol, 0.311 g), TBAB (0.5 mmol, 0.161 g), LiClO_4_ (0.1 M, 0.106 g), and 10 mL of DMSO, and the crude product was then purified by column chromatography (SiO_2_, 10–15% ethyl acetate in hexanes) to provide **2fa** as a white solid; yield: 80% (248 mg; 0.5 mmol scale); mp = 91–92 °C. ^1^H NMR (CDCl_3_, 400 MHz): δ = 8.22 (d, *J* = 2.0 Hz, 1H), 8.04 (d, *J* = 7.8 Hz, 1H), 7.88–7.85 (m, 1H), 7.70 (d, *J* = 8.4 Hz, 1H), 7.48–7.39 (m, 3H) ppm. ^13^C­{^1^H} NMR (CDCl_3_, 100 MHz): δ = 140.2, 140.0, 137.9, 137.2, 129.7, 127.6, 127.4, 126.2, 125.9, 125.2, 123.1, 119.1 ppm. HRMS (EI) calcd for C_12_H_7_BrSe [M]^+^ 309.8896, found 309.8905.

#### 2-(Trifluoromethyl)­dibenzo­[*b*,*d*]­selenophene (**2ga**)

It was synthesized following the general procedure using 1,2-bis­(5-(trifluoromethyl)-[1,1′-biphenyl]-2-yl)­diselane (**1ga**; 0.5 mmol, 0.300 g), TBAB (0.5 mmol, 0.161 g), LiClO_4_ (0.1 M, 0.106 g), and 10 mL of DMSO, and the crude product was then purified by column chromatography (SiO_2_, 10–15% ethyl acetate in hexanes) to provide **2ga** as a white solid; yield: 91% (272 mg; 0.5 mmol scale); mp = 81–82 °C. ^1^H NMR (CDCl_3_, 400 MHz): δ = 8.34 (s, 1H), 8.13 (d, *J* = 7.6 Hz, 1H), 7.95 (d, *J* = 8.3 Hz, 1H), 7.88 (d, *J* = 7.7 Hz, 1H), 7.61 (d, *J* = 8.3 Hz, 1H), 7.51–7.42 (m, 2H) ppm. ^13^C­{^1^H} NMR (CDCl_3_, 100 MHz): δ = 143.4, 139.9, 138.5, 137.4, 127.8, 127.6 (d, *J*_CF_ = 32 Hz), 126.5, 126.2, 125.3, 124.7 (d, *J*_CF_ = 270 Hz, *C*F_3_), 123.2, 123.1–122.9 (m), 119.8–119.6 (m) ppm. ^19^F NMR (CDCl_3_, 376 MHz): δ = −61.46 ppm. HRMS (EI) calcd for C_13_H_7_F_3_Se [M]^+^ 299.9665, found 299.9655.

#### 2,8-Dimethyldibenzo­[*b*,*d*]­selenophene (**2bb**) and 2,8–2,6-Dimethyldibenzo­[*b*,*d*]­selenophene (**2bb′**) (**1:1**)

It was synthesized following the general procedure using 1,2-bis­(3′,5-dimethyl-[1,1′-biphenyl]-2-yl)­diselane (**1bb**; 0.5 mmol, 0.260 g), TBAB (0.5 mmol, 0.161 g), LiClO_4_ (0.1 M, 0.106 g), and 10 mL of DMSO, and the crude product was then purified by column chromatography (SiO_2_, 10–15% ethyl acetate in hexanes) to provide **2bb** and **2bb′** (1:1) as a white solid; yield: 72% (186 mg; 0.5 mmol scale); mp = 73–74 °C. ^1^H NMR (CDCl_3_, 400 MHz): δ = 7.97–7.93 (m, 3H), 7.79 (d, *J* = 8.1 Hz, 1H), 7.75 (d, *J* = 8.0 Hz, 1H), 7.40 (t, *J* = 7.6 Hz, 1H), 7.24–7.20 (m, 3H), 2.56 (s, 3H), 2.52 (s, 3H), 2.52 (s, 3H) ppm. ^13^C­{^1^H} NMR (CDCl_3_, 100 MHz): δ = 140.9, 139.3, 138.5, 138.2, 136.3, 135.8, 135.0, 134.7, 134.6, 128.3, 126.9, 125.9, 125.8, 125.4, 123.6, 123.3, 120.3, 22.6, 21.6, 21.6 ppm. HRMS (EI) calcd for C_14_H_12_Se [M]^+^ 260.0104, found 260.0114.

#### 7-(*tert*-Butyl)-2-methyldibenzo­[*b*,*d*]­selenophene (**2bc**)

It was synthesized following the general procedure using 1,2-bis­(4′-(*tert*-butyl)-5-methyl-[1,1′-biphenyl]-2-yl)­diselane (**1bc**; 0.5 mmol, 0.303 g), TBAB (0.5 mmol, 0.161 g), LiClO_4_ (0.1 M, 0.106 g), and 10 mL of DMSO, and the crude product was then purified by column chromatography (SiO_2_, 10–15% ethyl acetate in hexanes) to provide **2bc** as a yellow oil; yield: 89% (268 mg; 0.5 mmol scale). ^1^H NMR (CDCl_3_, 400 MHz): δ = 8.03 (d, *J* = 8.3 Hz, 1H), 7.91–7.90 (m, 2H), 7.75 (d, *J* = 8.1 Hz, 1H), 7.51 (dd, *J* = 8.4 and 1.8 Hz, 1H), 7.20 (d, *J* = 7.9 Hz, 1H), 2.52 (s, 3H), 1.43 (s, 9H) ppm. ^13^C­{^1^H} NMR (CDCl_3_, 100 MHz): δ = 150.3, 139.8, 138.6, 135.9, 134.6, 127.9, 125.8, 123.1, 122.8, 122.6, 122.4, 35.2, 31.6, 21.6 ppm. HRMS (EI) calcd for C_17_H_18_Se [M]^+^ 302.0574, found 302.0568.

#### 7-Chloro-2-methyldibenzo­[*b*,*d*]­selenophene (**2bd**)

It was synthesized following the general procedure using 1,2-bis­(4′-chloro-5-methyl-[1,1′-biphenyl]-2-yl)­diselane (**1bd**; 0.5 mmol, 0.281 g), TBAB (0.5 mmol, 0.161 g), LiClO_4_ (0.1 M, 0.106 g), and 10 mL of DMSO, and the crude product was then purified by column chromatography (SiO_2_, 10–15% ethyl acetate in hexanes) to provide **2bd** as a white solid; yield: 86% (280 mg; 0.5 mmol scale); mp = 65–67 °C. ^1^H NMR (CDCl_3_, 400 MHz): δ = 7.96 (d, *J* = 8.5 Hz, 1H), 7.87 (s, 1H), 7.83 (d, *J* = 2.0 Hz, 1H), 7.73 (d, *J* = 8.1 Hz, 1H), 7.40 (dd, *J* = 8.5 and 2.0 Hz, 1H), 7.23 (d, *J* = 8.3 Hz, 1H), 2.51 (s, 3H) ppm. ^13^C­{^1^H} NMR (CDCl_3_, 100 MHz): δ = 140.8, 137.6, 136.8, 136.0, 135.0, 132.6, 128.7, 125.8, 125.8, 125.5, 123.5, 123.3, 21.6 ppm. HRMS (EI) calcd for C_13_H_9_ClSe [M]^+^ 279.9558, found 279.9550.

#### 2-Methyl-7-(trifluoromethyl)­dibenzo­[*b*,*d*]­selenophene (**2be**)

It was synthesized following the general procedure using 1,2-bis­(5-methyl-4′-(trifluoromethyl)-[1,1′-biphenyl]-2-yl)­diselane (**1be**; 0.5 mmol, 0.314 g), TBAB (0.5 mmol, 0.161 g), LiClO_4_ (0.1 M, 0.106 g), and 10 mL of DMSO, and the crude product was then purified by column chromatography (SiO_2_, 10–15% ethyl acetate in hexanes) to provide **2be** as a white solid; yield: 61% (191 mg; 0.5 mmol scale); mp = 71–73 °C. ^1^H NMR (CDCl_3_, 400 MHz): δ = 8.16–8.14 (m, 2H), 7.97 (s, 1H), 7.78 (d, *J* = 8.1 Hz, 1H), 7.67 (d, *J* = 8.3 Hz, 1H), 7.29 (d, *J* = 8.1 Hz, 1H), 2.53 (s, 3H) ppm. ^13^C­{^1^H} NMR (CDCl_3_, 100 MHz): δ = 141.2, 139.8, 137.3, 137.2, 135.2, 129.6, 128.3 (d, *J*_CF_ = 33 Hz), 125.9, 124.4 (d, *J*_CF_ = 271 Hz, *C*F_3_), 123.9, 123.5–123.3 (m), 122.9, 121.8–121.7 (m), 21.5 ppm. ^19^F NMR (CDCl_3_, 376 MHz): δ = – 61.56 ppm. HRMS (EI) calcd for C_14_H_9_F_3_Se [M]^+^ 313.9822, found 313.9812.

#### 2-Methoxy-8-methyldibenzo­[*b*,*d*]­selenophene (**2bf**) and 6-Methoxy-2-methyldibenzo­[*b*,*d*]­selenophene (**2bf′**) (**3:1**)

It was synthesized following the general procedure using 1,2-bis­(3′-methoxy-5-methyl-[1,1′-biphenyl]-2-yl)­diselane (**1bf**; 0.5 mmol, 0.276 g), TBAB (0.5 mmol, 0.161 g), LiClO_4_ (0.1 M, 0.106 g), and 10 mL of DMSO, and the crude product was then purified by column chromatography (SiO_2_, 10–15% ethyl acetate in hexanes) to provide **2bf** and **2bf′** (3:1) as a white solid; yield: 70% (193 mg; 0.5 mmol scale); mp = 71–72 °C. ^1^H NMR (CDCl_3_, 400 MHz): δ = 7.93–7.90 (m, 1H), 7.81–7.72 (m, 2H), 7.62 (d, *J* = 2.6 Hz, 1H), 7.22 (d, *J* = 8.0 Hz, 1H), 7.05–7.02 (m, 1H), 4.00 (s, 1H), 3.94 (s, 2H), 2.53 (s, 3H) ppm. ^13^C­{^1^H} NMR (CDCl_3_, 100 MHz): δ = 158.1, 156.5, 139.9, 139.4, 139.0, 138.4, 137.0, 136.3, 134.5, 130.7, 128.4, 128.4, 126.7, 126.4, 125.9, 125.9, 123.7, 123.3, 115.4, 106.8, 106.8, 55.8, 55.7, 21.5 ppm. HRMS (EI) calcd for C_14_H_12_OSe [M]^+^ 276.0053, found 276.0059.

#### 8-Methylbenzo­[*b*]­naphtho­[2,1-*d*]­selenophene (**2bg**)

It was synthesized following the general procedure using 1,2-bis­(4-methyl-2-(naphthalen-2-yl)­phenyl)­diselane (**1bg**; 0.5 mmol, 0.297 g), TBAB (0.5 mmol, 0.161 g), LiClO_4_ (0.1 M, 0.106 g), and 10 mL of DMSO, and the crude product was then purified by column chromatography (SiO_2_, 10–15% ethyl acetate in hexanes) to provide **2bg** as a white solid; yield: 79% (233 mg; 0.5 mmol scale); mp = 199–200 °C. ^1^H NMR (CDCl_3_, 400 MHz): δ = 8.11 (d, *J* = 8.4 Hz, 1H), 8.00 (s, 1H), 7.95 (d, *J* = 7.6 Hz, 2H), 7.86 (dd, *J* = 8.3 and 2.9 Hz, 2H), 7.61–7.53 (m, 2H), 7.25 (d, *J* = 8.0 Hz, 1H), 2.55 (s, 3H) ppm. ^13^C­{^1^H} NMR (CDCl_3_, 100 MHz): δ = 139.7, 139.7, 136.1, 135.6, 134.8, 132.5, 131.5, 128.9, 128.0, 126.9, 126.4 (2C), 125.9, 125.8, 123.5, 120.9, 21.6 ppm. HRMS (EI) calcd for C_17_H_12_Se [M]^+^ 296.0104, found 296.0098.

#### 2-Fluoro-7-methyldibenzo­[*b*,*d*]­selenophene (**2db**)

It was synthesized following the general procedure using 1,2-bis­(5-fluoro-4′-methyl-[1,1′-biphenyl]-2-yl)­diselane (**1db**; 0.5 mmol, 0.264 g), TBAB (0.5 mmol, 0.161 g), LiClO_4_ (0.1 M, 0.106 g), and 10 mL of DMSO, and the crude product was then purified by column chromatography (SiO_2_, 10–15% ethyl acetate in hexanes) to provide **2db** as a white solid; yield: 91% (239 mg; 0.5 mmol scale); mp = 94–96 °C. ^1^H NMR (CDCl_3_, 400 MHz): δ = 7.92 (d, *J* = 8.1 Hz, 1H), 7.78–7.72 (m, 2H), 7.67 (s, 1H), 7.26 (d, *J* = 8.2 Hz, 1H), 7.14–7.09 (m, 1H), 2.48 (s, 3H) ppm. ^13^C­{^1^H} NMR (CDCl_3_, 100 MHz): δ = 161.6 (d, *J*_CF_ = 241 Hz), 140.8, 139.9 (d, *J*_CF_ = 8 Hz), 137.8, 135.4 (d, *J*_CF_ = 3 Hz), 133.4, 127.1 (d, *J*_CF_ = 9 Hz), 126.5, 126.4, 122.9, 114.5 (d, *J*_CF_ = 24 Hz), 109.1 (d, *J*_CF_ = 23 Hz), 21.7 ppm. ^19^F NMR (CDCl_3_, 376 MHz): δ = – 118.05 ppm. HRMS (EI) calcd for C_13_H_9_FSe [M]^+^ 263.9854, found 263.9861.

#### 7-Chloro-2-fluorodibenzo­[*b*,*d*]­selenophene (**2dd**)

It was synthesized following the general procedure using 1,2-bis­(4′-chloro-5-fluoro-[1,1′-biphenyl]-2-yl)­diselane (**1dd**; 0.5 mmol, 0.285 g), TBAB (0.5 mmol, 0.161 g), LiClO_4_ (0.1 M, 0.106 g), and 10 mL of DMSO, and the crude product was then purified by column chromatography (SiO_2_, 10–15% ethyl acetate in hexanes) to provide **2dd** as a white solid; yield: 60% (170 mg; 0.5 mmol scale); mp = 83–85 °C. ^1^H NMR (CDCl_3_, 400 MHz): δ = 7.90 (d, *J* = 8.5 Hz, 1H), 7.82 (d, *J* = 2.0 Hz, 1H), 7.76 (dd, *J* = 8.7, 5.0 Hz, 1H), 7.70 (dd, *J* = 9.5 and 2.6 Hz, 1H), 7.40 (dd, *J* = 8.4 and 1.9 Hz, 1H), 7.15 (td, *J* = 8.7 and 2.5 Hz, 1H) ppm. ^13^C­{^1^H} NMR (CDCl_3_, 100 MHz): δ = 161.7 (d, *J*_CF_ = 242 Hz), 141.7, 138.9 (d, *J*_CF_ = 9 Hz), 136.2 (d, *J*_CF_ = 4 Hz), 133.8 (d, *J*_CF_ = 2 Hz), 133.4, 127.1 (d, *J*_CF_ = 9 Hz), 125.9, 125.8, 123.8, 115.3 (d, *J*_CF_ = 24 Hz), 109.4 (d, *J*_CF_ = 23 Hz) ppm. ^19^F NMR (CDCl_3_, 376 MHz): δ = −117.35 ppm. HRMS (EI) calcd for C_12_H_6_ClFSe [M]^+^ 283.9307, found 283.9312.

#### 8-Fluorobenzo­[*b*]­naphtho­[2,1-*d*]­selenophene (**2dg**)

It was synthesized following the general procedure using 1,2-bis­(4-fluoro-2-(naphthalen-2-yl)­phenyl)­diselane (**1dg**; 0.5 mmol, 0.300 g), TBAB (0.5 mmol, 0.161 g), LiClO_4_ (0.1 M, 0.106 g), and 10 mL of DMSO, and the crude product was then purified by column chromatography (SiO_2_, 10–15% ethyl acetate in hexanes) to provide **2dg** as a brown solid; yield: 75% (224 mg; 0.5 mmol scale); mp = 175–177 °C. ^1^H NMR (CDCl_3_, 400 MHz): δ = 8.03 (d, *J* = 8.6 Hz, 1H), 7.96–7.82 (m, 5H), 7.62–7.55 (m, 2H), 7.19–7.14 (m, 1H) ppm. ^13^C­{^1^H} NMR (CDCl_3_, 100 MHz): δ = 161.7 (d, *J*_CF_ = 241 Hz), 141.0 (d, *J*_CF_ = 16 Hz), 135.0 (d, *J*_CF_ = 4 Hz), 133.8 (d, *J*_CF_ = 2 Hz), 132.6, 131.4, 129.0, 127.2, 127.2, 127.2, 126.9, 126.4, 126.3, 120.9, 114.7 (d, *J*_CF_ = 25 Hz), 109.5 (d, *J*_CF_ = 23 Hz) ppm. ^19^F NMR (CDCl_3_, 376 MHz): δ = −117.77 ppm. HRMS (EI) calcd for C_16_H_9_FSe [M]^+^ 299.9854, found 299.9860.

#### 2-Chloro-7-methyldibenzo­[*b*,*d*]­selenophene (**2eb**)

It was synthesized following the general procedure using 1,2-bis­(5-chloro-4′-methyl-[1,1′-biphenyl]-2-yl)­diselane (**1eb**; 0.5 mmol, 0.281 g), TBAB (0.5 mmol, 0.161 g), LiClO_4_ (0.1 M, 0.106 g), and 10 mL of DMSO, and the crude product was then purified by column chromatography (SiO_2_, 10–15% ethyl acetate in hexanes) to provide **2eb** as a white solid; yield: 85% (238 mg; 0.5 mmol scale); mp = 74–75 °C. ^1^H NMR (CDCl_3_, 400 MHz): δ = 8.01 (d, *J* = 2.1 Hz, 1H), 7.90 (d, *J* = 8.1 Hz, 1H), 7.73 (d, *J* = 8.4 Hz, 1H), 7.65 (s, 1H), 7.31 (dd, *J* = 8.4 and 2.1 Hz, 1H), 7.25 (d, *J* = 8.0 Hz, 1H), 2.48 (s, 3H) ppm. ^13^C­{^1^H} NMR (CDCl_3_, 100 MHz): δ = 140.3, 139.9, 137.8, 136.9, 134.9, 131.3, 127.0, 126.6, 126.5, 126.3, 122.7, 122.5, 21.7 ppm. HRMS (EI) calcd for C_13_H_9_ClSe [M]^+^ 279.9558, found 279.9548.

#### 2-Bromo-7-methyldibenzo­[*b*,*d*]­selenophene (**2fb**)

It was synthesized following the general procedure using 1,2-bis­(5-bromo-4′-methyl-[1,1′-biphenyl]-2-yl)­diselane (**1fb**; 0.5 mmol, 0.325 g), TBAB (0.5 mmol, 0.161 g), LiClO_4_ (0.1 M, 0.106 g), and 10 mL of DMSO, and the crude product was then purified by column chromatography (SiO_2_, 10–15% ethyl acetate in hexanes) to provide **2fb** as a white solid; yield: 80% (259 mg; 0.5 mmol scale); mp = 69–71 °C. ^1^H NMR (CDCl_3_, 400 MHz): δ = 8.18 (d, *J* = 2.0 Hz, 1H), 7.95 (d, *J* = 8.1 Hz, 1H), 7.70 (d, *J* = 8.4 Hz, 1H), 7.68 (s, 1H), 7.45 (dd, *J* = 8.4 and 2.0 Hz, 1H), 7.29–7.26 (m, 1H), 2.48 (s, 3H) ppm. ^13^C­{^1^H} NMR (CDCl_3_, 100 MHz): δ = 140.4, 137.9, 137.6, 134.8, 129.3, 127.5, 126.7, 126.3, 125.6, 122.8, 119.1, 21.7 ppm. HRMS (EI) calcd for C_13_H_9_BrSe [M]^+^ 323.9053, found 323.9055.

#### 7-Methyl-2-(trifluoromethyl)­dibenzo­[*b*,*d*]­selenophene (**2gb**)

It was synthesized following the general procedure using 1,2-bis­(4′-methyl-5-(trifluoromethyl)-[1,1′-biphenyl]-2-yl)­diselane (**1gb**; 0.5 mmol, 0.314 g), TBAB (0.5 mmol, 0.161 g), LiClO_4_ (0.1 M, 0.106 g), and 10 mL of DMSO, and the crude product was then purified by column chromatography (SiO_2_, 10–15% ethyl acetate in hexanes) to provide **2gb** as a white solid; yield: 95% (282 mg; 0.5 mmol scale); mp = 75–76 °C. ^1^H NMR (CDCl_3_, 400 MHz): δ = 8.26 (d, *J* = 1.9 Hz, 1H), 7.97 (d, *J* = 8.1 Hz, 1H), 7.90 (d, *J* = 8.3 Hz, 1H), 7.64 (s, 1H), 7.55 (d, *J* = 8.3 Hz, 1H), 7.25 (d, *J* = 8.0 Hz, 1H), 2.46 (s, 3H) ppm. ^13^C­{^1^H} NMR (CDCl_3_, 100 MHz): δ = 143.0, 140.2, 138.5, 138.1, 134.9, 127.5 (d, *J*_CF_ = 32 Hz), 126.8, 126.5, 126.2, 124.8 (d, *J*_CF_ = 270 Hz, *C*F_3_), 122.8, 122.6–122.5 (m), 119.4–119.3 (m), 21.6 ppm. ^19^F NMR (CDCl_3_, 376 MHz): δ = – 61.49 ppm. HRMS (EI) calcd for C_14_H_9_F_3_Se [M]^+^ 313.9822, found 313.9823.

## Supplementary Material



## Data Availability

The data underlying this study are available in the published article and its Supporting Information.

## Notes

Experimental procedures and the scanned copies of ^1^H NMR, ^13^C NMR, ^19^F NMR (for **2da**, **2ga**, **2be**, **2db**, **2dd**, **2dg**, and **2gb**), and those of cyclic voltammograms, along with single X-ray crystallographic information for **2bf**, are supplemented (PDF)
