# Supplementary material for: Site-Selective Electrochemical Intramolecular C(sp2)–H Selenylation for Dibenzoselenophene Synthesis
Source: J Org Chem. 2025 Aug 18;90(34):12205–15. doi: 10.1021/acs.joc.5c01600 (PMC12400415; doi:10.1021/acs.joc.5c01600)
Supplement: Supplementary file 1 [file jo5c01600_si_001.pdf]

Supporting Information

Site-Selective Electrochemical Intramolecular C(sp^2)–H Selenylation for Dibenzoselenophene Synthesis

Indrajit Karmakar,[a] Yi-Xuan Jian,[a] Wan-Lin Cheng,[a] Rekha Bai,[a] Yu-Hsuan Chen[a] and Chin-Fa Lee*[a,b,c]

[a]*Department of Chemistry, National Chung Hsing University, Taichung, Taiwan 402, Republic of China.*

[b]*i-Center for Advanced Science and Technology (iCAST), National Chung Hsing University, Taichung, Taiwan 402, Republic of China*

[c]*Innovation and Development Center of Sustainable Agriculture (IDCSA), National Chung Hsing University, Taichung, Taiwan 402, Republic of China*

*Corresponding author. E-mail: cfalee@dragon.nchu.edu.tw

ORCID: http://orcid.org/0000-0003-0735-5691

Table of Contents

1. General……………………...………………………..…………………………....…..……………..…....…S1

2. General procedure for the synthesis of dibenzoselenophenes in the presence of an electrochemical cell...S1

3. Large-scale synthesis of compounds **2aa** and **2gb** under an electrochemical cell………..…..………..…S1

4. Pictorial views of the experimental Setup.……………………………………………………………..…. S2

5. General procedure for cyclic voltammetry……………………………………………………………..…. S2

6. Cyclic voltammograms of 1,2-di([1,1′-biphenyl]-2-yl)diselane (**1aa**), TBAB, $LiClO_4$, $^nBu_4NBF_4$ and other mixtures (Figure **S2** and **S3**)……………...…………………………………..…..………………………S3

7. Single X-ray crystal structure analysis of 2-methoxy-8-methyldibenzo[*b*,*d*]selenophene (**2bf**) (ORTEP view Figure **S4** and Table **S1**)……………………………………………………………………………...…S4-S5

8. Scanned copies of ^{1}H NMR, ^{13}C NMR, ^{19}F NMR (for **2da**, **2ga**, **2be**, **2db**, **2dd**, **2dg** and **2gb**) spectra for all the synthesized dibenzoselenophenes **2** (**2aa**–**2gb**) (Figure **S5** – **S49**); HRMS spectrum of intermediate **3** (Figure **S50** – **S51**)……………..….……………………………………………………..…...……S6-S52

9. References……………………………………………………………………………………….…..S53

1. General. All solvents used in this study were distilled and dried before use, according to standard procedures. ^{1}H, ^{13}C and ^{19}F NMR spectra were collected at Varian Unity Inova-600 or a Varian Mercury-400 NMR instrument using $CDCl_3$ as solvent. Chemical shifts were reported in δ (ppm), relative to the internal standard, TMS. The signals observed are described as s (singlet), d (doublet), t (triplet), and m (multiplet). Coupling constants are reported as J values in Hz. Mass spectrometry was obtained using a Jeol JMS-HX 110 spectrometer. The X-ray diffraction measurements were carried out using a Bruker D8 VENTURE XRD instrument. Cyclic voltammetry was performed on a CHI Instruments 750A potentiostat using acetonitrile as solvent. The melting points were recorded on a Büchi 535 melting point apparatus and are uncorrected. Thin Layer Chromatography (TLC) was performed using silica gel 60 F_{254} (Merck) plates. An undivided electrochemical cell equipped with platinum electrodes (IKA) was used, with dimensions of 0.7 cm × 0.7 cm × 0.2 cm for small-scale reactions and 2.8 cm × 0.7 cm × 0.2 cm for larger-scale reactions. A GW Instek GPS-2303 Laboratory DC Power Supply (350 watts, 450 VA, 50/60 Hz) was employed as the power source.

2. General procedure for the synthesis of dibenzoselenophenes in the presence of an electrochemical cell

An oven-dried glass vessel was sequentially charged with bis(biaryl) diselenides (**1**; 0.5 mmol), TBAB (0.5 mmol), 10 mL of a 0.1 M $LiClO_4$ electrolyte solution in DMSO, and a magnetic stir bar. The vessel was then sealed with a pair of platinum plate electrodes (each measuring 0.7 cm × 0.7 cm × 0.2 cm) positioned 0.5 cm apart, forming an undivided electrochemical cell. A constant direct current of 10 mA was applied across the reaction mixture while maintaining a temperature of 70 °C. The mixture was stirred continuously at ambient pressure for 3 hours. The progress of the reaction was monitored by TLC. Upon completion of the reaction, 20 mL of a 3:1 (*v*/*v*) mixture of ethyl acetate and water was added to the reaction mixture and transferred to a separating funnel. The mixture was thoroughly shaken, and the organic layer was separated and dried over anhydrous sodium sulfate. The solvent was then removed under reduced pressure, and the resulting crude product was purified by column chromatography using ethyl acetate-hexane mixtures as eluents. This process afforded the desired product dibenzoselenophenes **2** (**2aa**-**2gb**). All synthesized compounds were fully characterized by detailed spectroscopic analyses, including ^{1}H NMR, ^{13}C NMR, ^{19}F NMR, and HRMS spectrometry.

3. Large-scale synthesis of compounds 2aa and 2gb under an electrochemical cell

An oven-dried glass vessel was sequentially charged with 1,2-di([1,1′-biphenyl]-2-yl)diselane/1,2-bis(4'-methyl-5-(trifluoromethyl)-[1,1'-biphenyl]-2-yl)diselane (**1aa/1gb**; 2.5 mmol, 1.160 g/1.570 g), TBAB (2.5 mmol, 0.805 g), 50 mL of a 0.1 M $LiClO_4$ (0.530 g) electrolyte solution in DMSO, and a magnetic stir bar. The vessel was then sealed with a pair of platinum plate electrodes (each measuring 2.8 cm × 0.7 cm × 0.2 cm) positioned 0.5 cm apart, forming an undivided electrochemical cell. A constant direct current of 20 mA was applied across the reaction mixture while maintaining a temperature of 70 °C. The mixture was stirred continuously at ambient pressure for 7 hours. The progress of the reaction was monitored by TLC. Upon completion of the reaction, 80 mL of a 3:1 (*v*/*v*) mixture of ethyl acetate and water was added to the reaction mixture and transferred to a separating funnel. The mixture was thoroughly shaken, and the organic layer was separated and dried over anhydrous sodium sulfate. The solvent was then removed under reduced pressure, and the resulting crude product was purified by column chromatography using ethyl acetate-hexane mixtures as eluents. This process afforded the desired product dibenzo[*b*,*d*]selenophene/7-methyl-2-(trifluoromethyl)dibenzo[*b*,*d*]selenophene (**2aa/2gb**) with 73%/77% yield (0.843 g/1.266 g).

4. Pictorial views of the experimental Setup

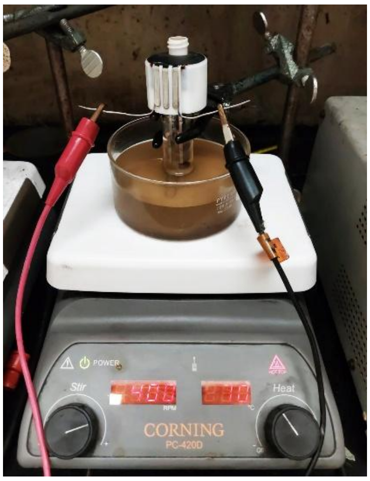

(a)

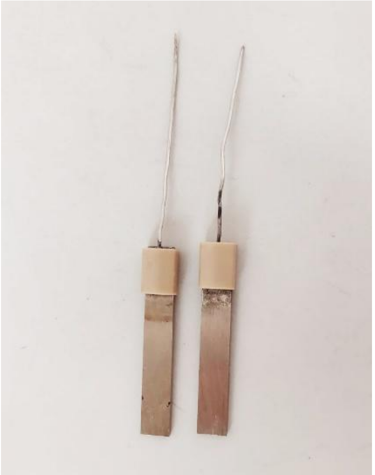

(b)

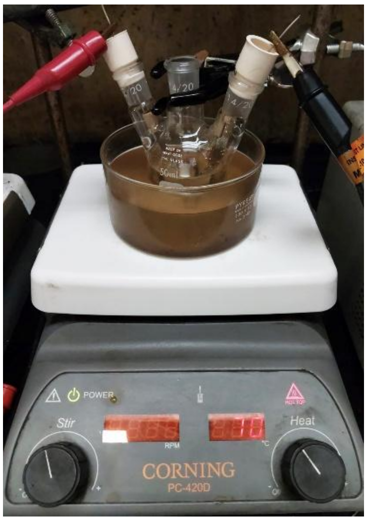

(c)

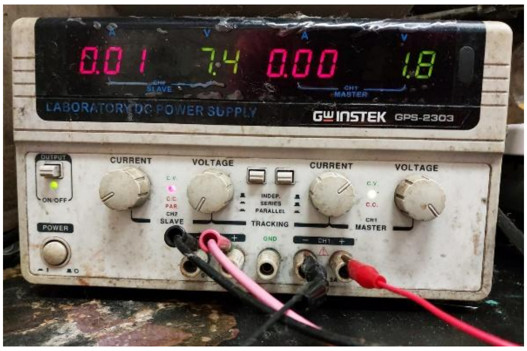

(d)

Figure S1: (a) Small scale experimental setup; (b) IKA made Platinum/Platinum plate electrodes; (c) Larger-scale experimental setup (d)'DC Regulated Power Supply'

5. General procedure for cyclic voltammetry

Electrochemical tests were performed on a CHI Instruments 750A potentiostat using acetonitrile as solvent. A standard cyclic voltammetric (CV) experiment based on a three-electrode system is conducted. The working electrode uses BAS glassy carbon (3 mm diameter), while the reference and auxiliary electrodes use Ag/AgCl (saturated) and platinum wire, respectively. Potentials are reported *vs*. Ag/AgCl (saturated). The working electrode was polished with 0.03 μm aluminium on felt pads (Buehler) before each experiment. The reference is Ag/Ag^+ electrode, and 10 mL of electrolyte solution containing 300 mg $^n Bu_4NBF_4$ in CH_3CN was poured into the electrochemical cell in all experiments. 1 mg of each of the samples, such as 1,2-di([1,1′-biphenyl]-2-yl)diselane (**1aa**), TBAB (**2e**), $LiClO_4$ and other mixtures, was used for the purpose. The potential scan ranged from −2.0 to +2.0 V at a scan rate of 0.1 V/s for each case. CV plotting convention is IUPAC.

6. **Cyclic voltammograms of 1,2-di([1,1′-biphenyl]-2-yl)diselane (1aa), TBAB, $LiClO_4$, $^{n}Bu_4NBF_4$ and other mixtures:**

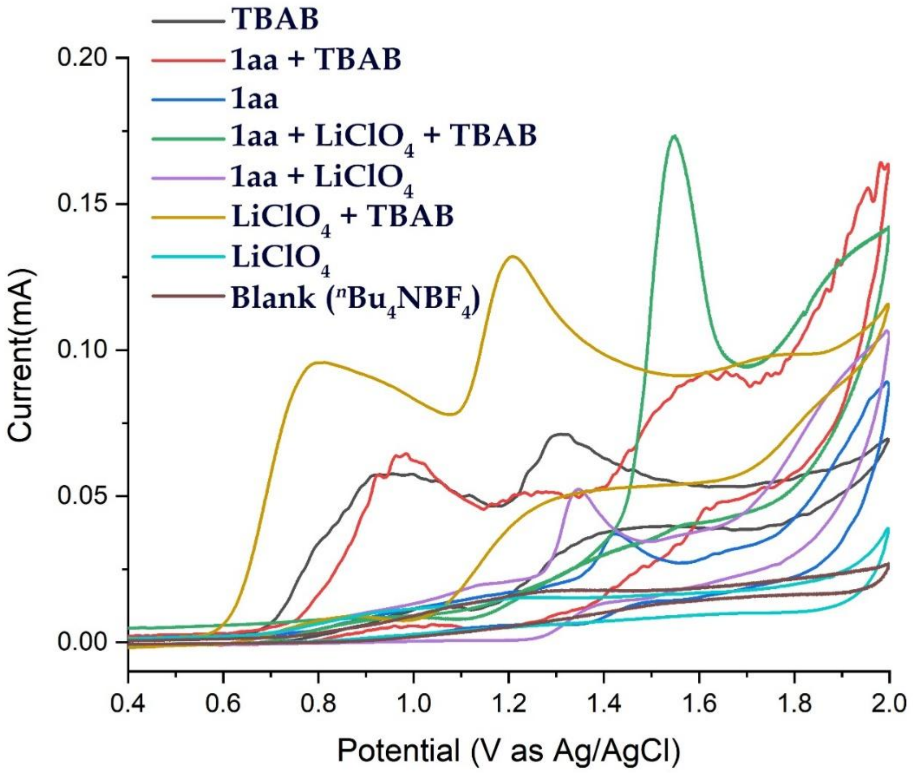

Figure S2. Cyclic voltammetry; CV plotting convension: IUPAC; Working electrode: BAS glassy carbon; Counter electrode: Platinum wire; Reference electrodes: Ag/Ag^+ electrode; Solvent: CH_3CN (10 mL); Electrolyte: ^{n}**Bu₄NBF₄** (0.09 M); Analyte: **1aa** (0.0002 M); **$LiClO_4$** (0.0009 M); **TBAB** (0.0003 M); Temperature: Room temperature; Starting point: 0.0; Direction of scan: + Direction; The potential scan ranged from –2.0 to +2.0 V at a scan rate of 0.1 V/s for each cases.

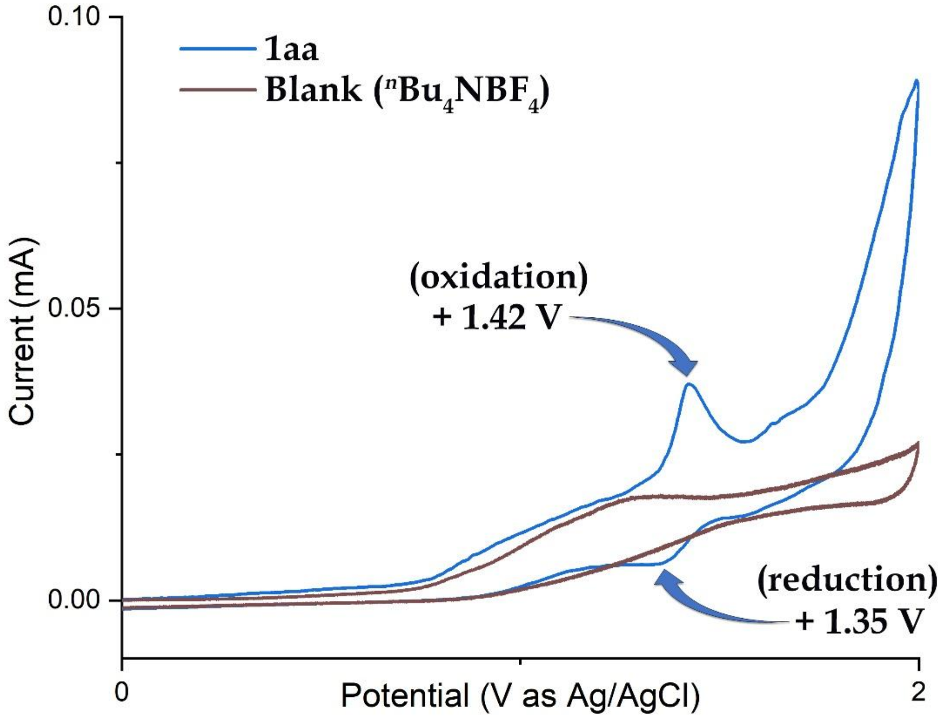

Figure S3. Cyclic voltammetry of **1aa**; Solvent: CH_3CN (10 mL); Electrolyte: ^{n}**Bu_4NBF_4** (0.09 M); Analyte: **1aa** (0.0002 M).

7. Single X-ray crystal structure analysis of 2-methoxy-8-methyldibenzo[b,d]selenophene (2bf)

Preparation of single crystals of compound 2bf

For preparing single crystals of compound **2bf**, 50 mg of the sample was dissolved in 5 mL of commercial chloroform, and the solution was left for 15 days for slow evaporation at ambient temperature to yield colourless block-shaped crystals.

CCDC 2450651 (Compound **2bf**) contains the supplementary crystallographic data for this paper. These data can be obtained free of charge from The Cambridge Crystallographic Data Centre *via* www.ccdc.cam.ac.uk/data_request/cif

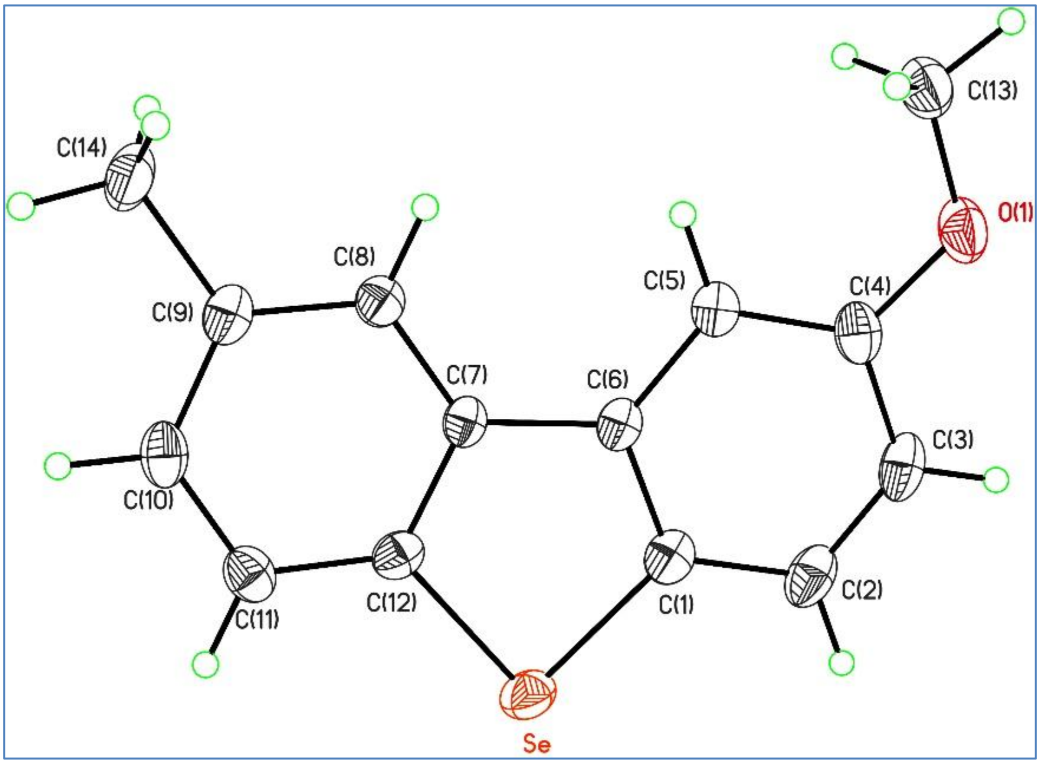

Figure S4 *ORTEP* view of the molecule, showing the atom-labelling scheme
Displacement ellipsoids are drawn at the 50% probability level and H atoms are shown as small spheres of arbitrary radii.

Table S1. Crystal data and structure refinement for 2-methoxy-8-methyldibenzo[b,d]selenophene (2bf)

Empirical formula	$C_{14} H_{12} O Se$	
Formula weight	275.20	
Temperature	150(2) K	
Wavelength	0.71073 Å	
Crystal system	Orthorhombic	
Space group	$P2_12_12_1$	
Unit cell dimensions	a = 6.3622(3) Å	α= 90°.
	b = 8.8554(5) Å	β= 90°.
	c = 20.2197(11) Å	γ = 90°.
Volume	1139.17(10) Å^3	

Z	4
Density (calculated)	1.605 Mg/m^3
Absorption coefficient	3.269 mm^{-1}
F(000)	552
Crystal size	0.450 x 0.210 x 0.110 mm^3
Theta range for data collection	3.058 to 27.892°.
Index ranges	-8<=h<=8, -11<=k<=11, -26<=l<=26
Reflections collected	42112
Independent reflections	2733 [R(int) = 0.0350]
Completeness to theta = 25.242°	99.8 %
Absorption correction	Semi-empirical from equivalents
Max. and min. transmission	0.7456 and 0.5172
Refinement method	Full-matrix least-squares on F^2
Data / restraints / parameters	2733 / 0 / 145
Goodness-of-fit on F^2	1.080
Final R indices [I>2sigma(I)]	R1 = 0.0193, wR2 = 0.0479
R indices (all data)	R1 = 0.0210, wR2 = 0.0486
Absolute structure parameter	0.036(4)
Extinction coefficient	n/a
Largest diff. peak and hole	0.484 and -0.326 e.Å^{-3}
Software for solve and refine	Olex2[1]
Software for structure solution	SHELXS[2]
Software for refinement	SHELXL[3]

8. **Scanned copies of ^{1}H NMR, ^{13}C NMR, ^{19}F NMR (for 2da, 2ga, 2be, 2db, 2dd, 2dg, and 2gb) spectra for all the synthesized dibenzoselenophenes 2 (2aa–2gb) (Figure S5 – S49); HRMS spectrum of intermediate 3 (Figure S50 – S51)**

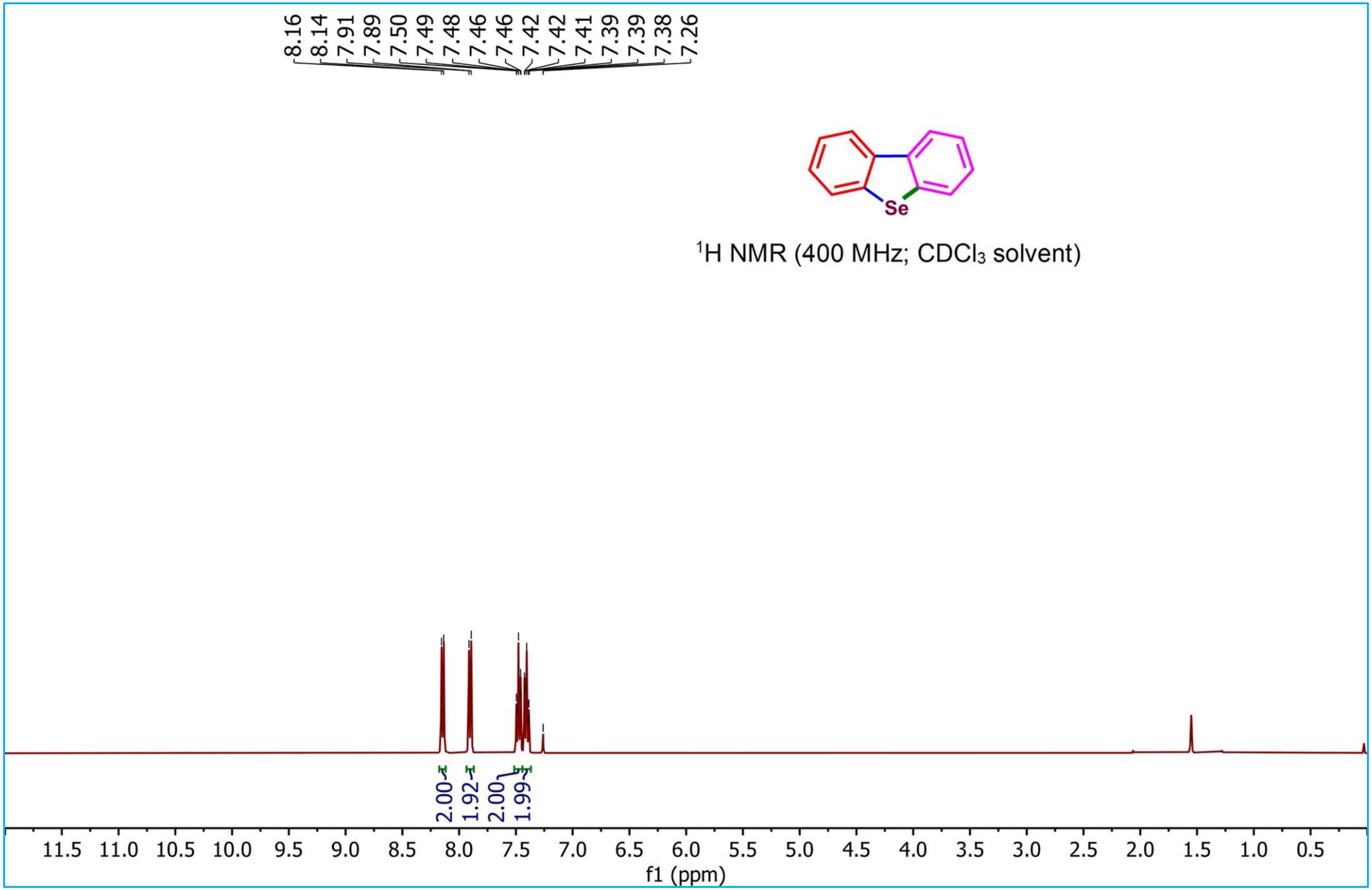

Figure S5. ^{1}H NMR spectrum of dibenzo[b,d]selenophene (**2aa**)

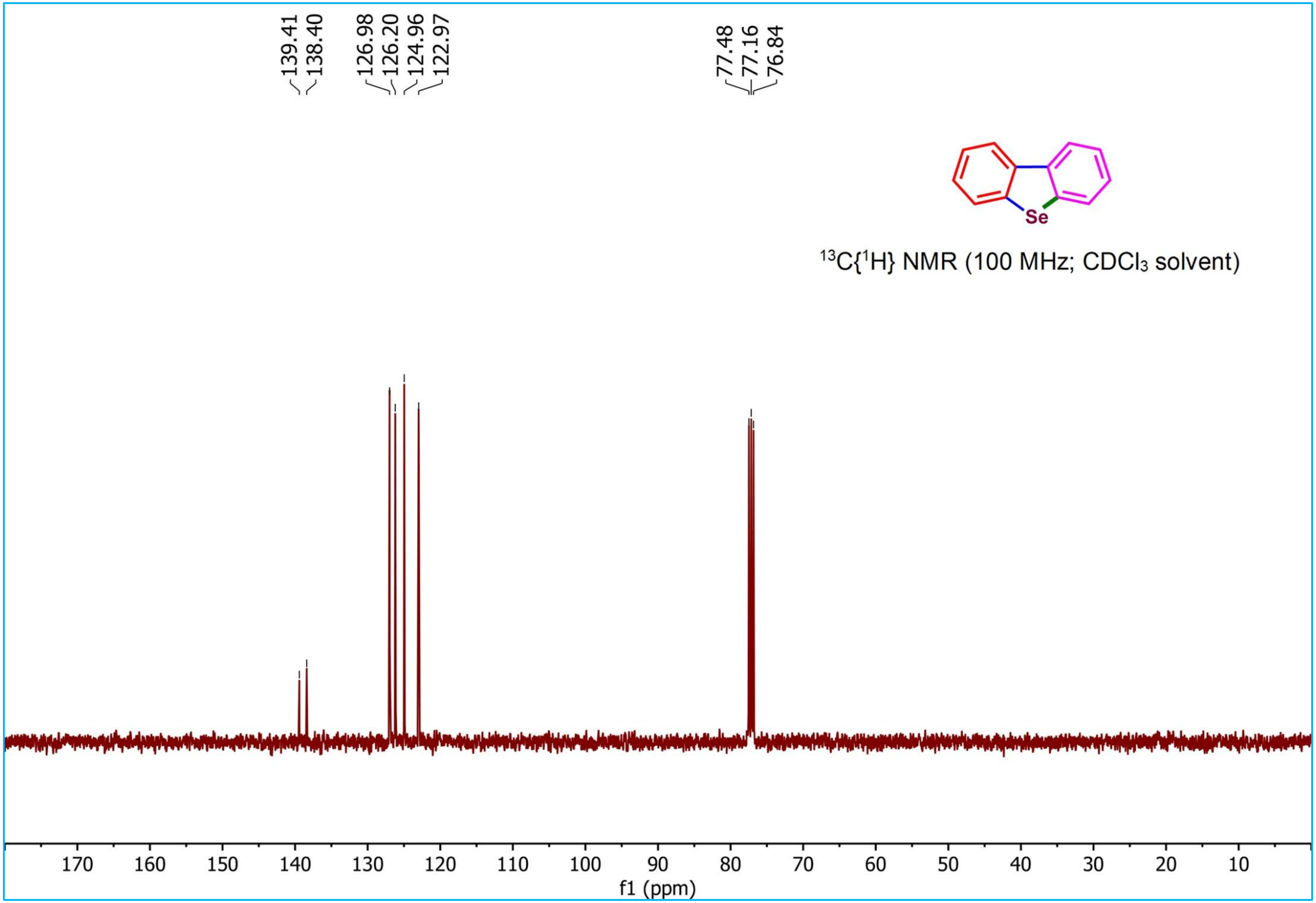

Figure S6. $^{13}C\{^{1}H\}$ NMR spectrum of dibenzo[b,d]selenophene (**2aa**)

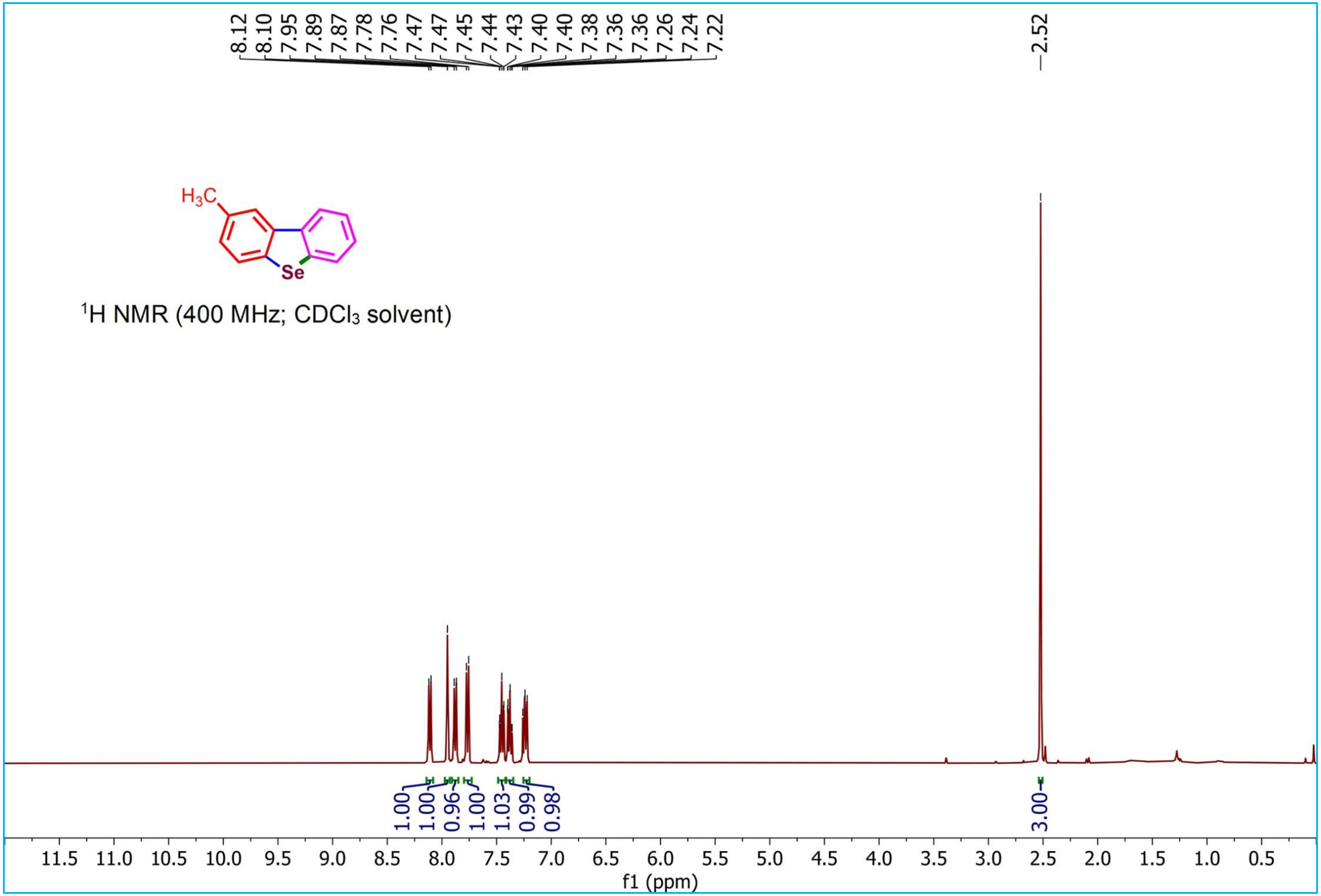

Figure S7. ^{1}H NMR spectrum of 2-methyldibenzo[b,d]selenophene (**2ba**)

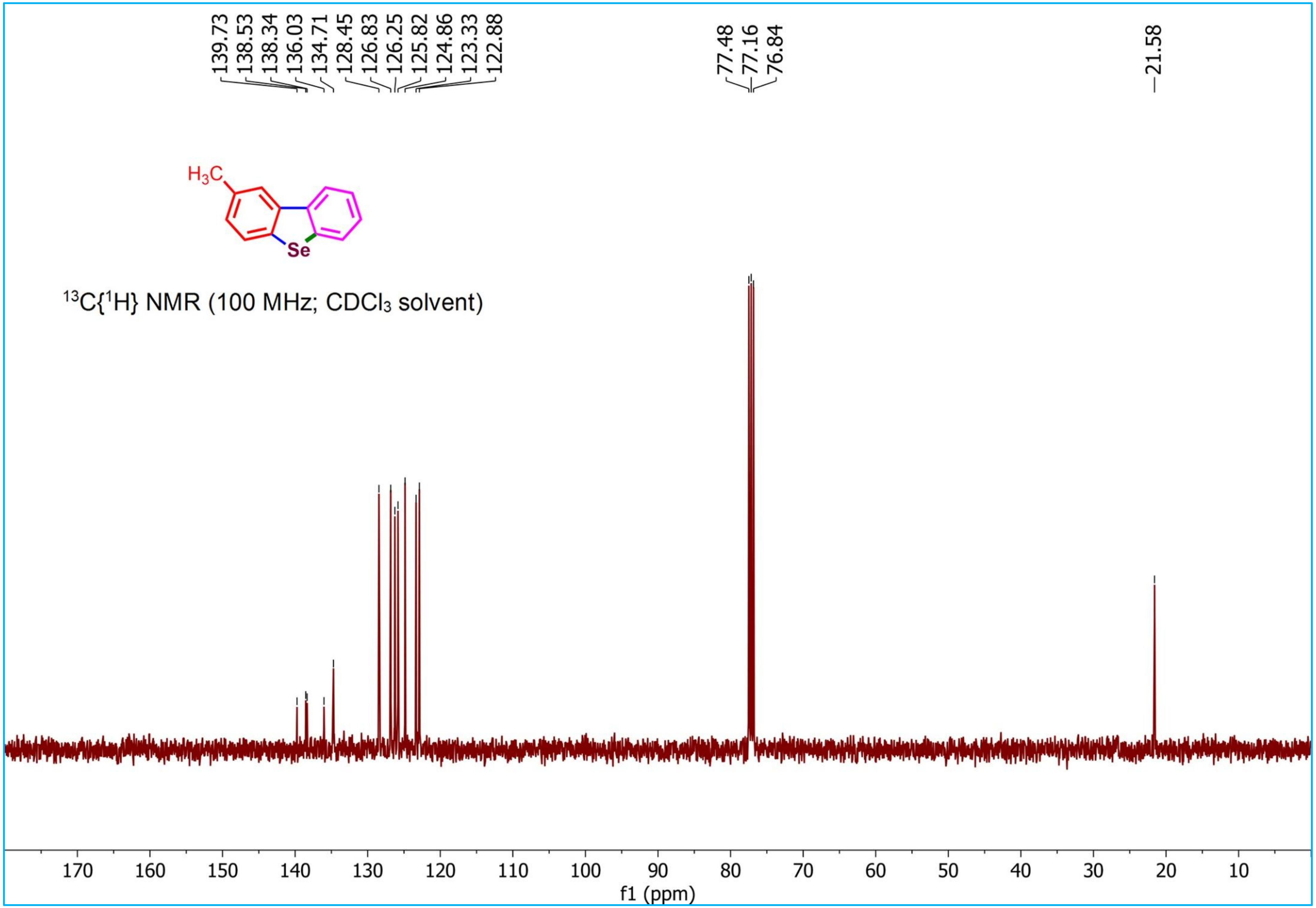

Figure S8. $^{13}C\{^{1}H\}$ NMR spectrum of 2-methyldibenzo[b,d]selenophene (**2ba**)

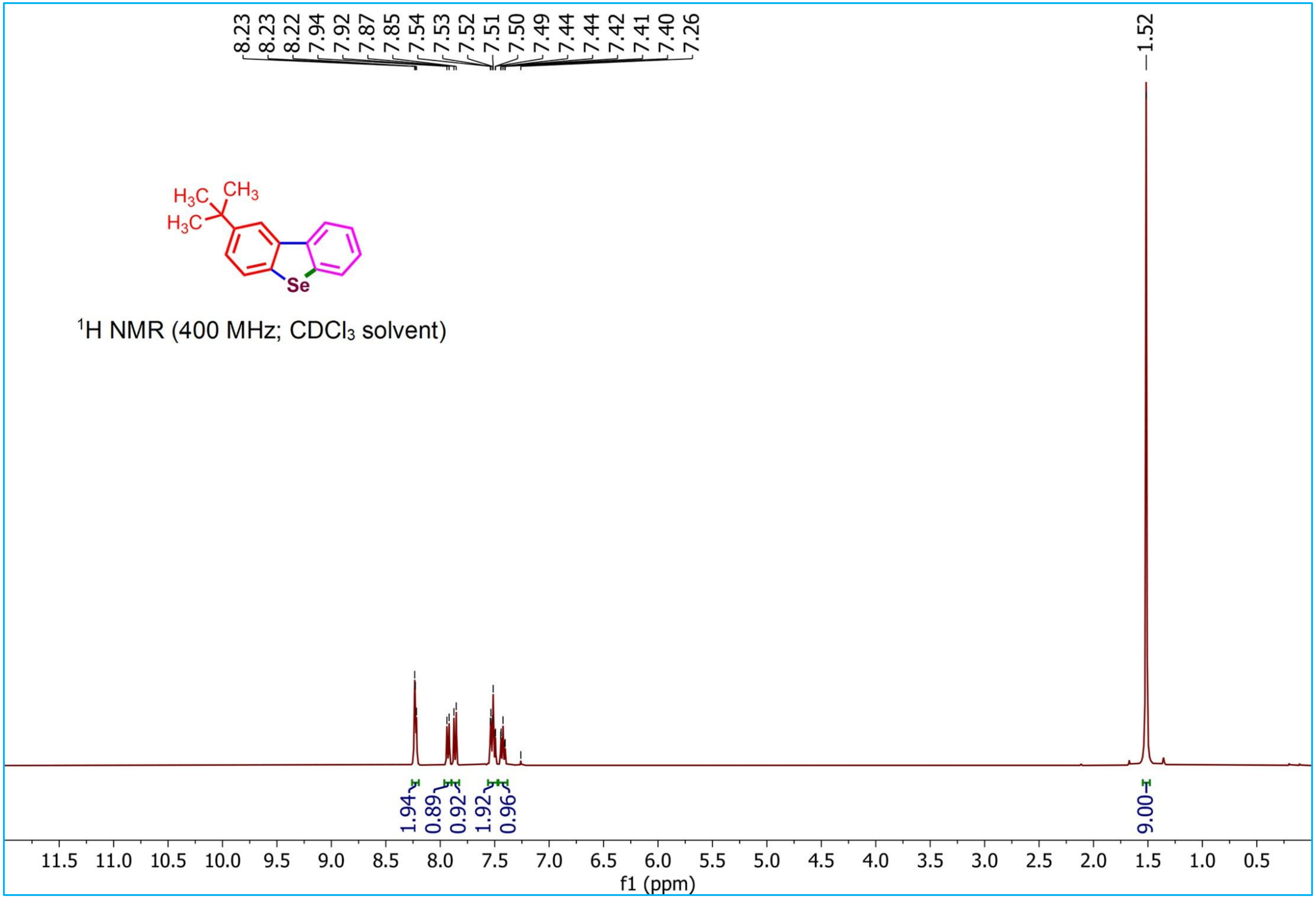

Figure S9. ^{1}H NMR spectrum of 2-(*tert*-butyl)dibenzo[b,d]selenophene (**2ca**)

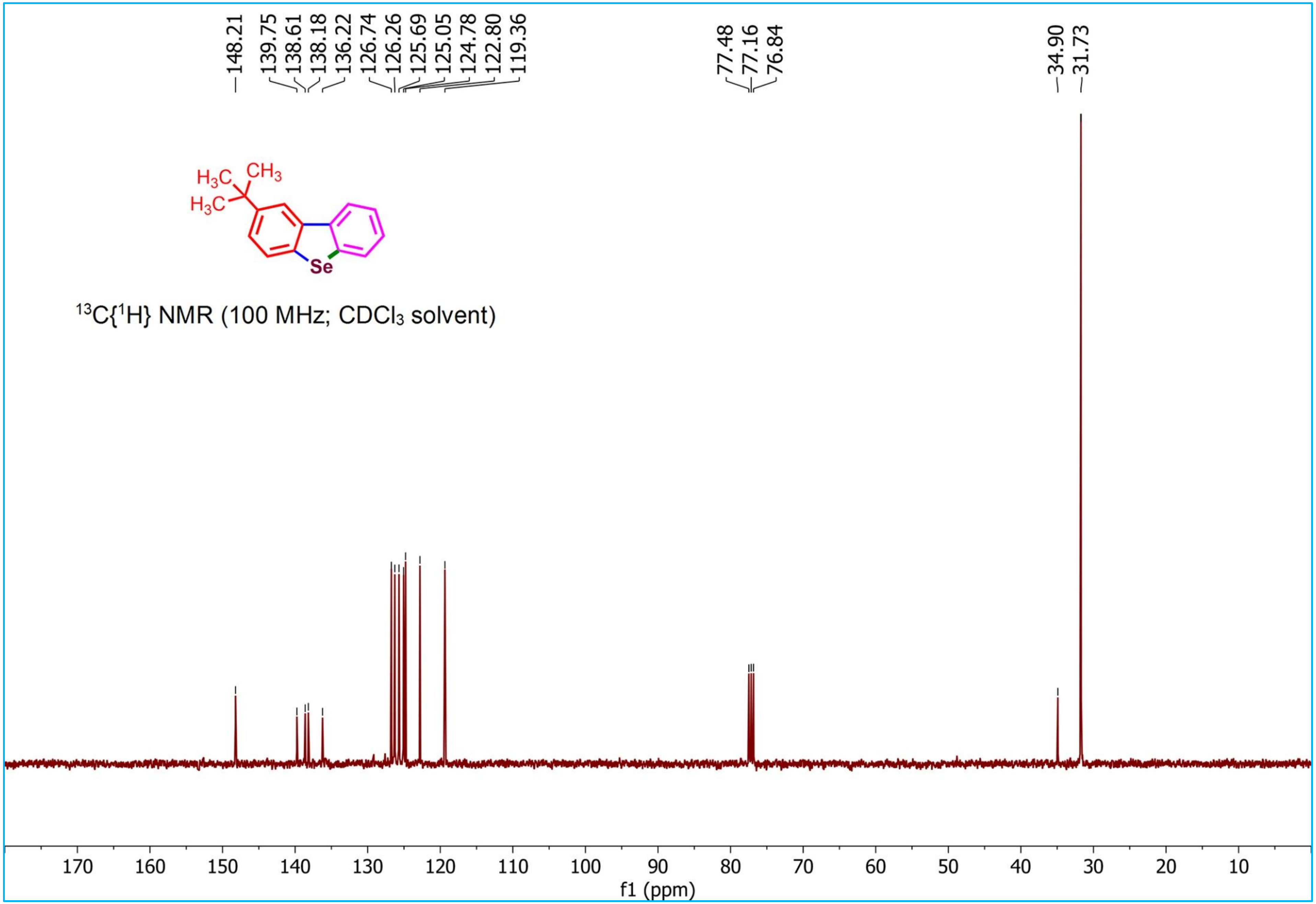

Figure S10. $^{13}C\{^{1}H\}$ NMR spectrum of 2-(*tert*-butyl)dibenzo[b,d]selenophene (**2ca**)

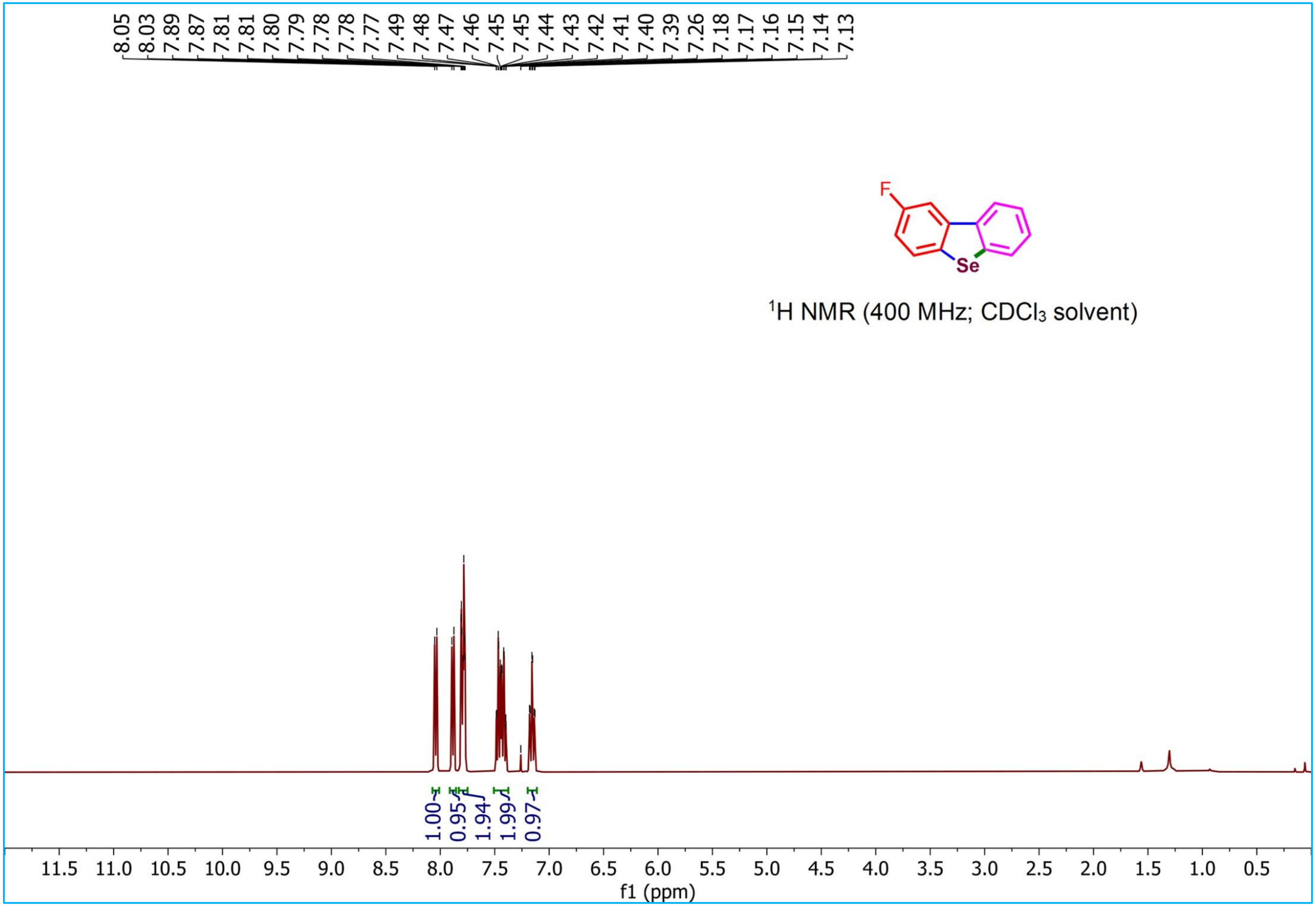

Figure S11. ^{1}H NMR spectrum of 2-fluorodibenzo[b,d]selenophene (**2da**)

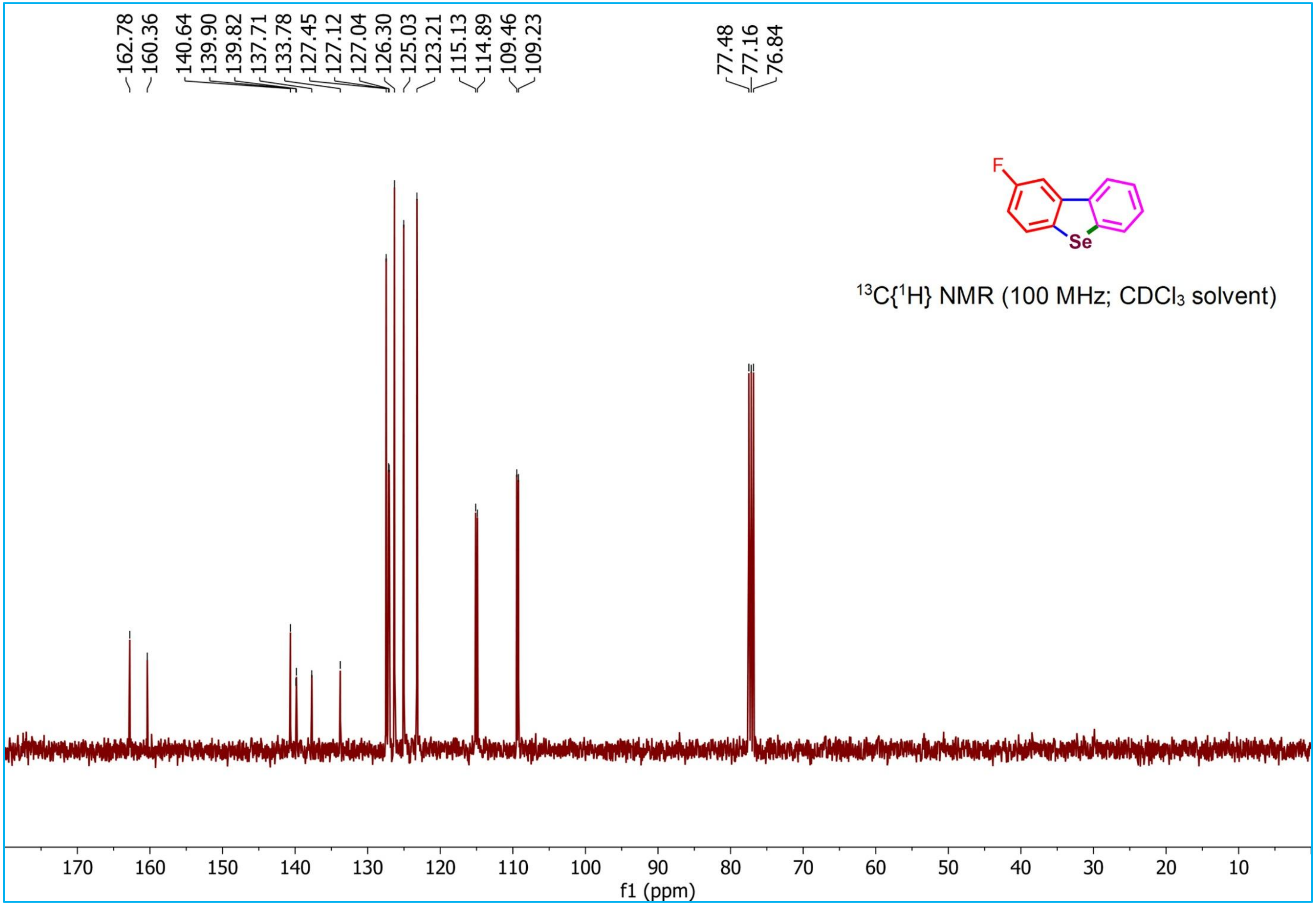

Figure S12. ^{13}C{^{1}H} NMR spectrum of 2-fluorodibenzo[b,d]selenophene (**2da**)

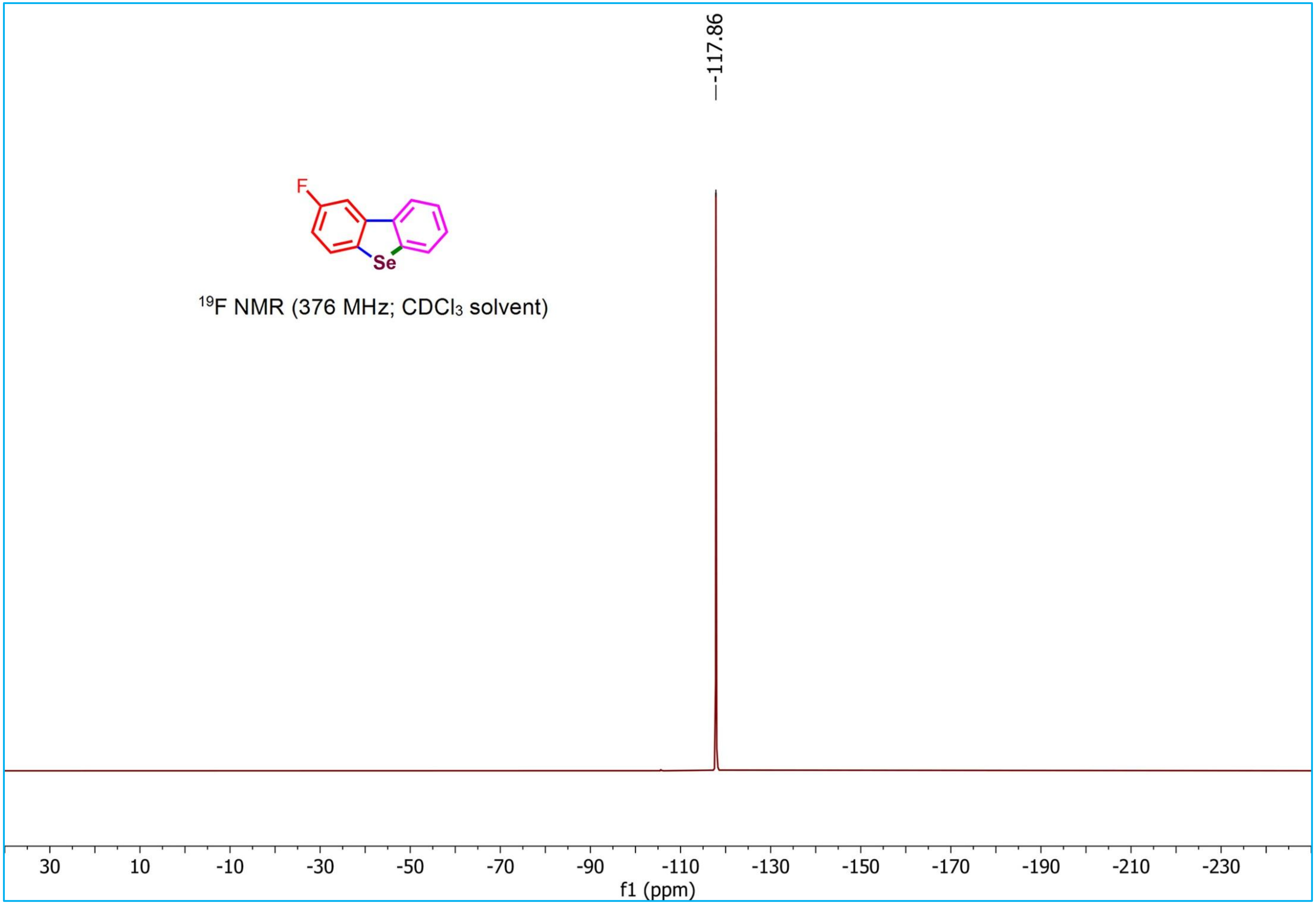

Figure S13. ^{19}F NMR spectrum of 2-fluorodibenzo[b,d]selenophene (**2da**)

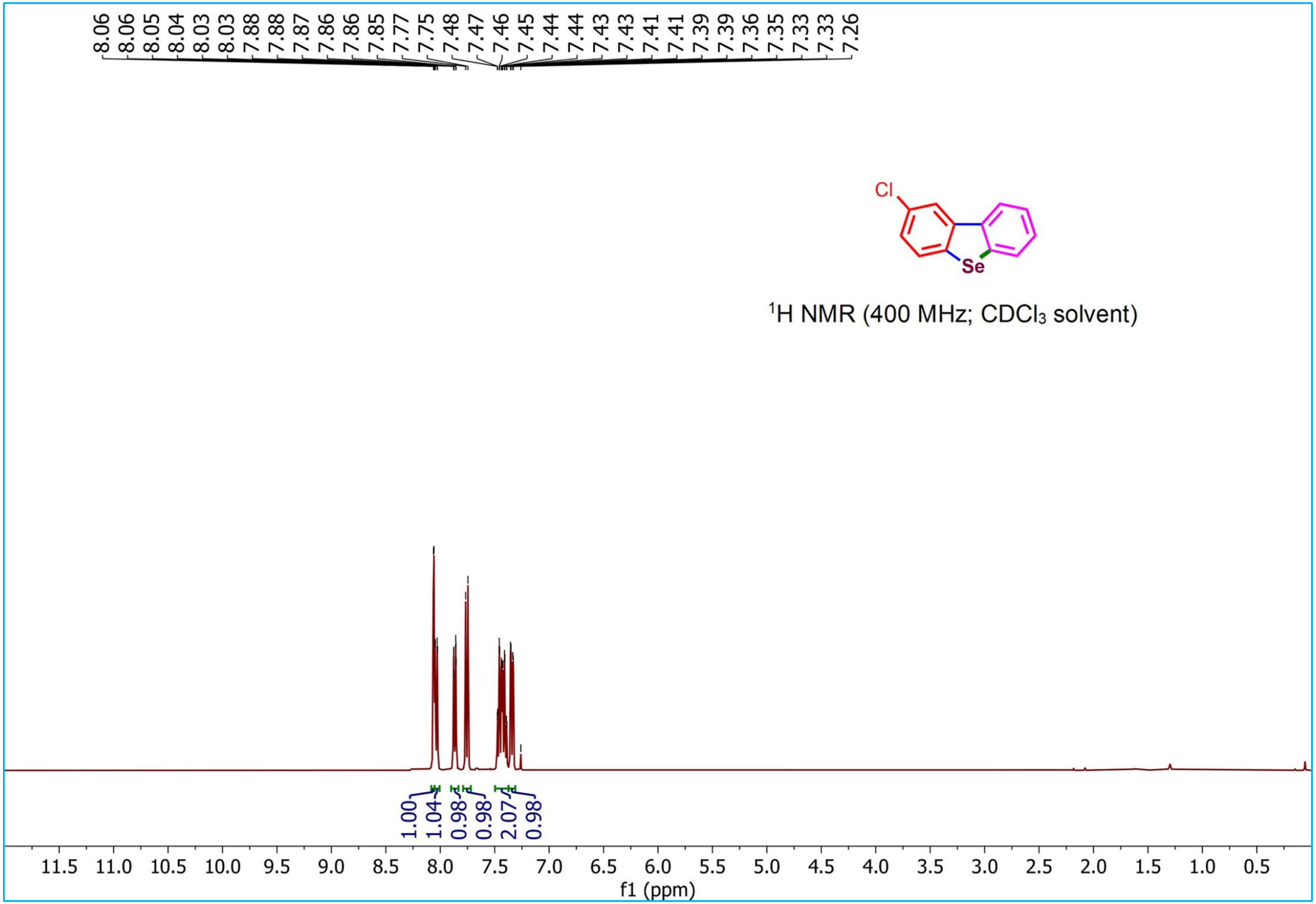

Figure S14. ^{1}H NMR spectrum of 2-chlorodibenzo[b,d]selenophene (**2ea**)

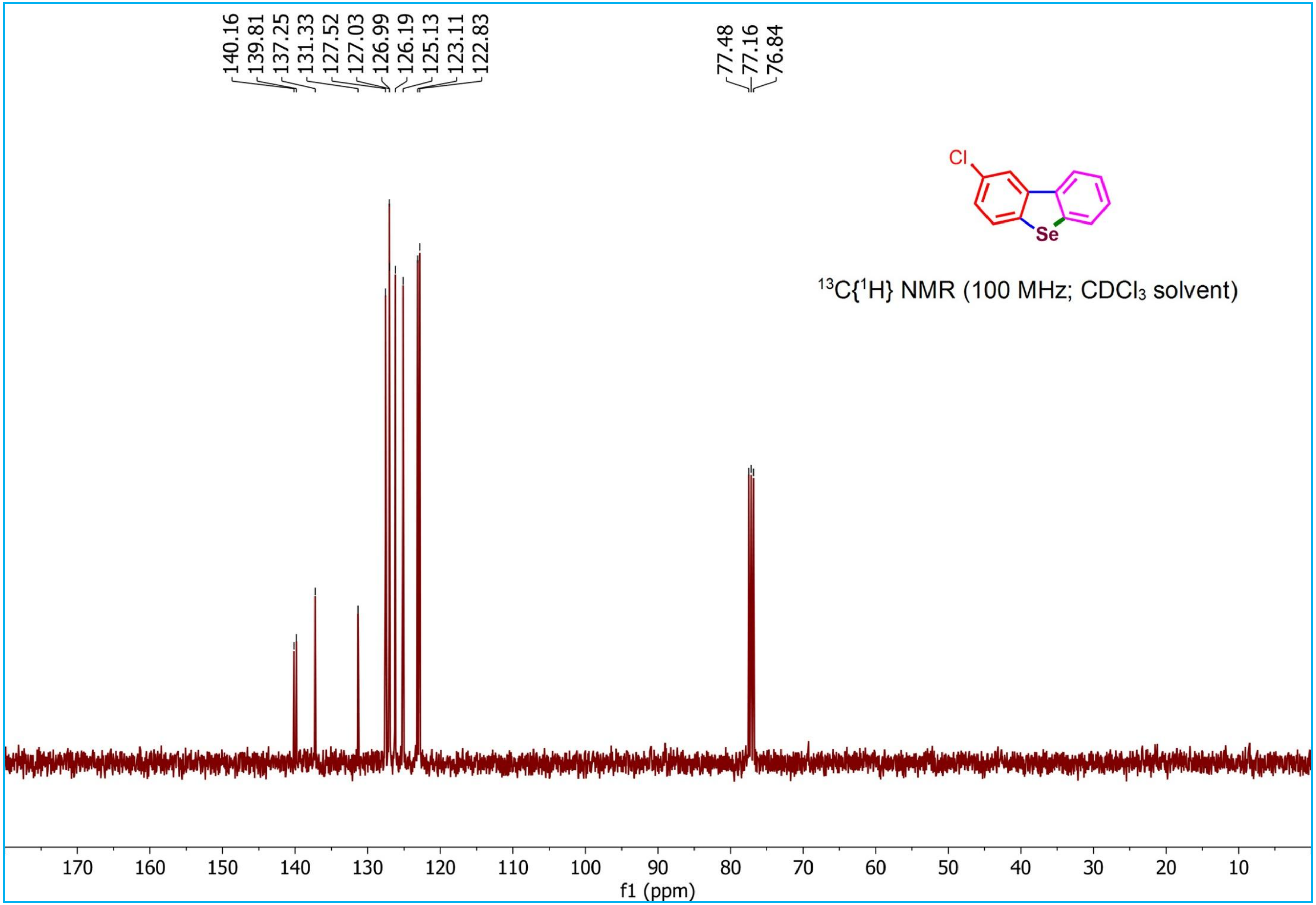

Figure S15. $^{13}C\{^{1}H\}$ NMR spectrum of 2-chlorodibenzo[b,d]selenophene (**2ea**)

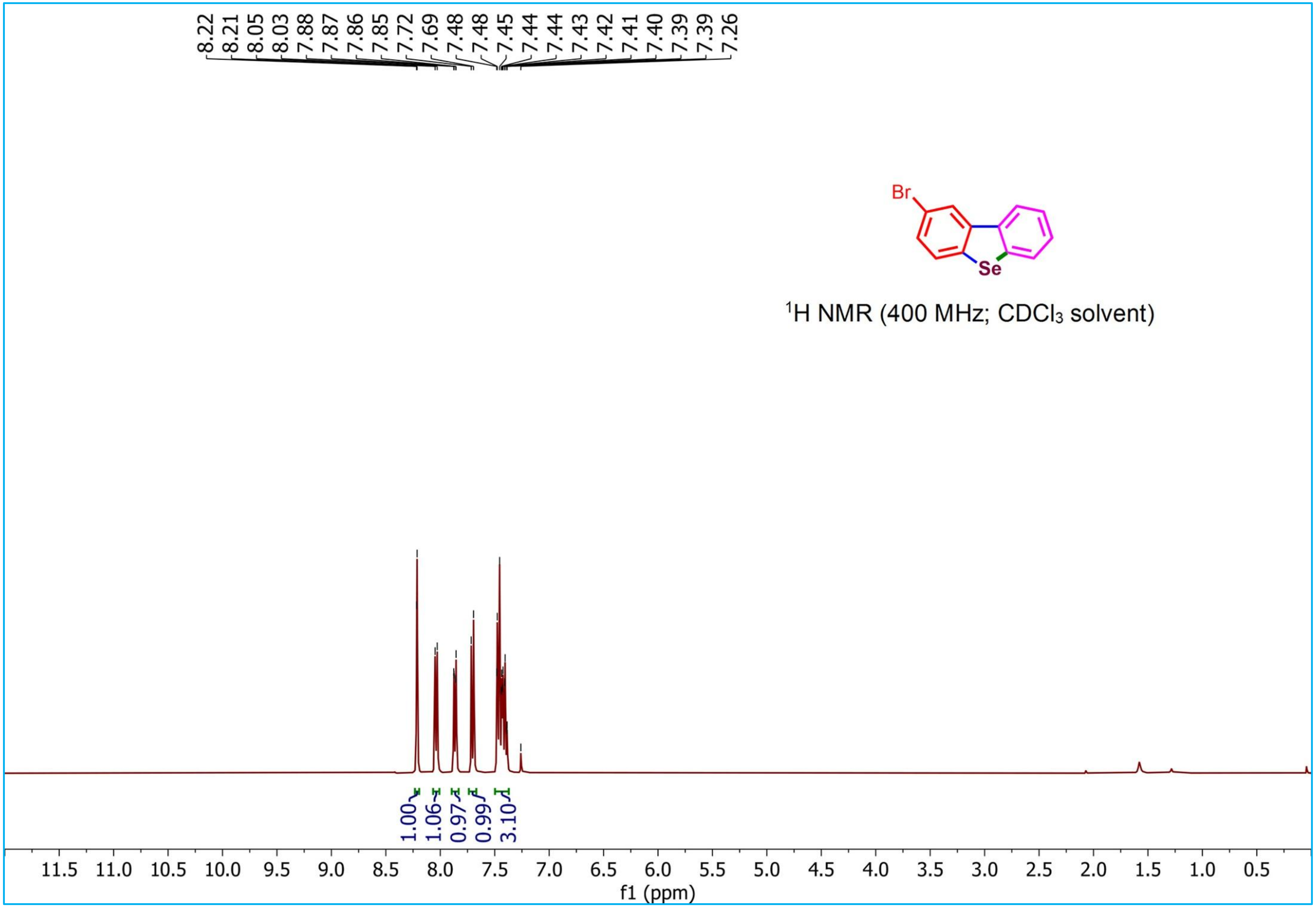

Figure S16. ^{1}H NMR spectrum of 2-bromodibenzo[b,d]selenophene (**2fa**)

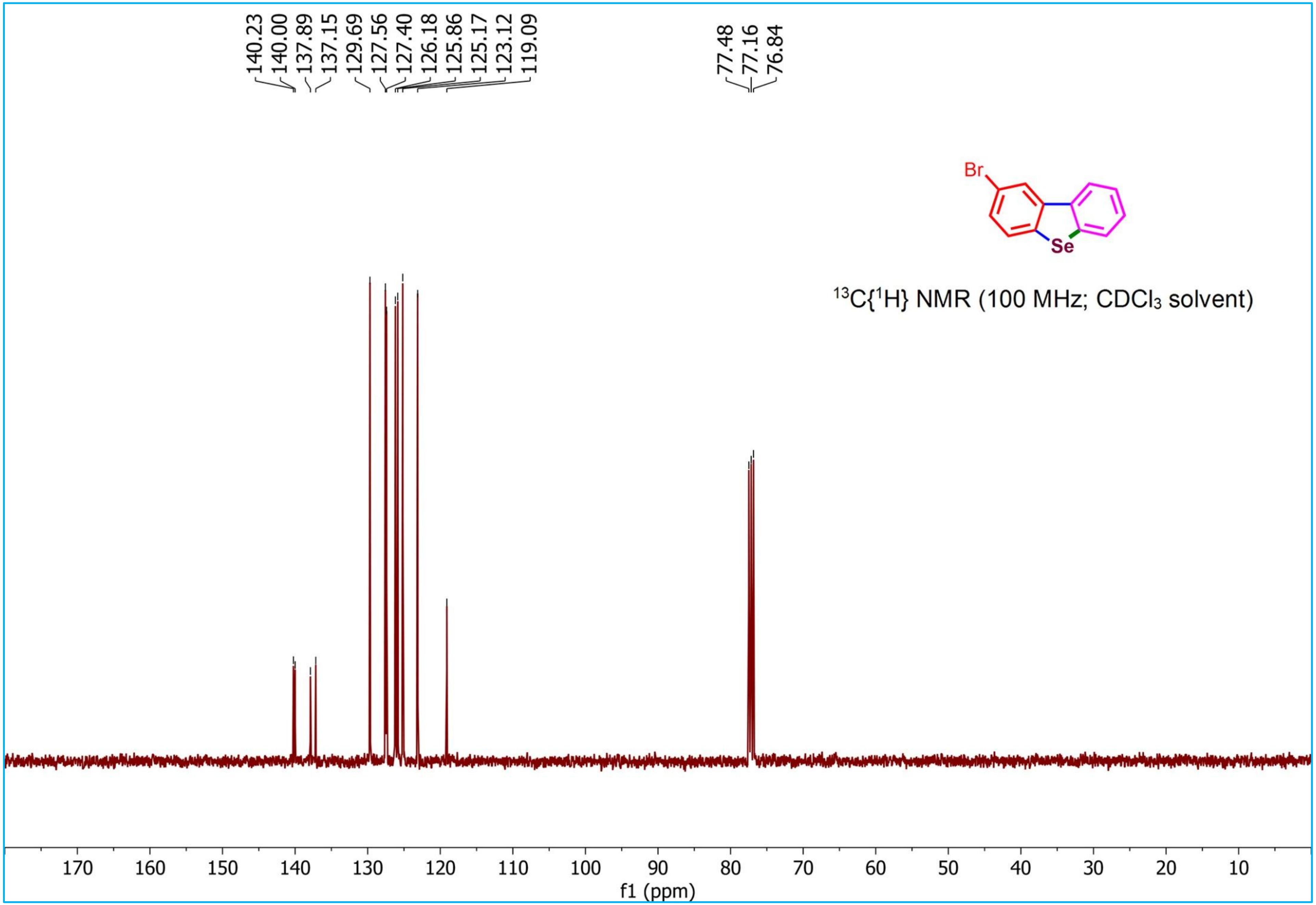

Figure S17. $^{13}C\{^1H\}$ NMR spectrum of 2-bromodibenzo[b,d]selenophene (**2fa**)

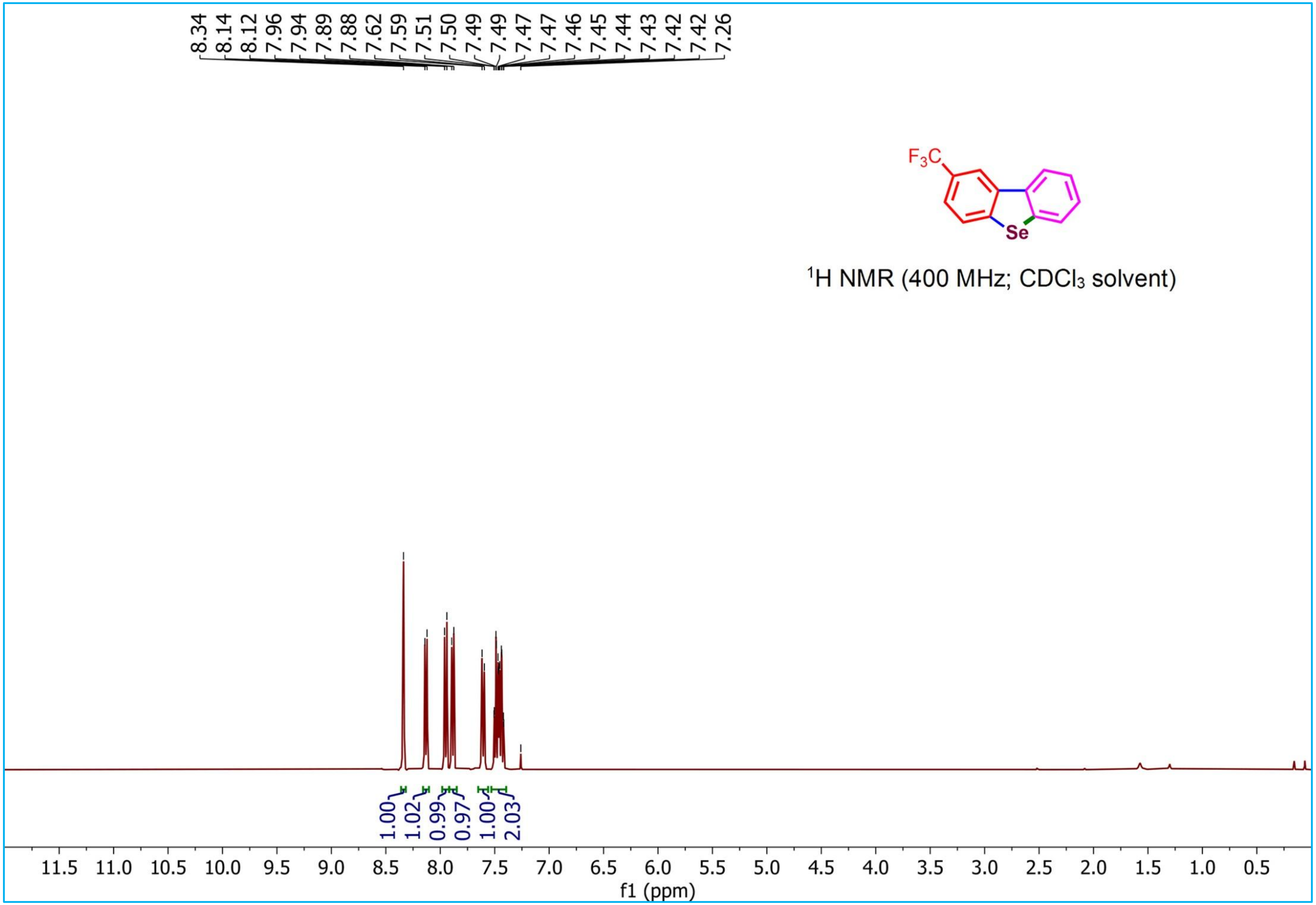

Figure S18. ^{1}H NMR spectrum of 2-(trifluoromethyl)dibenzo[b,d]selenophene (**2ga**)

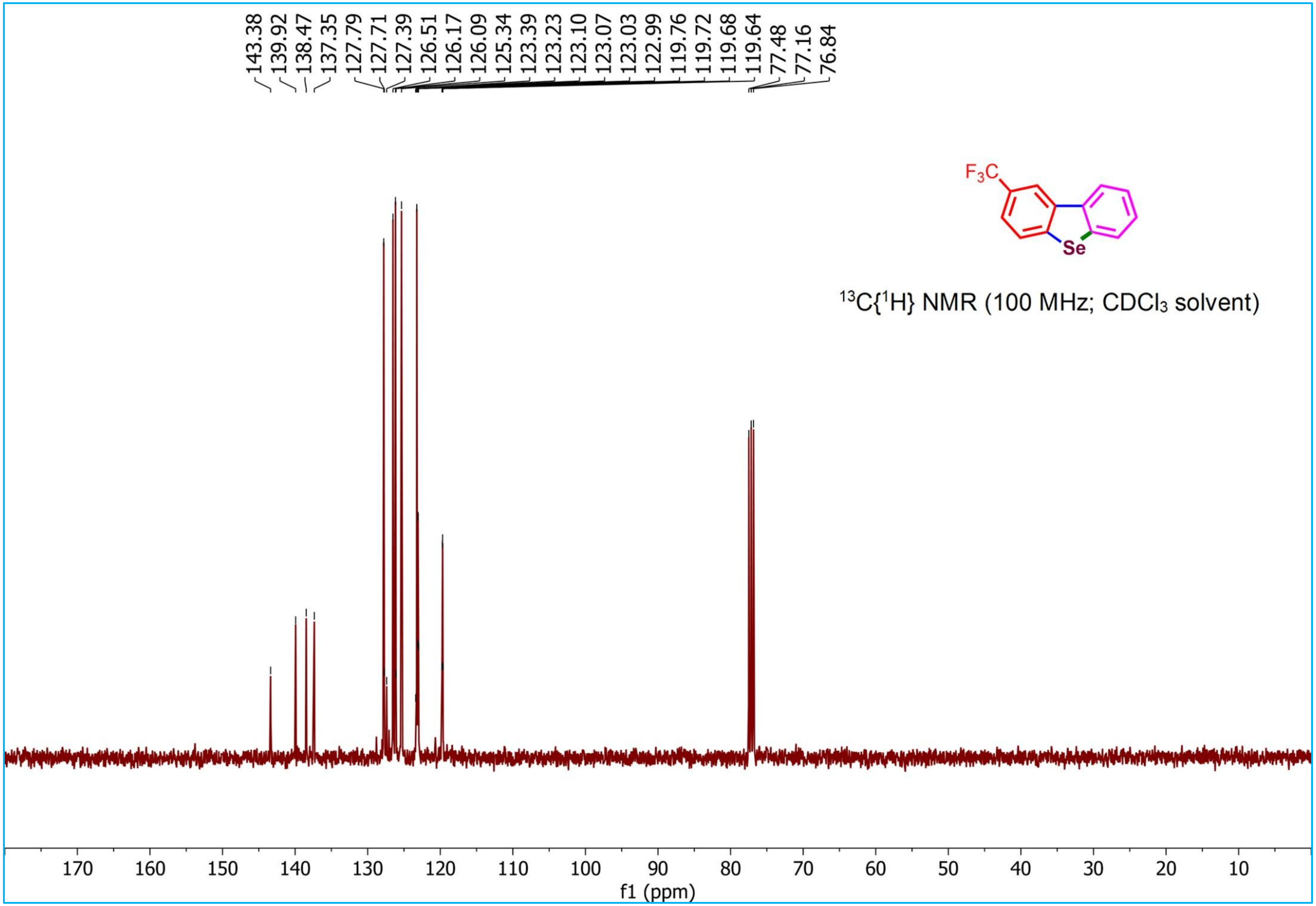

Figure S19. $^{13}C\{^{1}H\}$ NMR spectrum of 2-(trifluoromethyl)dibenzo[b,d]selenophene (**2ga**)

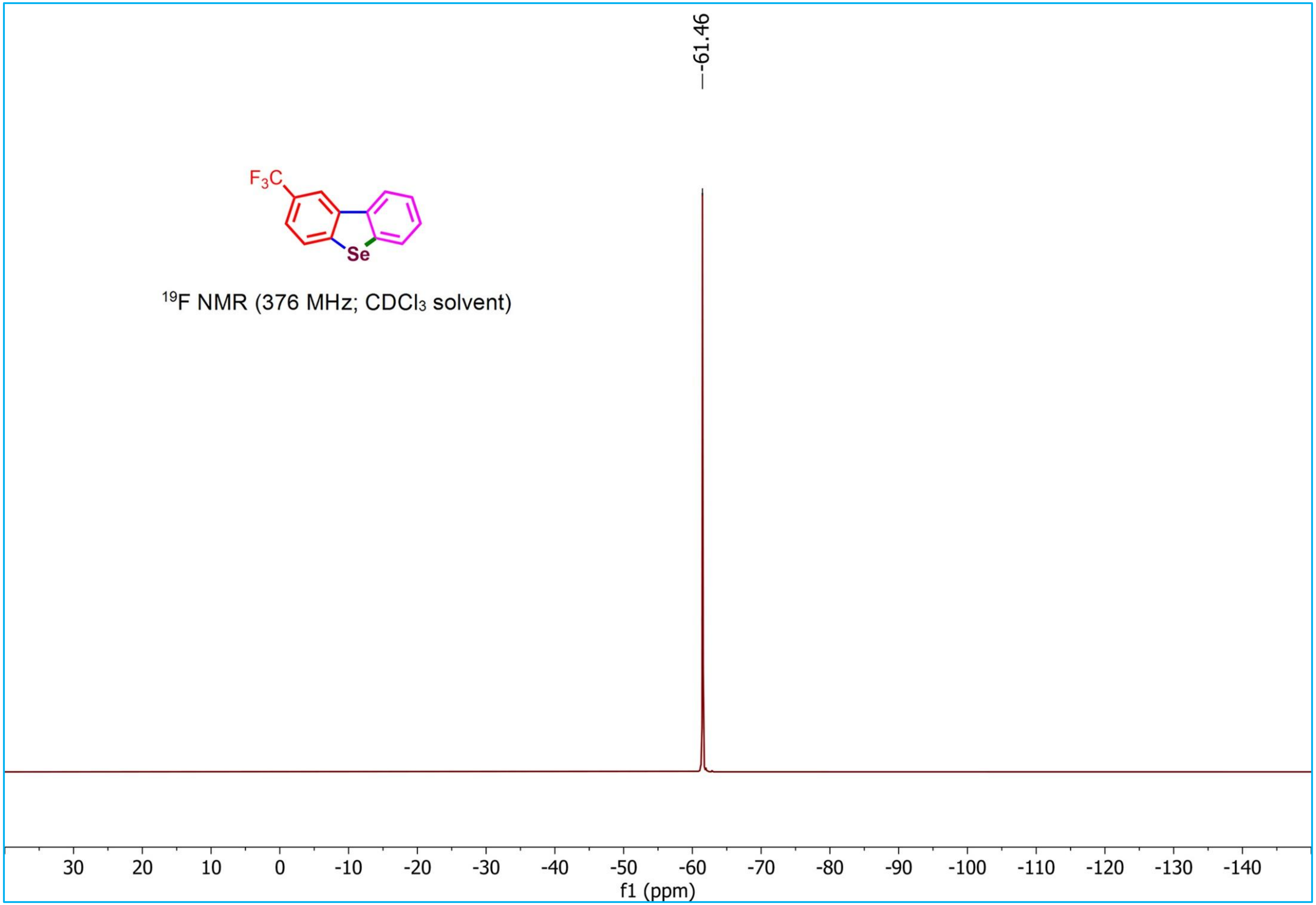

Figure S20. ^{19}F NMR spectrum of 2-(trifluoromethyl)dibenzo[b,d]selenophene (**2ga**)

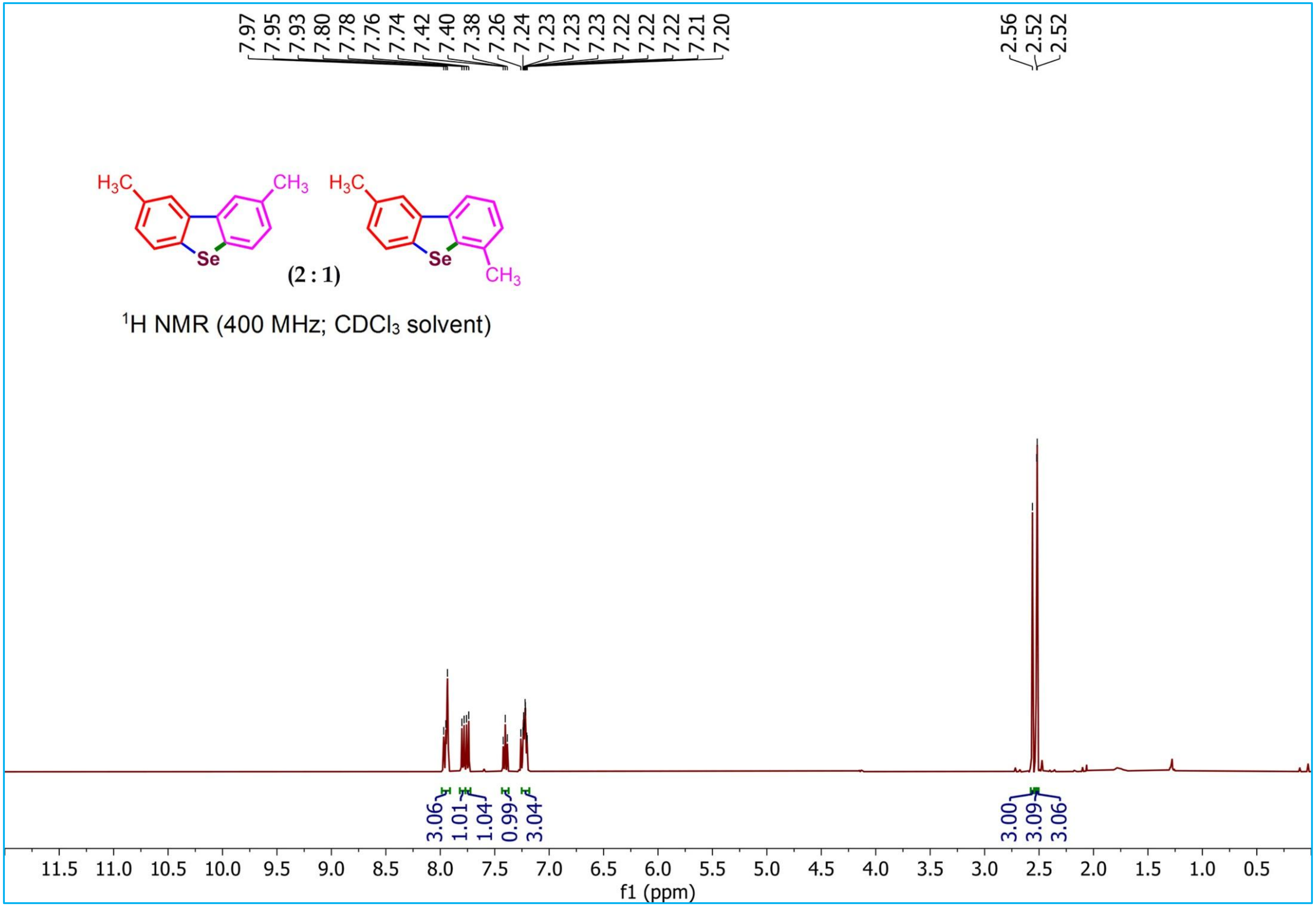

Figure S21. ^{1}H NMR spectrum of 2,8-dimethyldibenzo[b,d]selenophene (**2bb**) and 2,6-dimethyldibenzo[b,d]selenophene (**2bb′**)

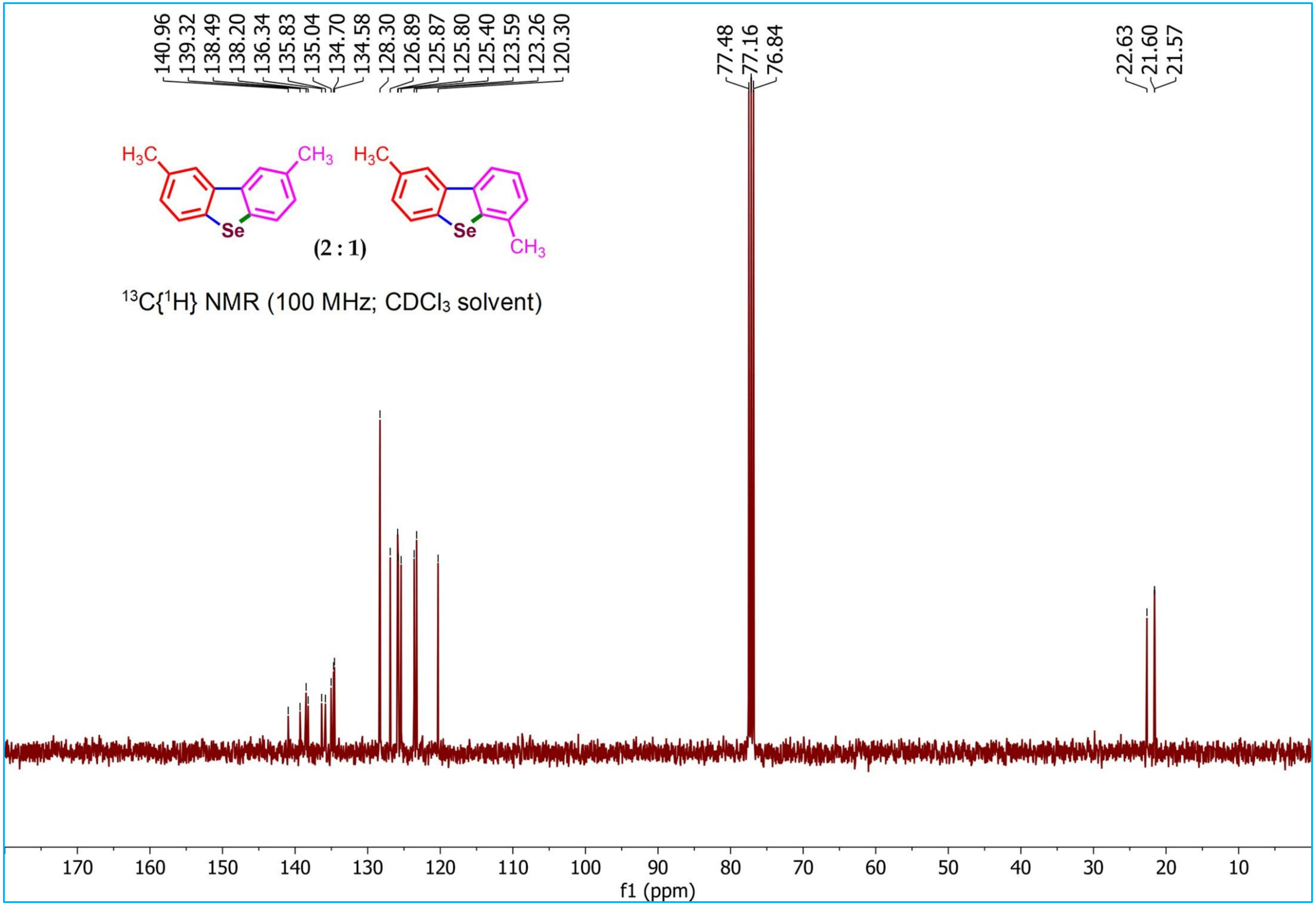

Figure S22. $^{13}C\{^1H\}$ NMR spectrum of 2,8-dimethyldibenzo[b,d]selenophene (**2bb**) and 2,6-dimethyldibenzo[b,d]selenophene (**2bb′**)

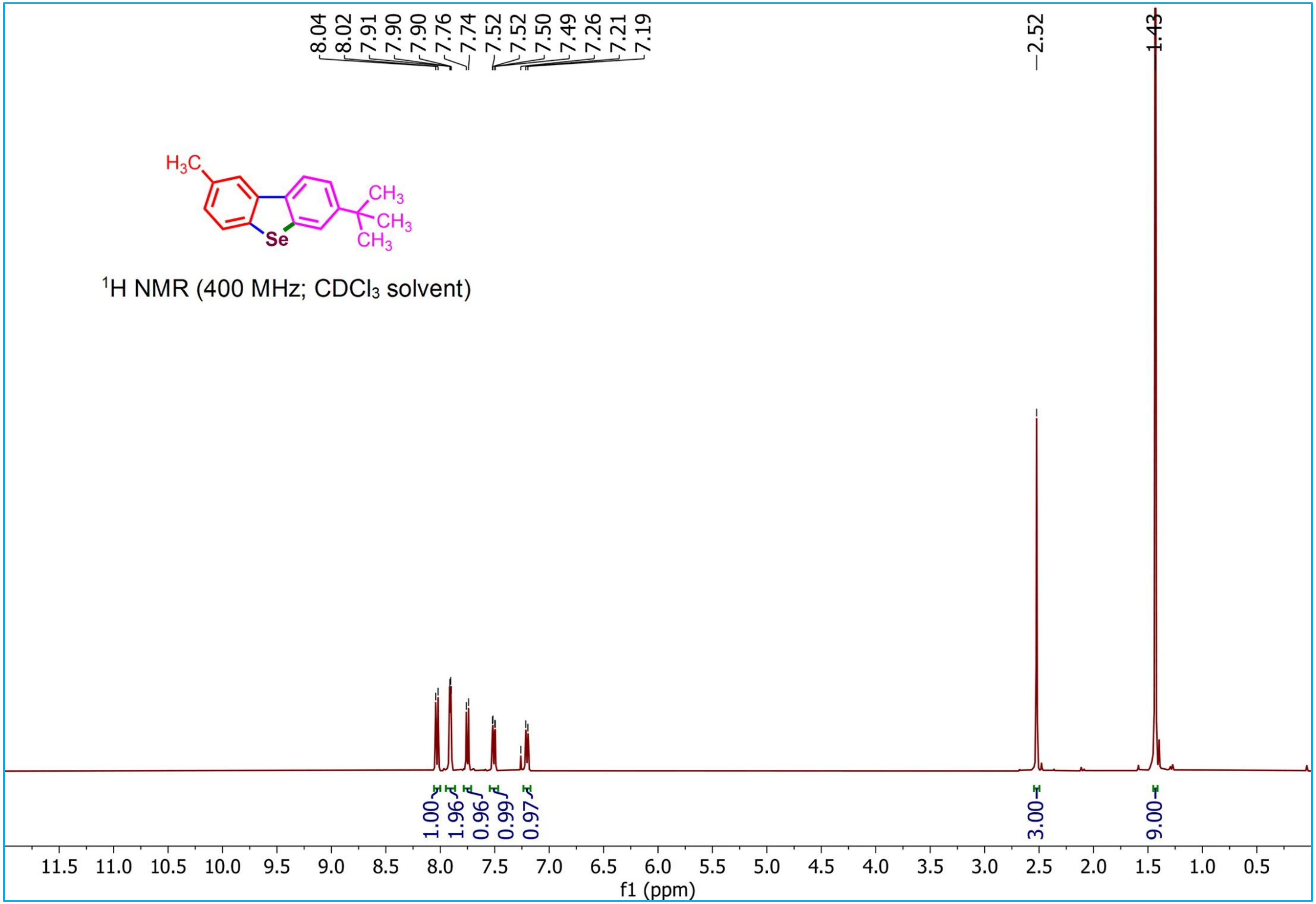

Figure S23. ^1H NMR spectrum of 7-(*tert*-butyl)-2-methyldibenzo[b,d]selenophene (**2bc**)

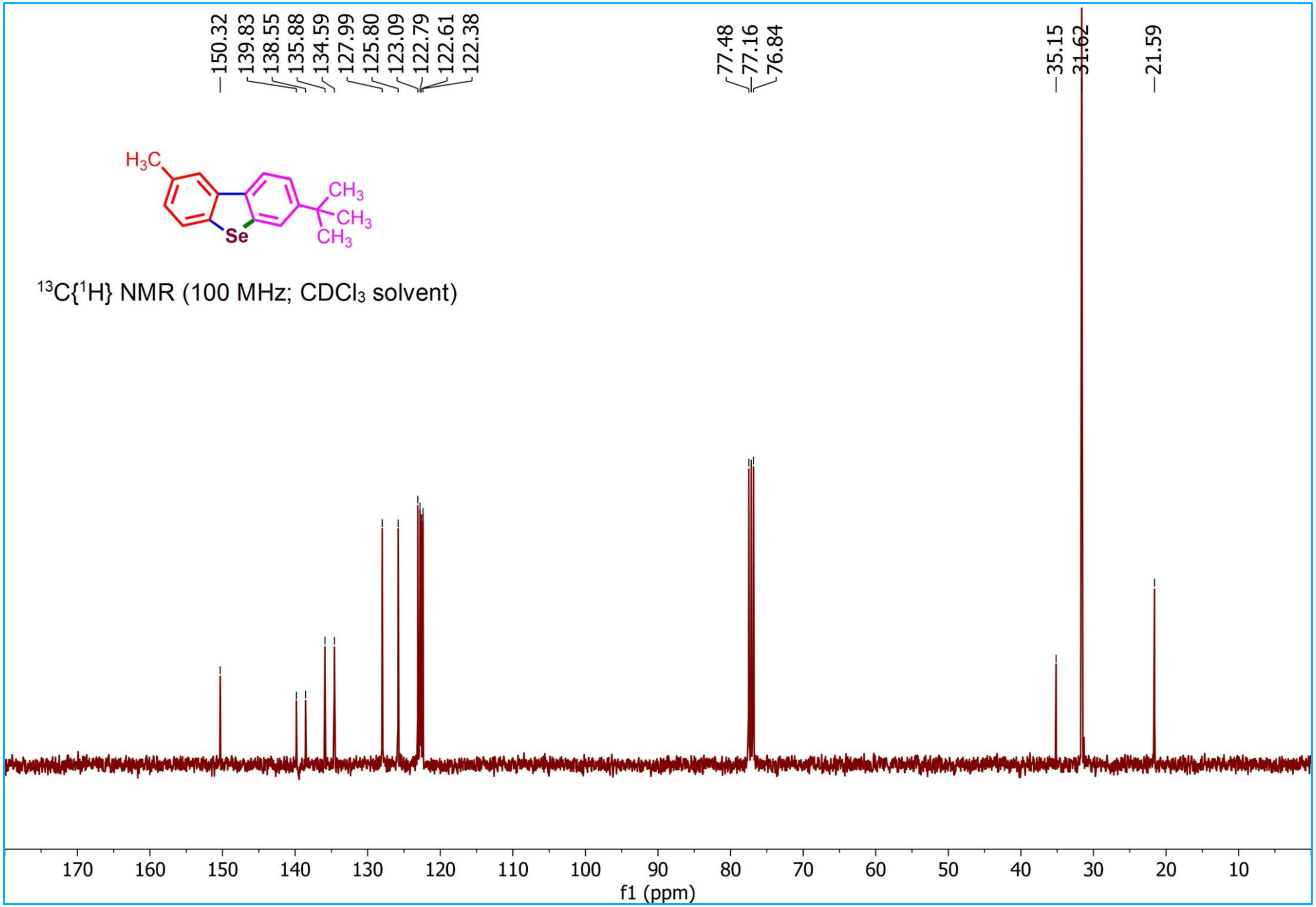

Figure S24. ^{13}C{^{1}H} NMR spectrum of 7-(*tert*-butyl)-2-methyldibenzo[b,d]selenophene (**2bc**)

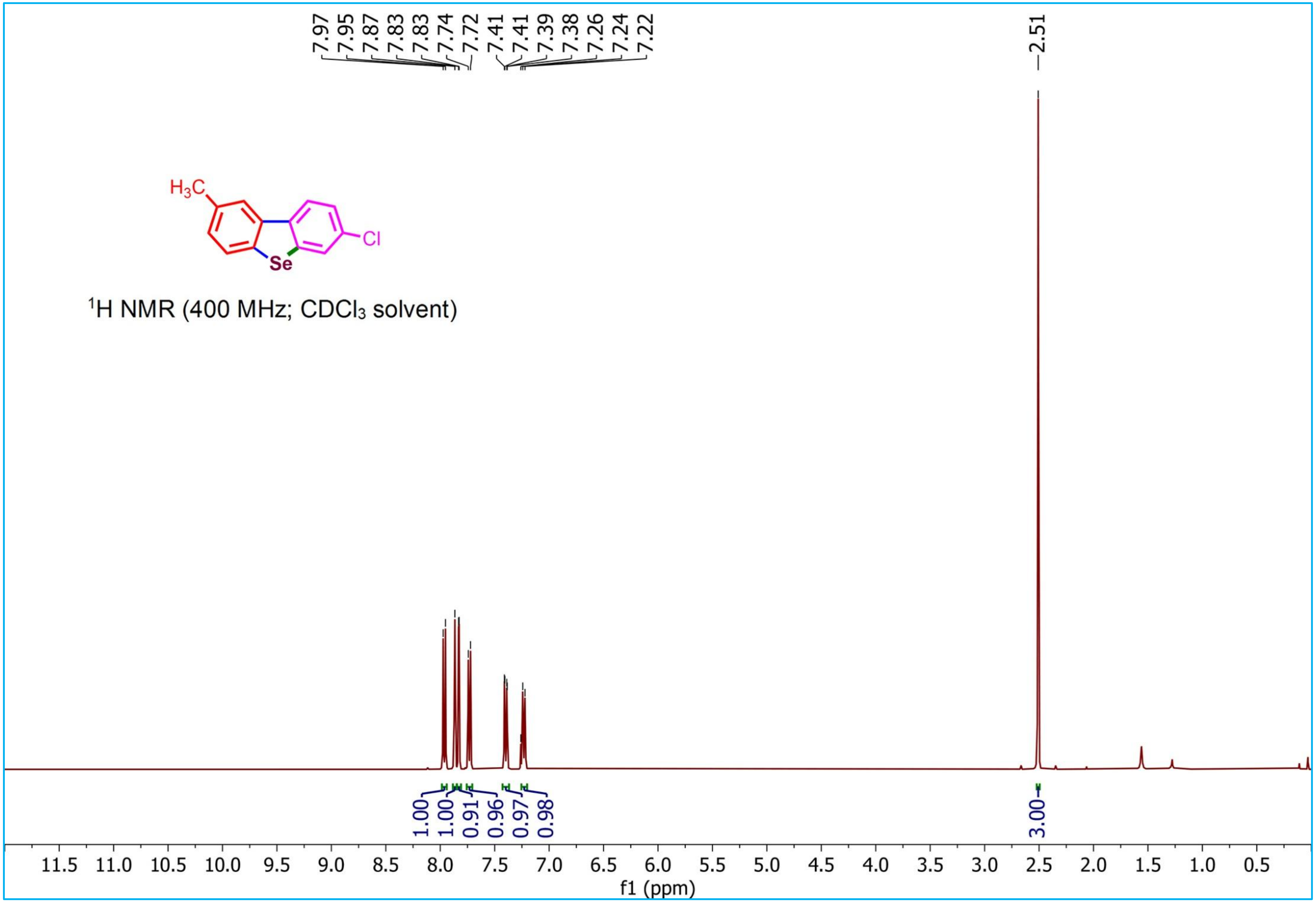

Figure S25. ^{1}H NMR spectrum of 7-chloro-2-methyldibenzo[b,d]selenophene (**2bd**)

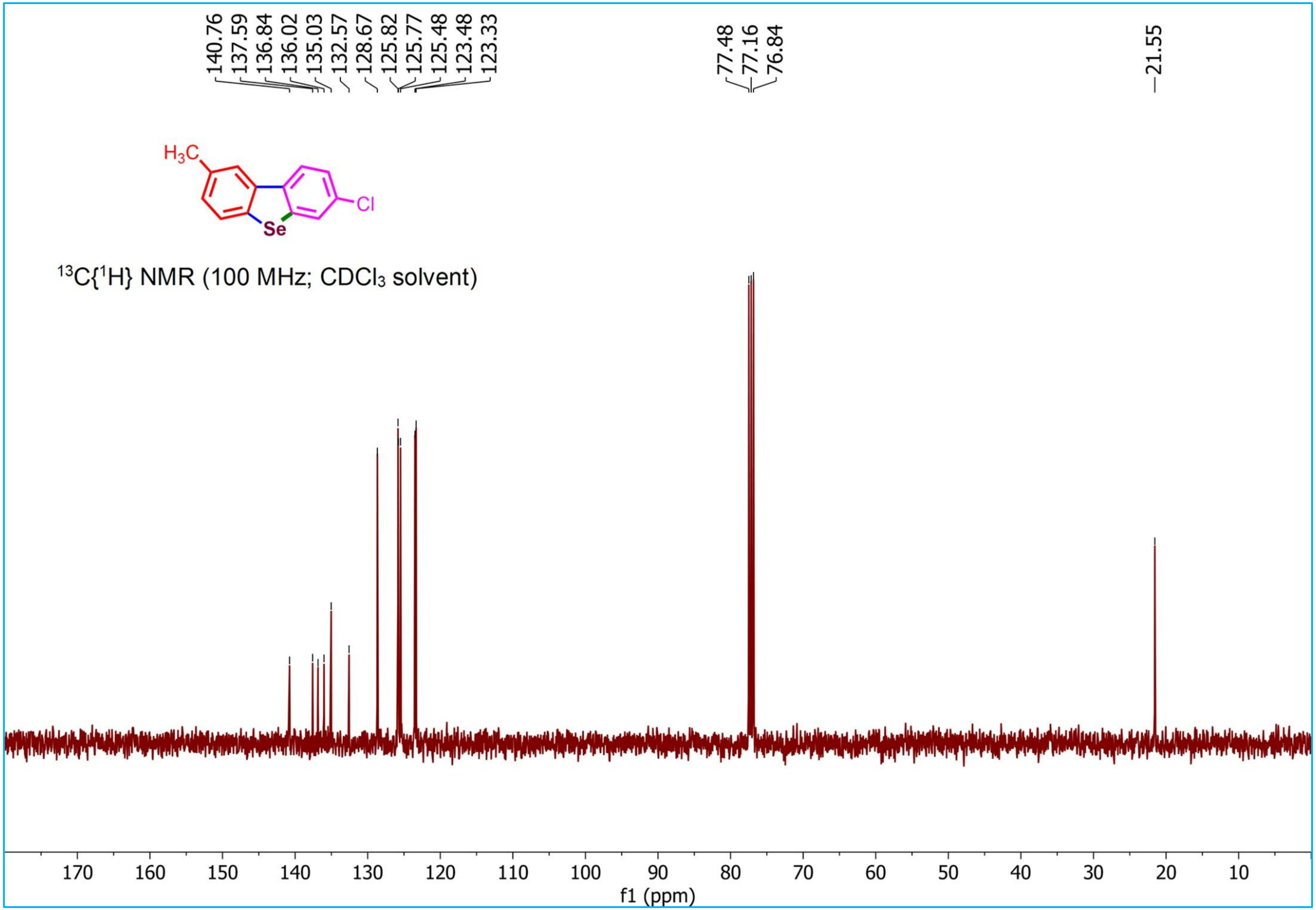

Figure S26. $^{13}C\{^{1}H\}$ NMR spectrum of 7-chloro-2-methyldibenzo[b,d]selenophene (**2bd**)

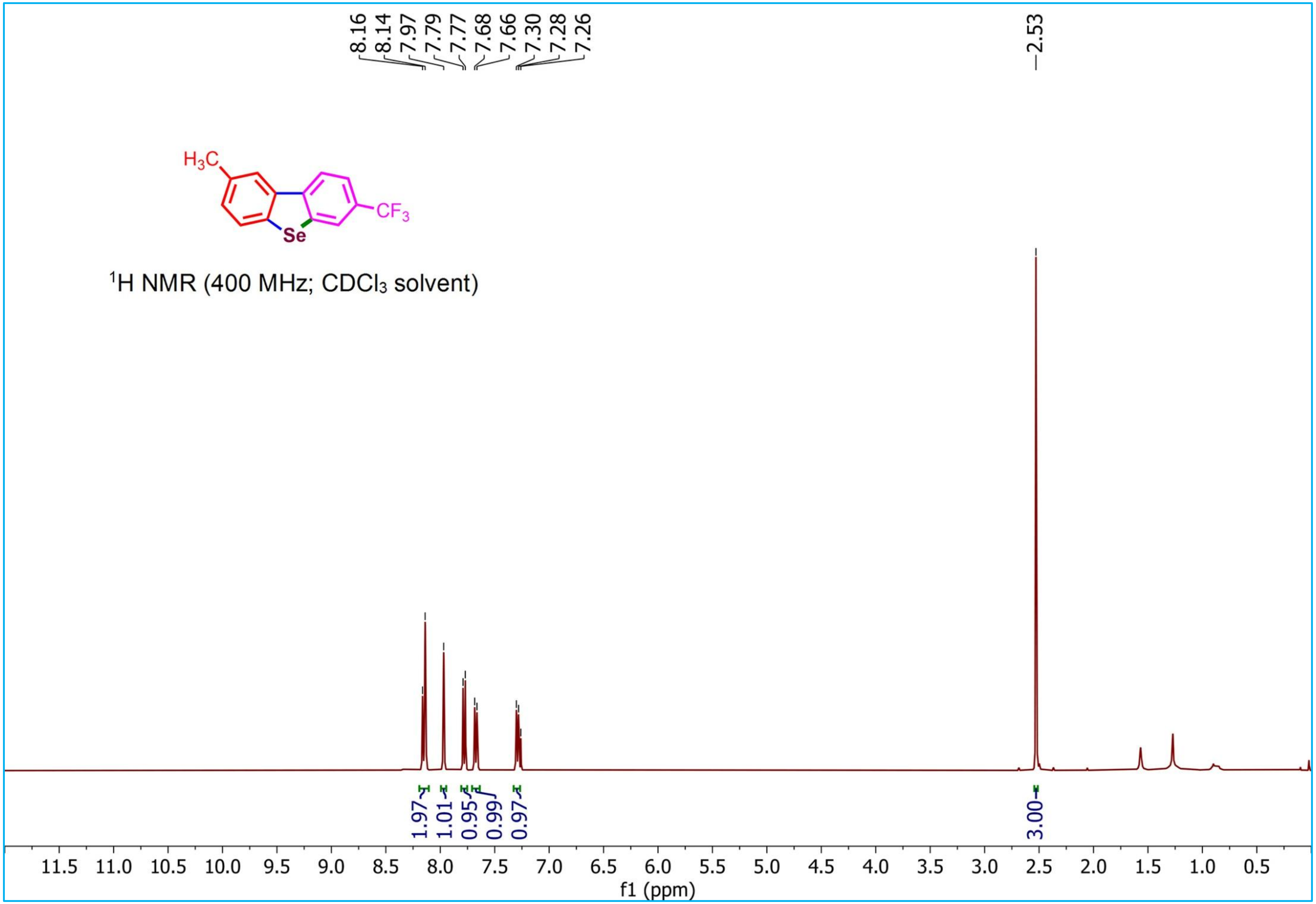

Figure S27. ^{1}H NMR spectrum of 2-methyl-7-(trifluoromethyl)dibenzo[b,d]selenophene (**2be**)

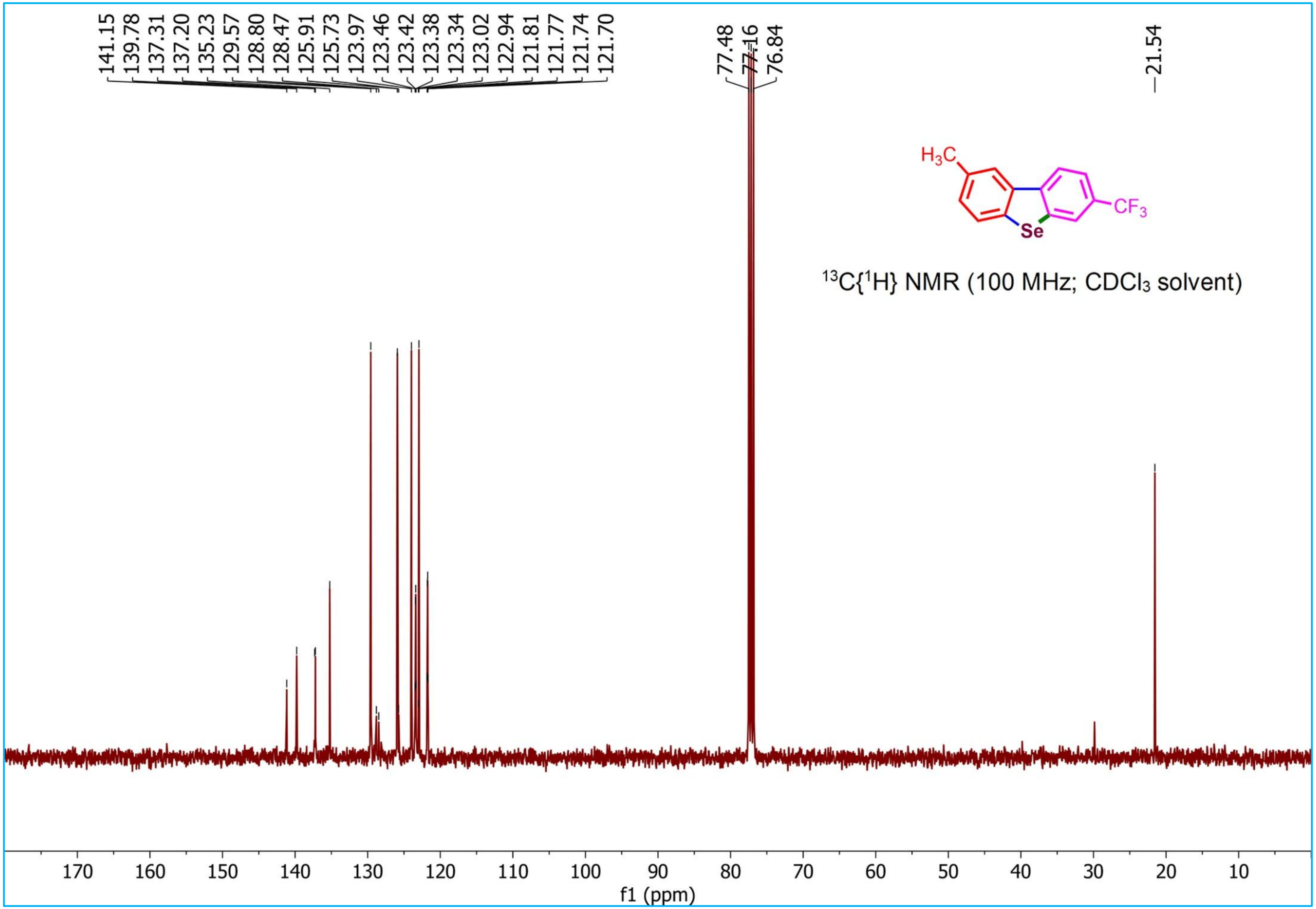

Figure S28. $^{13}C\{^{1}H\}$ NMR spectrum of 2-methyl-7-(trifluoromethyl)dibenzo[b,d]selenophene (**2be**)

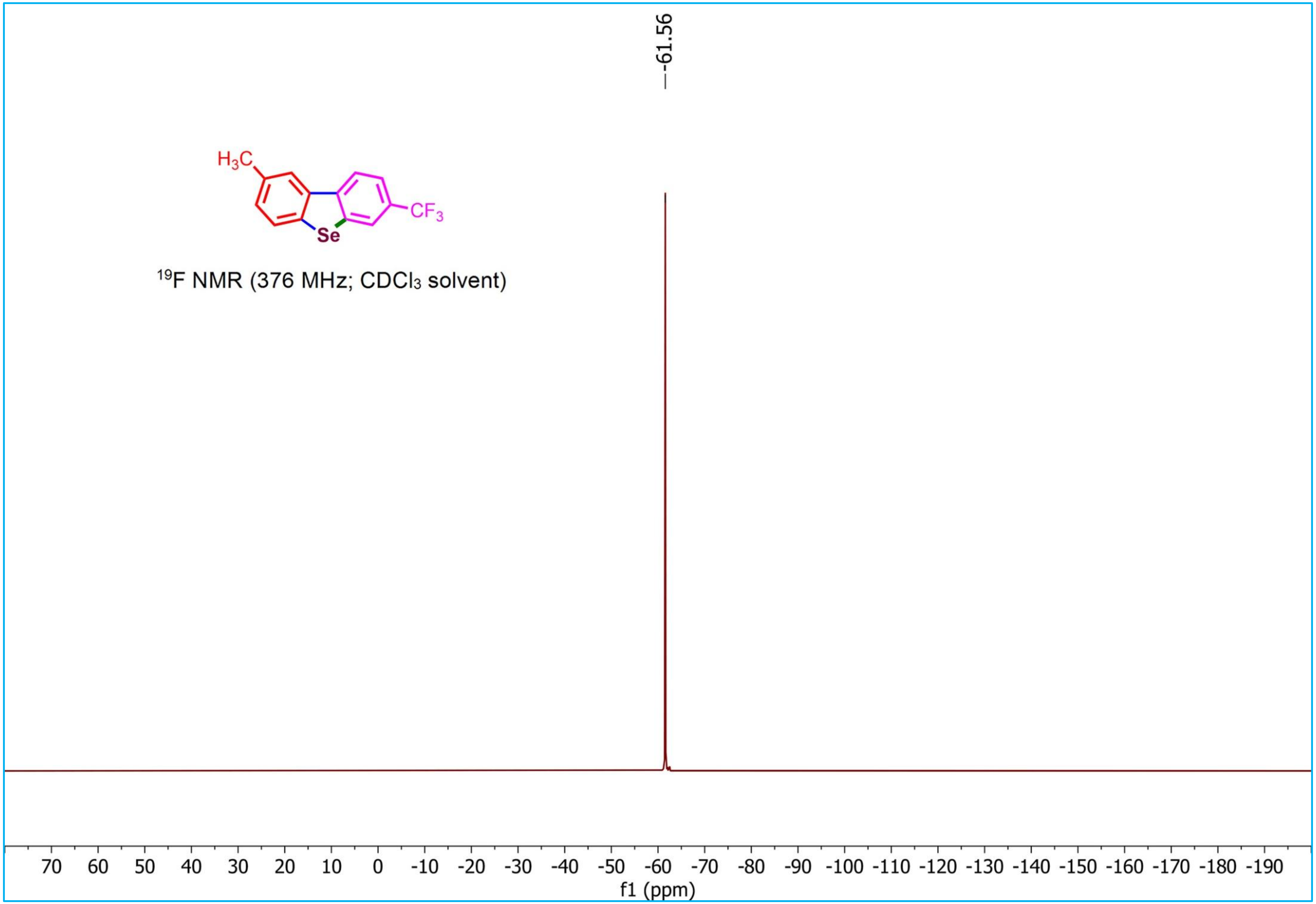

Figure S29. ^{19}F NMR spectrum of 2-methyl-7-(trifluoromethyl)dibenzo[b,d]selenophene (**2be**)

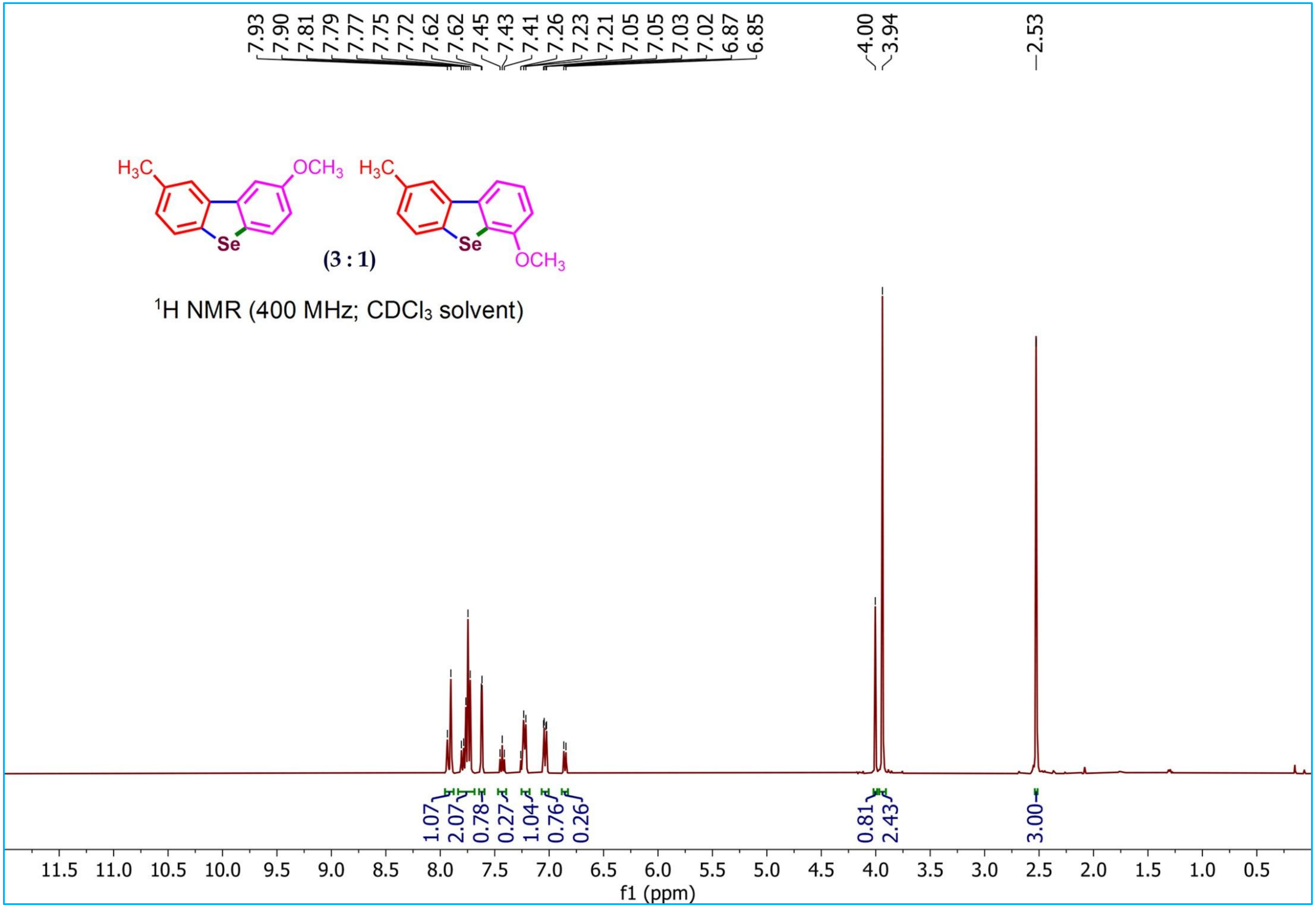

Figure S30. ^{1}H NMR spectrum of 2-methoxy-8-methyldibenzo[b,d]selenophene (**2bf**) and 6-methoxy-2-methyldibenzo[b,d]selenophene (**2bf′**)

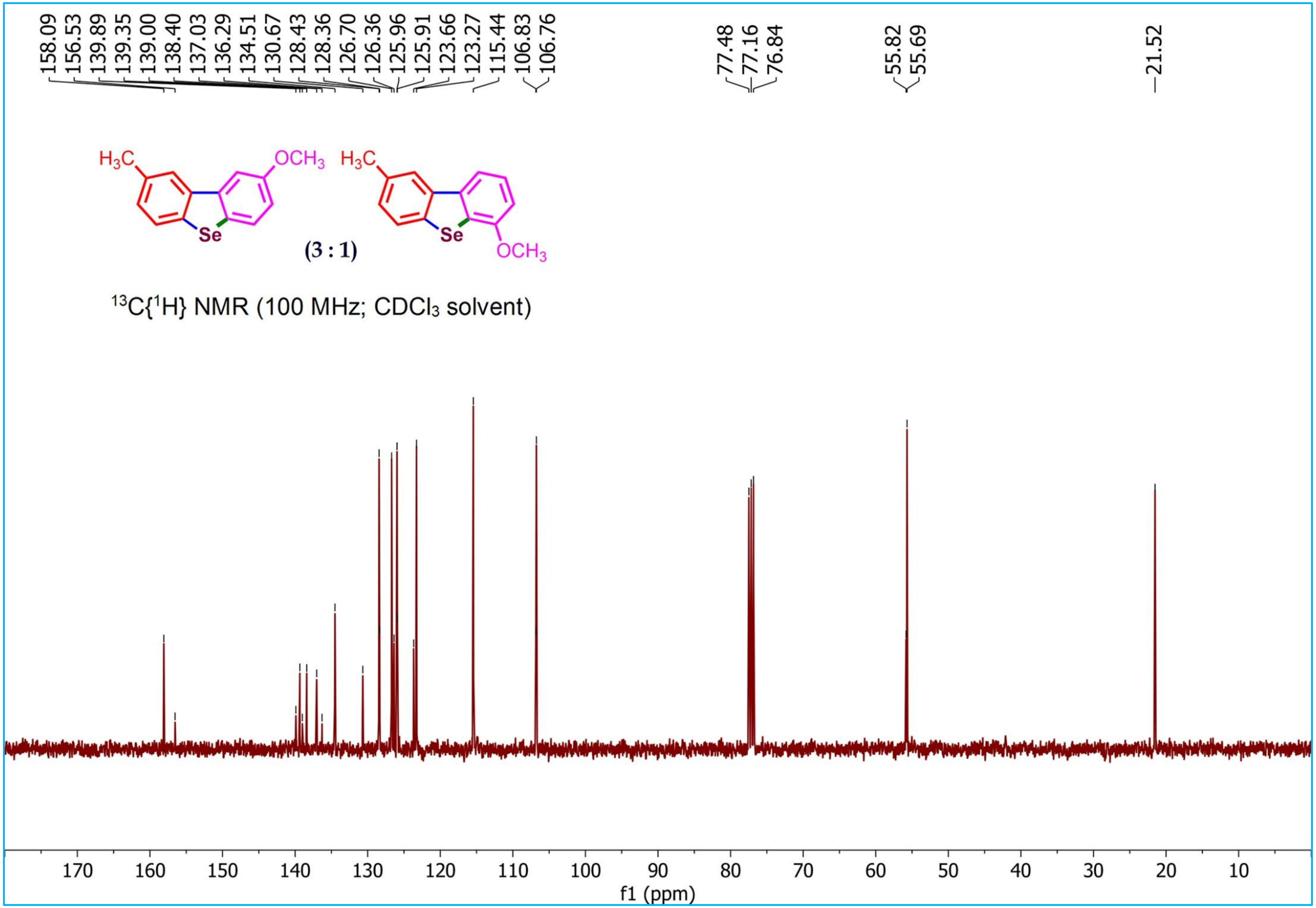

Figure S31. $^{13}C\{^{1}H\}$ NMR spectrum of 2-methoxy-8-methyldibenzo[b,d]selenophene (**2bf**) and 6-methoxy-2-methyldibenzo[b,d]selenophene (**2bf′**)

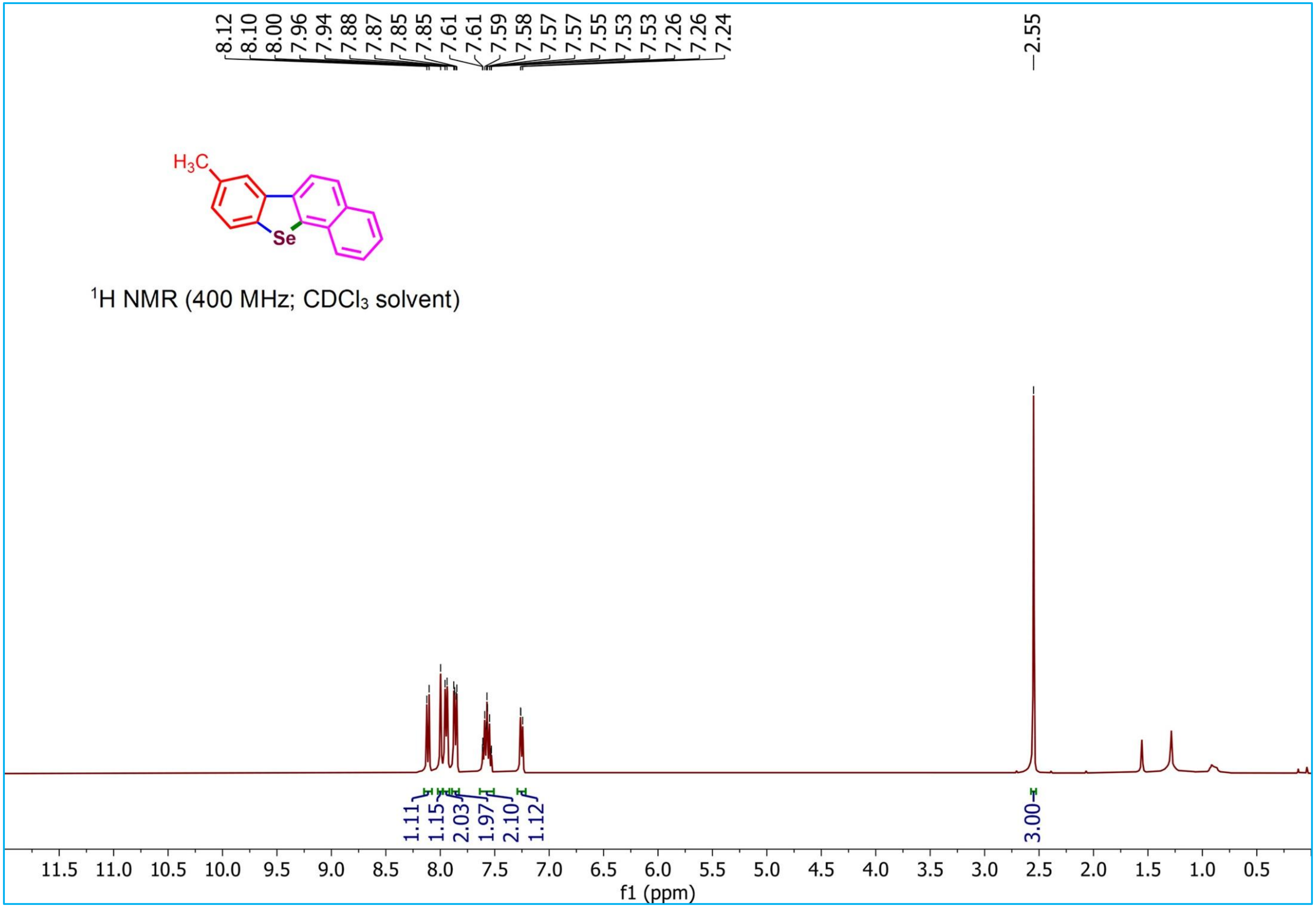

Figure S32. ^1H NMR spectrum of 8-methylbenzo[b]naphtho[2,1-d]selenophene (**2bg**)

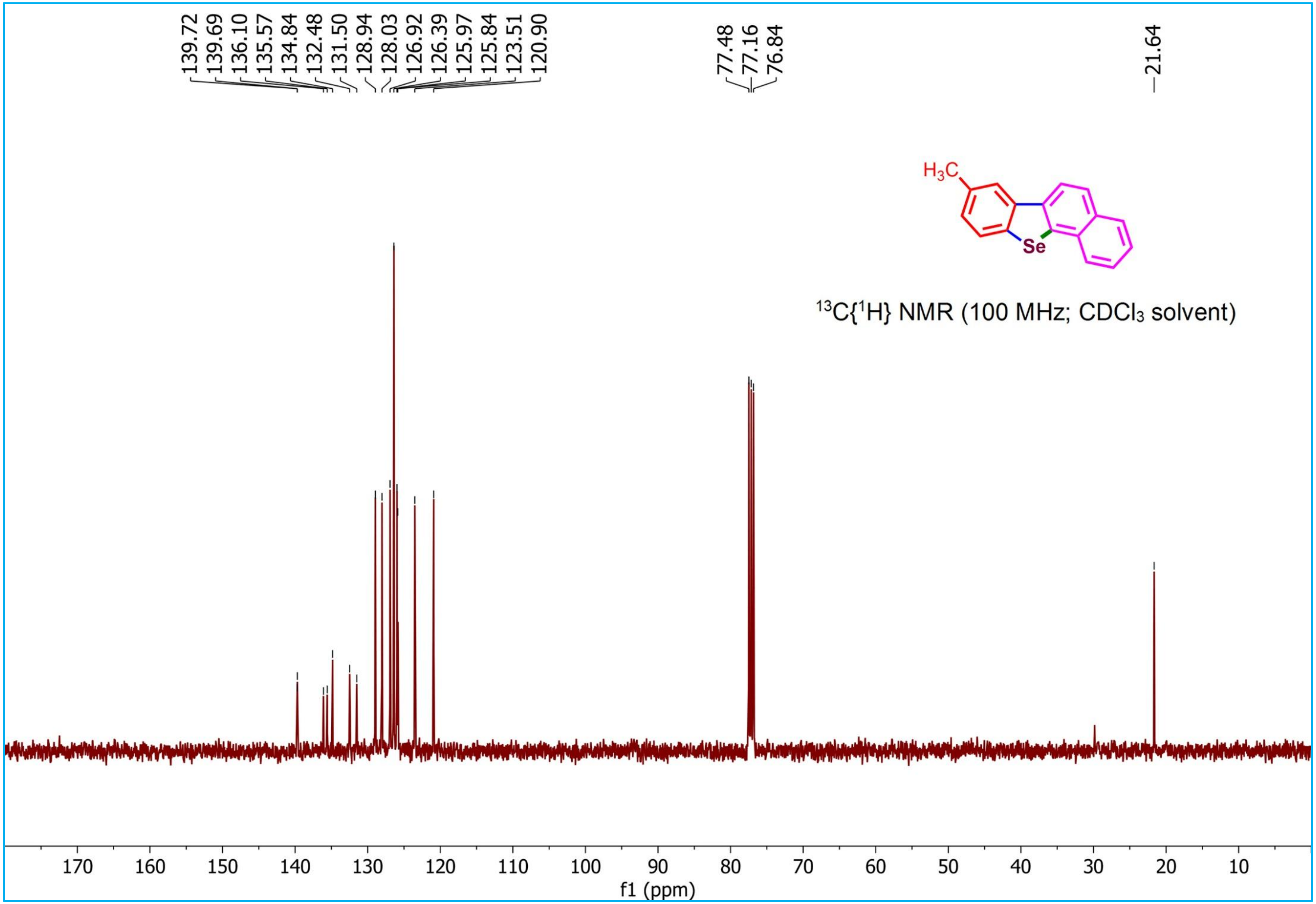

Figure S33. $^{13}C\{^{1}H\}$ NMR spectrum of 8-methylbenzo[b]naphtho[2,1-d]selenophene (**2bg**)

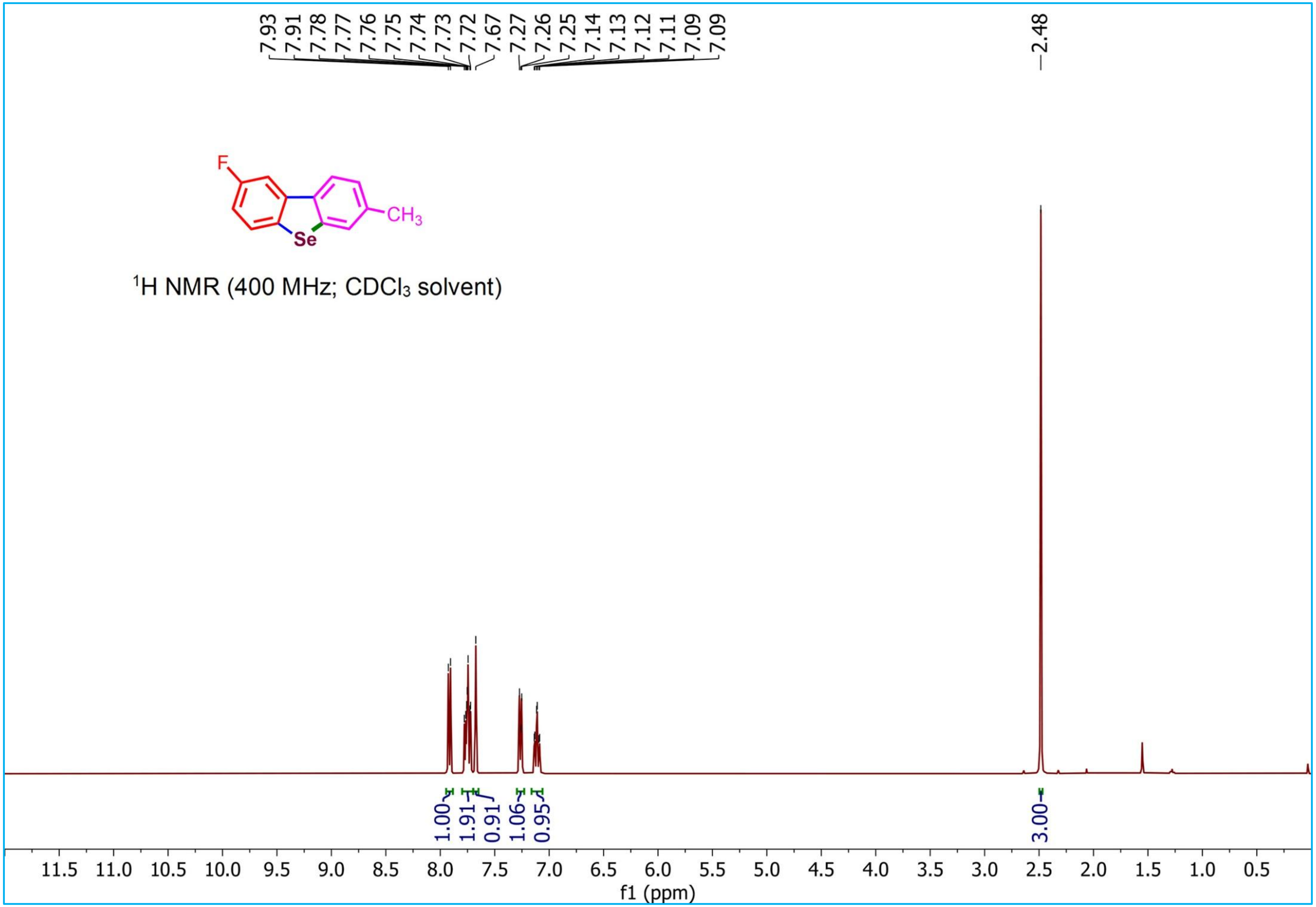

Figure S34. ^{1}H NMR spectrum of 2-fluoro-7-methyldibenzo[b,d]selenophene (**2db**)

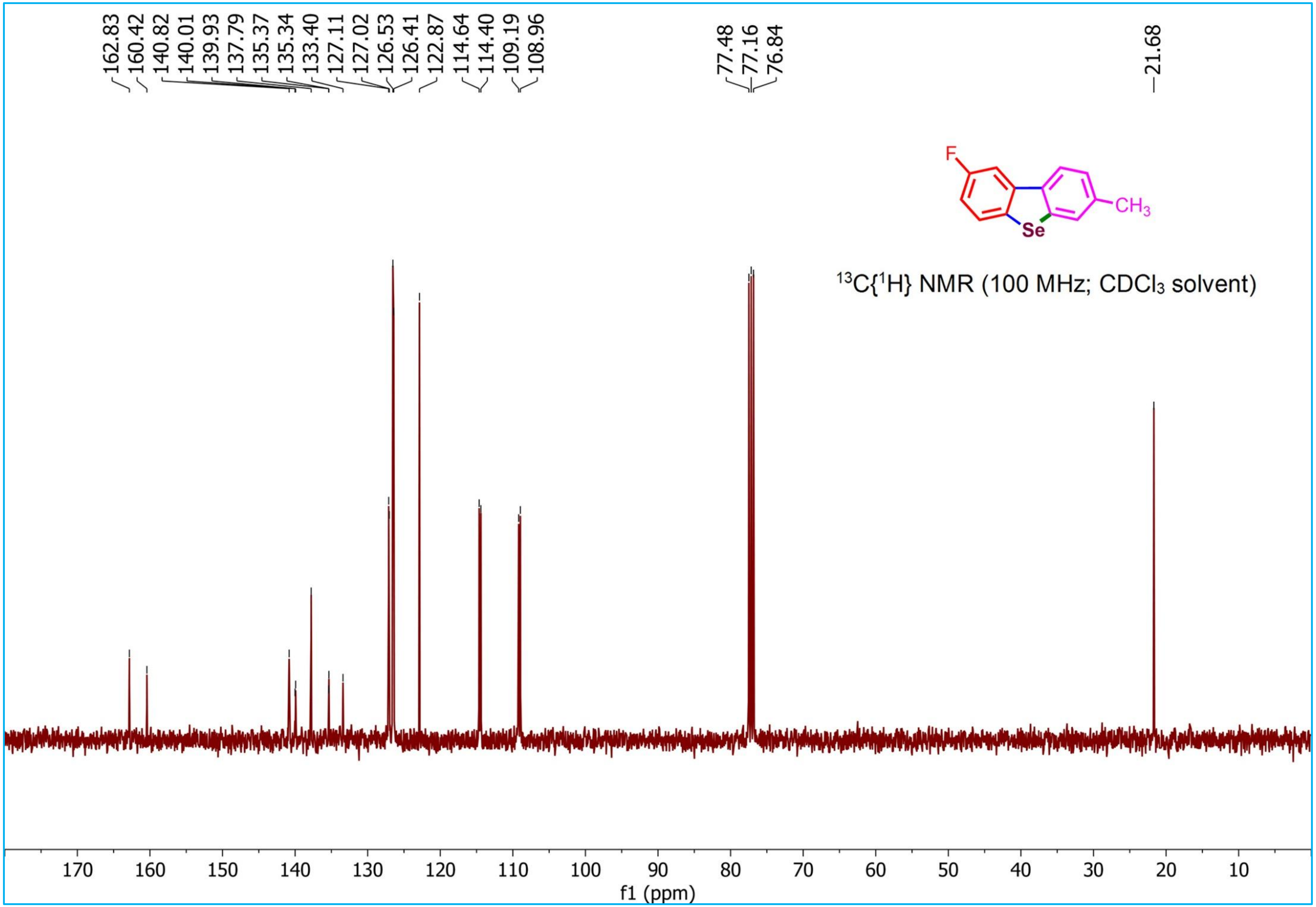

Figure S35. $^{13}C\{^{1}H\}$ NMR spectrum of 2-fluoro-7-methyldibenzo[b,d]selenophene (**2db**)

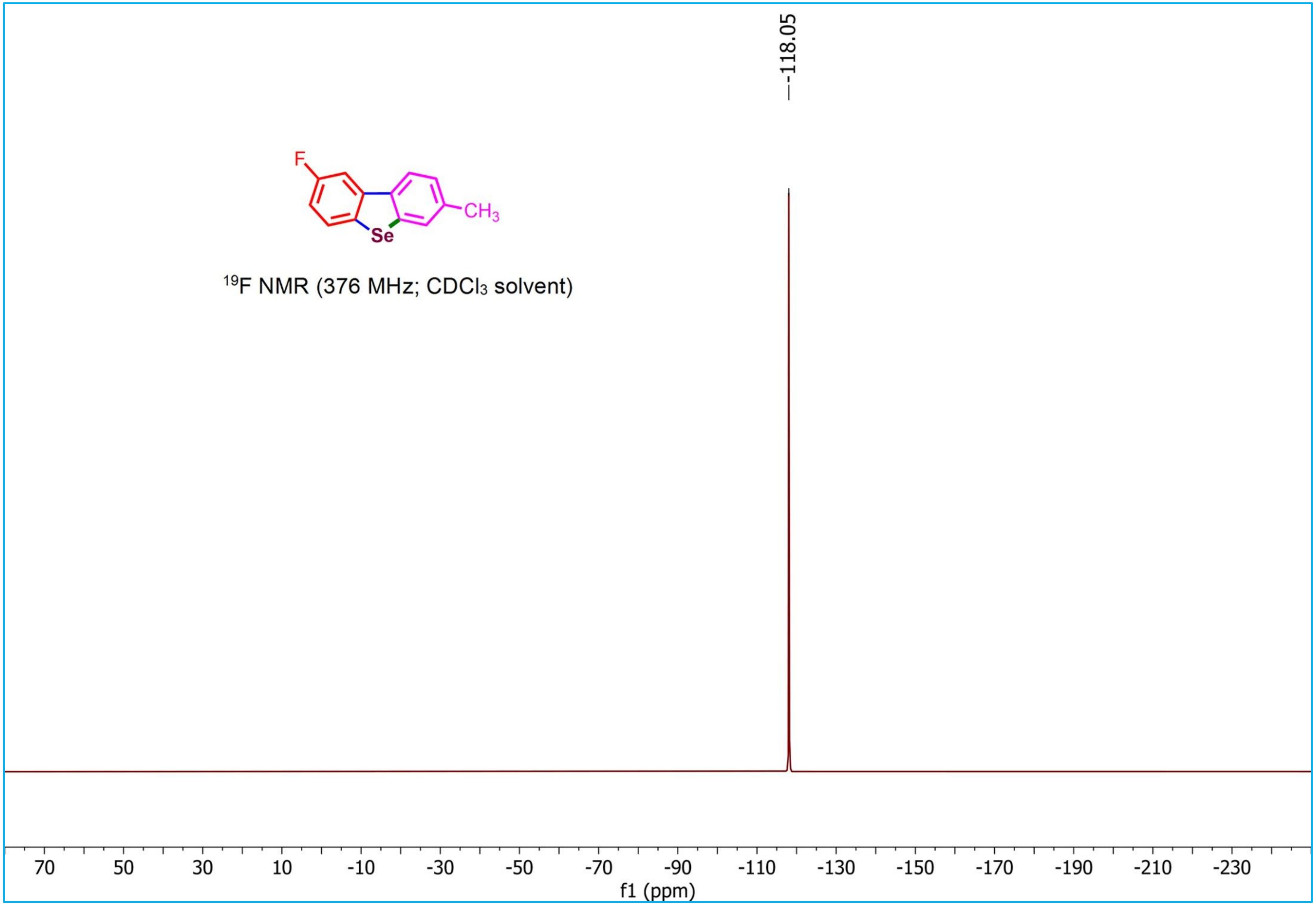

Figure S36. ^{19}F NMR spectrum of 2-fluoro-7-methyldibenzo[b,d]selenophene (**2db**)

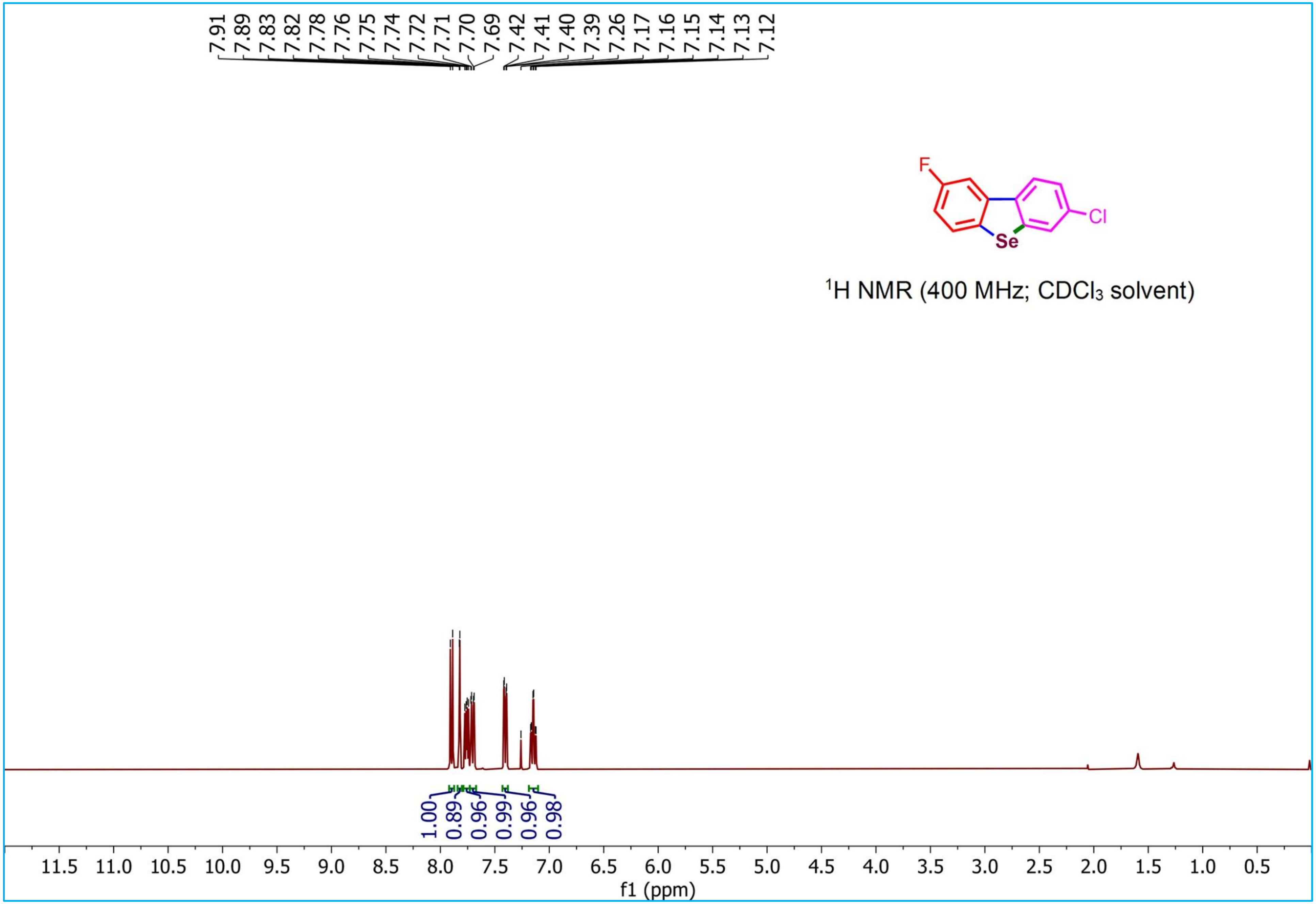

Figure S37. ^{1}H NMR spectrum of 7-chloro-2-fluorodibenzo[b,d]selenophene (**2dd**)

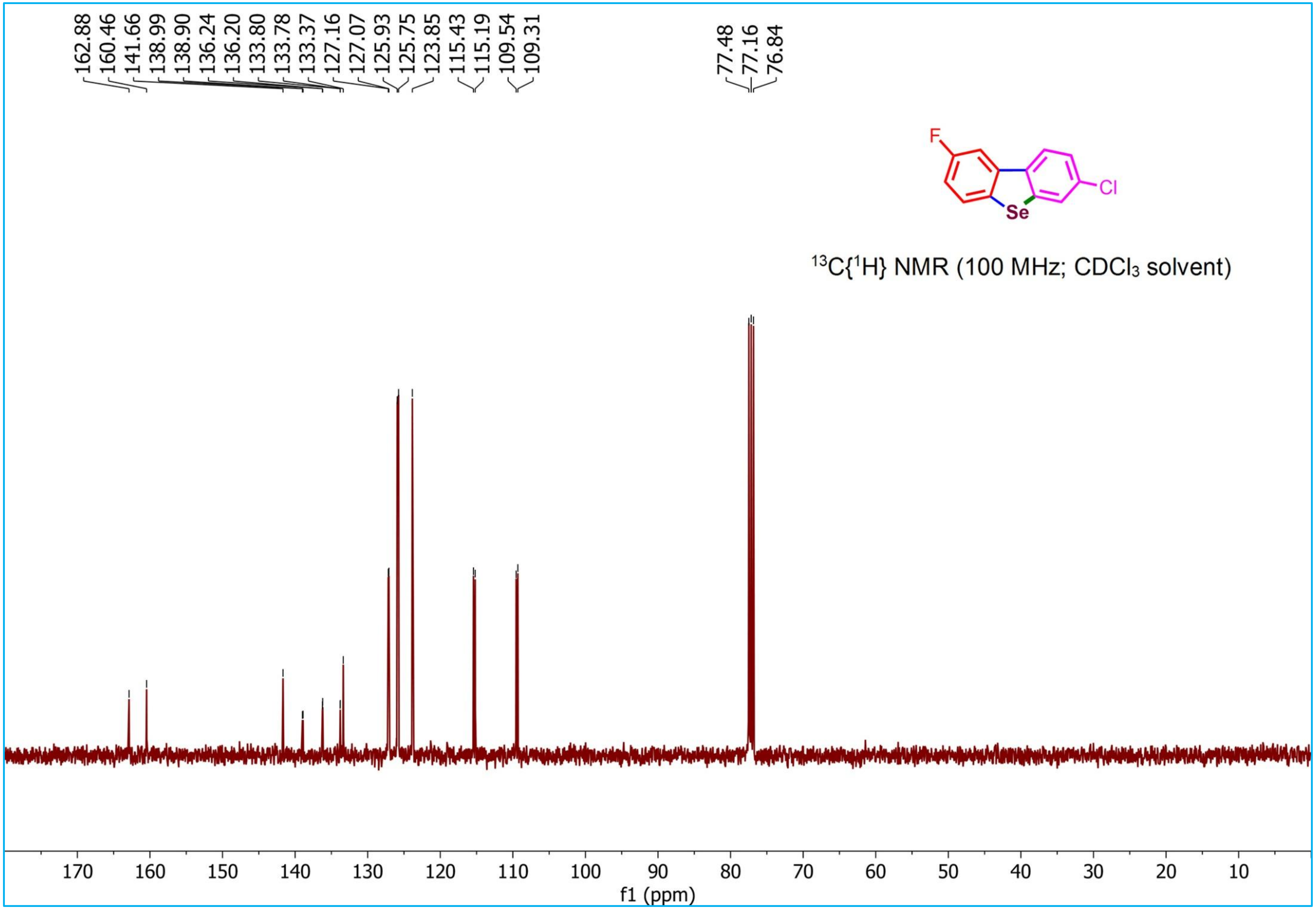

Figure S38. $^{13}C\{^{1}H\}$ NMR spectrum of 7-chloro-2-fluorodibenzo[b,d]selenophene (**2dd**)

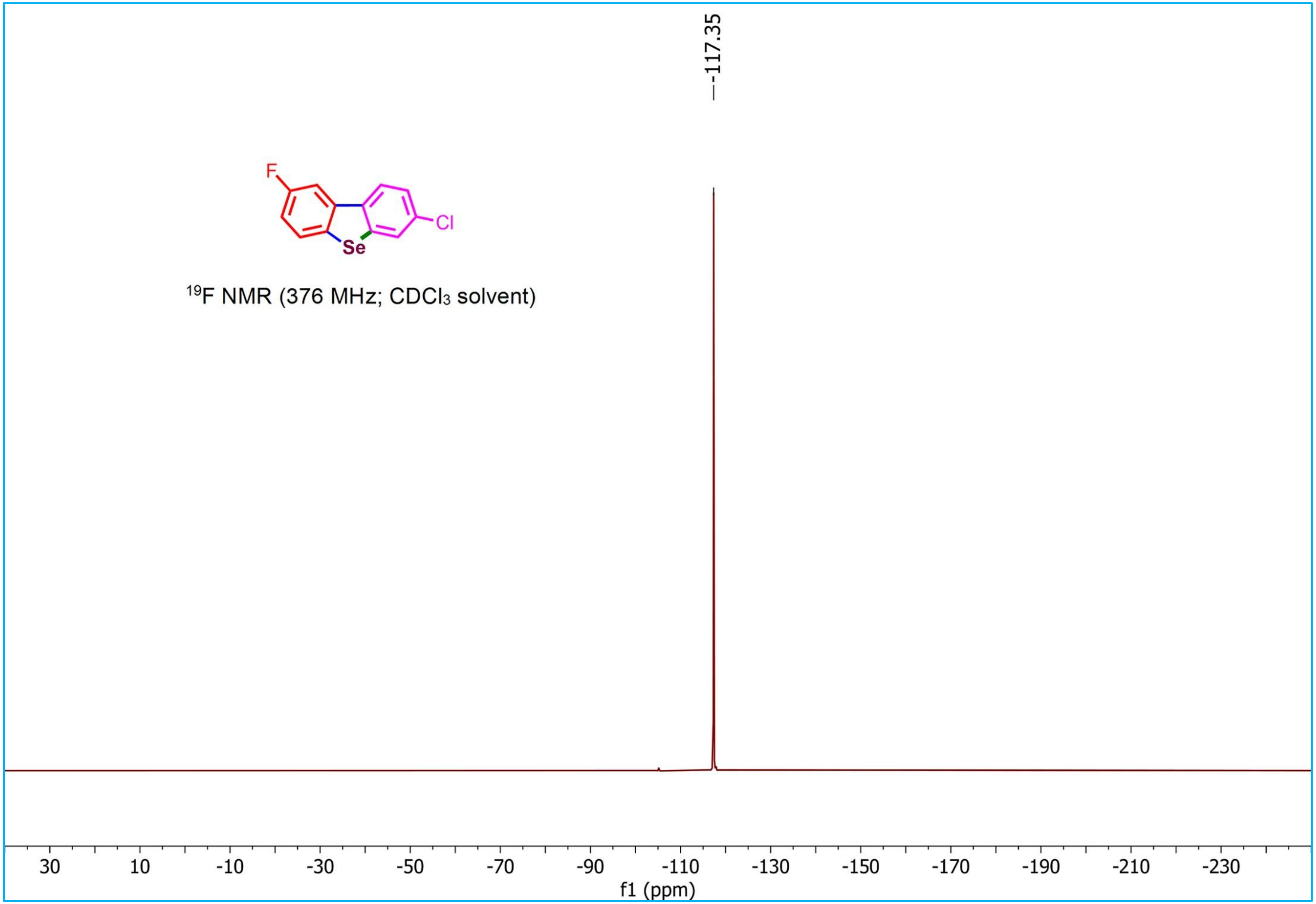

Figure S39. ^{19}F NMR spectrum of 7-chloro-2-fluorodibenzo[b,d]selenophene (**2dd**)

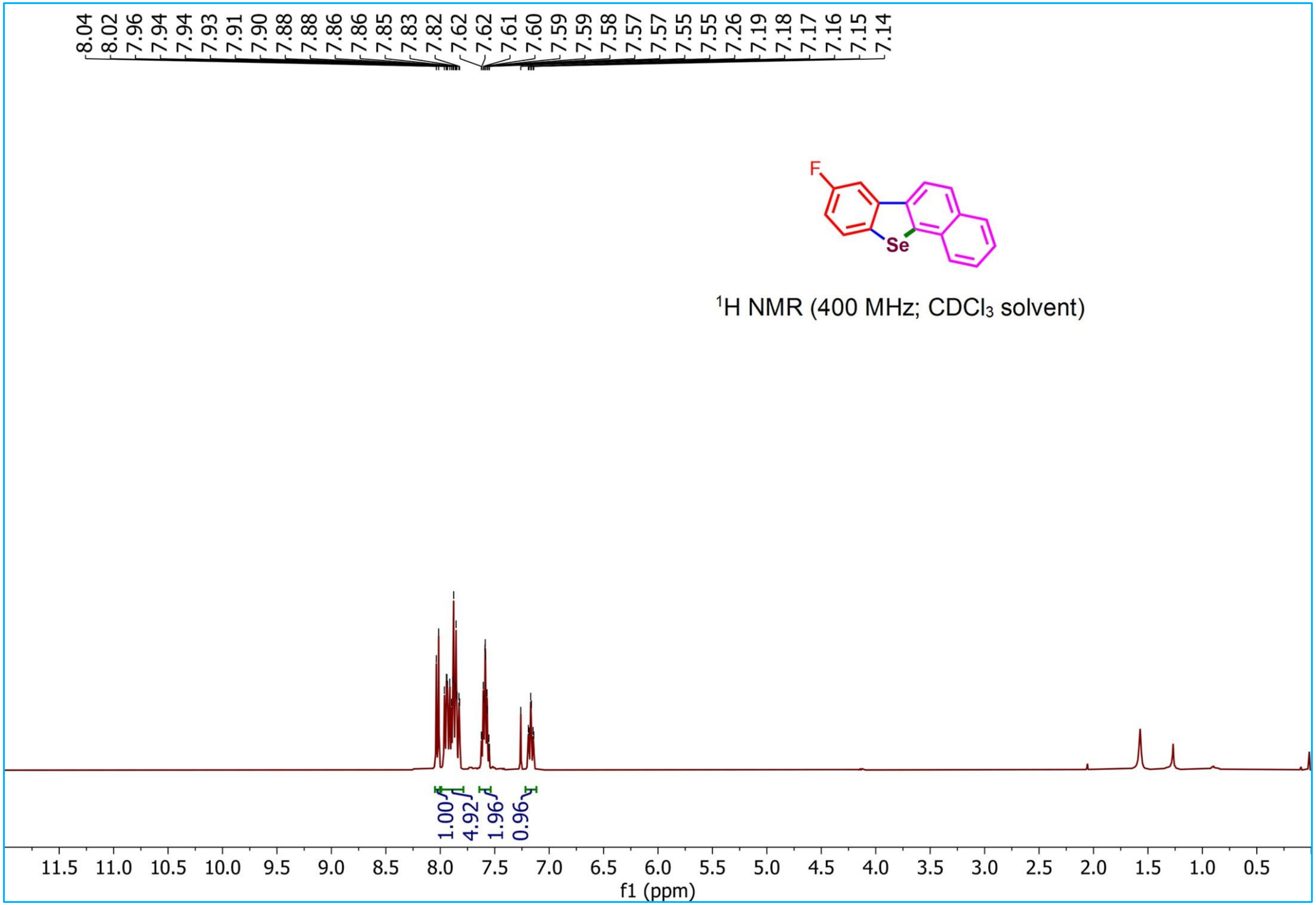

Figure S40. ^{1}H NMR spectrum of 8-fluorobenzo[b]naphtho[2,1-d]selenophene (**2dg**)

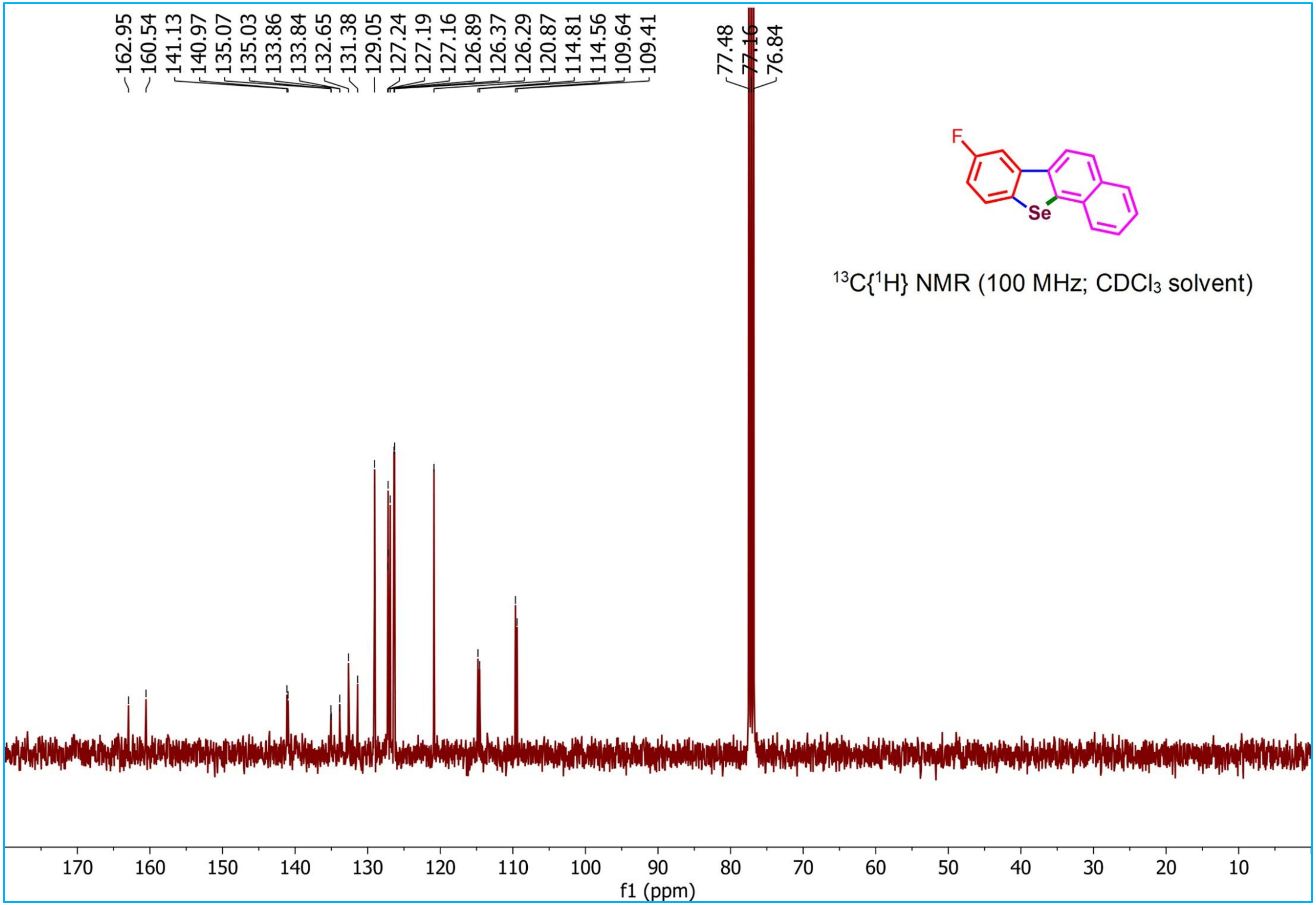

Figure S41. $^{13}C\{^{1}H\}$ NMR spectrum of 8-fluorobenzo[b]naphtho[2,1-d]selenophene (**2dg**)

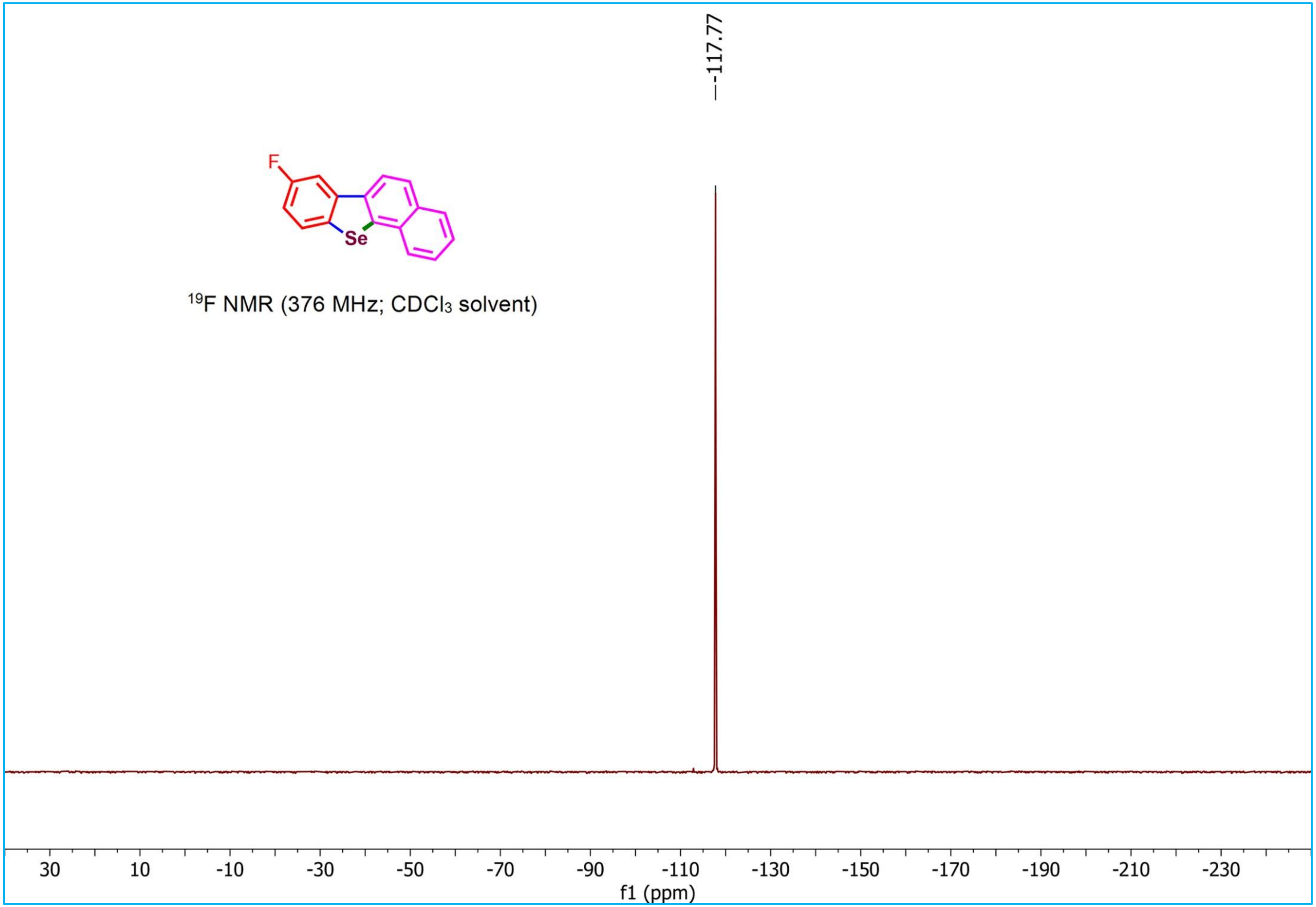

Figure S42. ^{19}F NMR spectrum of 8-fluorobenzo[b]naphtho[2,1-d]selenophene (**2dg**)

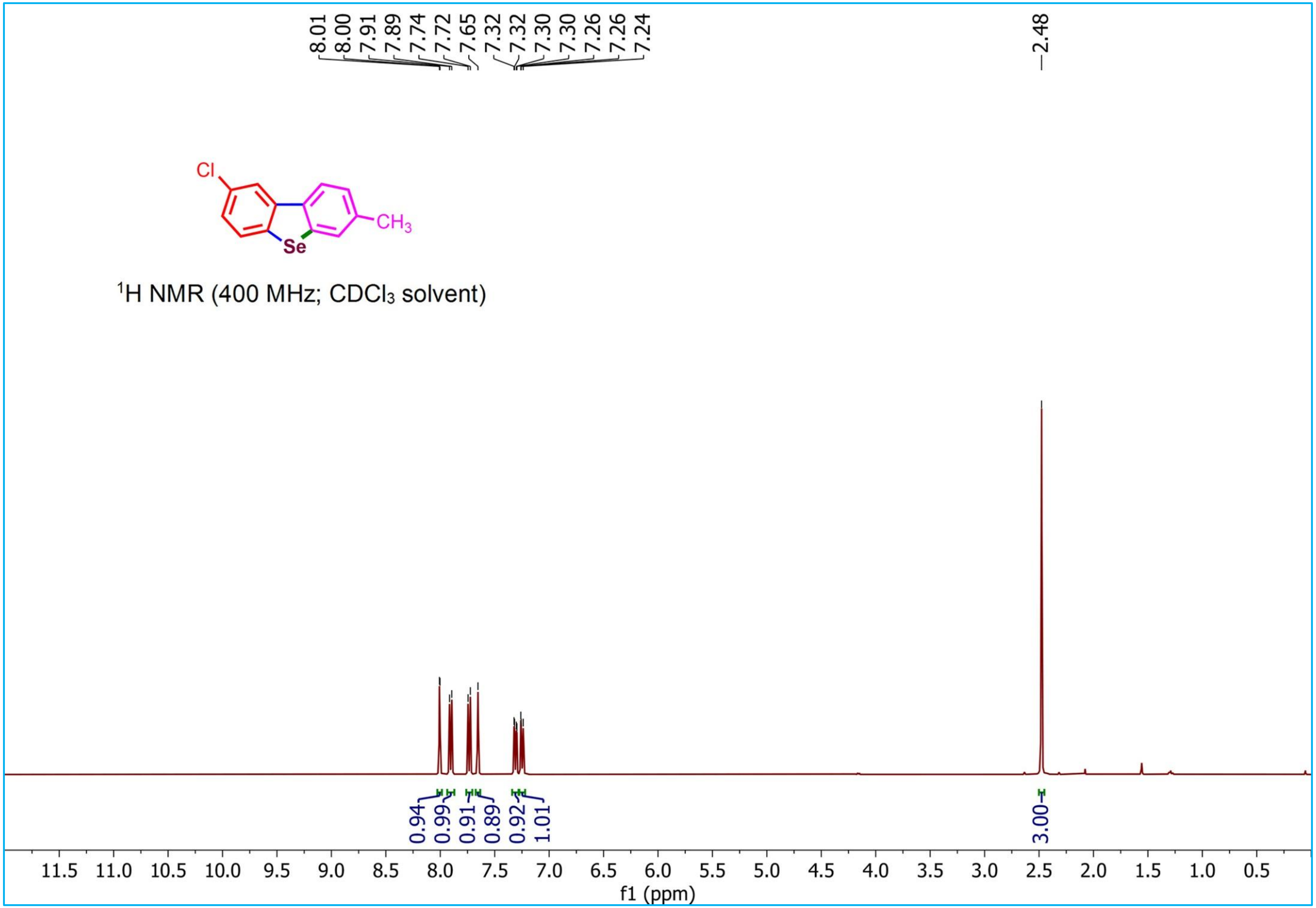

Figure S43. ^{1}H NMR spectrum of 2-chloro-7-methyldibenzo[b,d]selenophene (**2eb**)

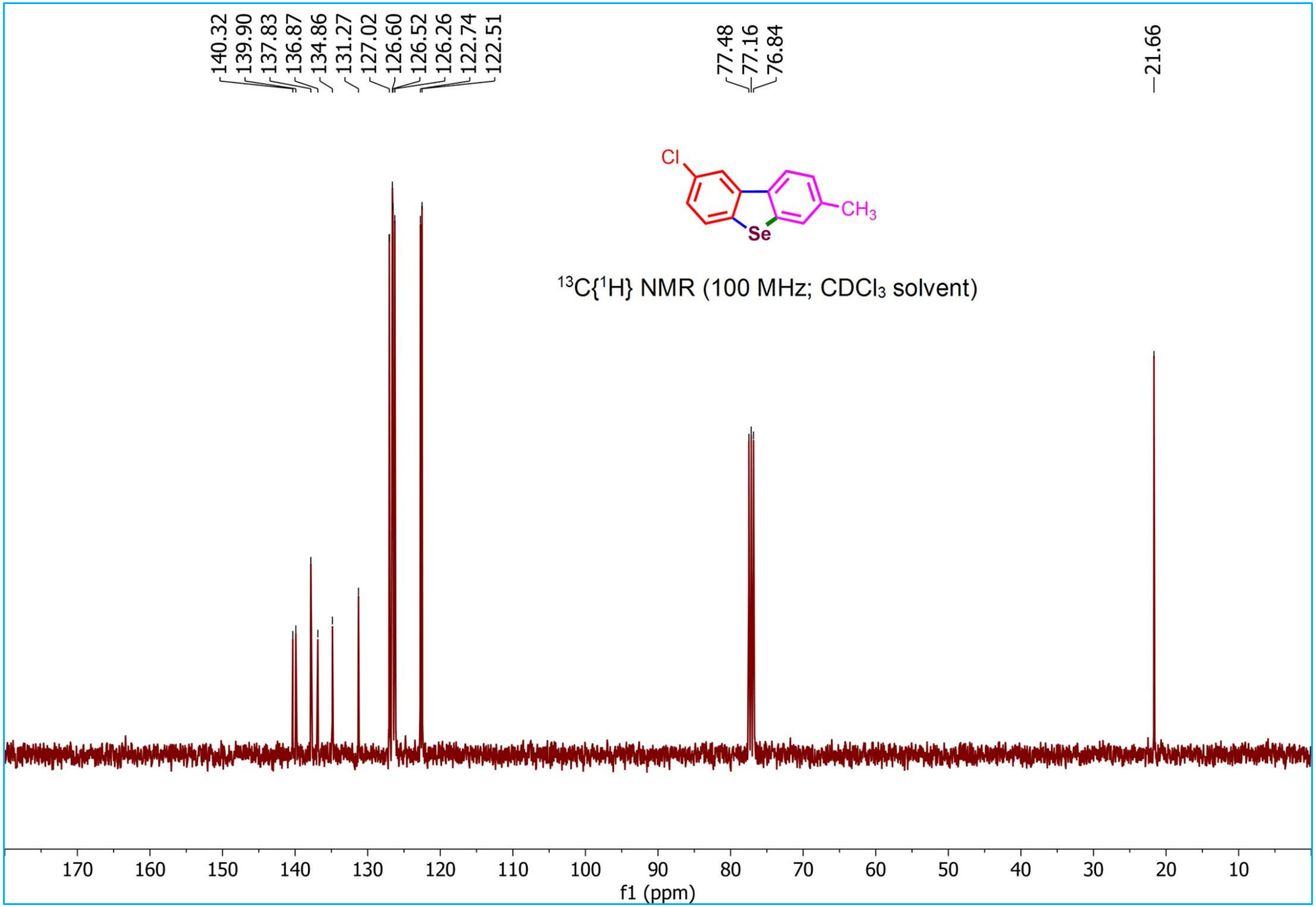

Figure S44. $^{13}C\{^{1}H\}$ NMR spectrum of 2-chloro-7-methyldibenzo[b,d]selenophene (**2eb**)

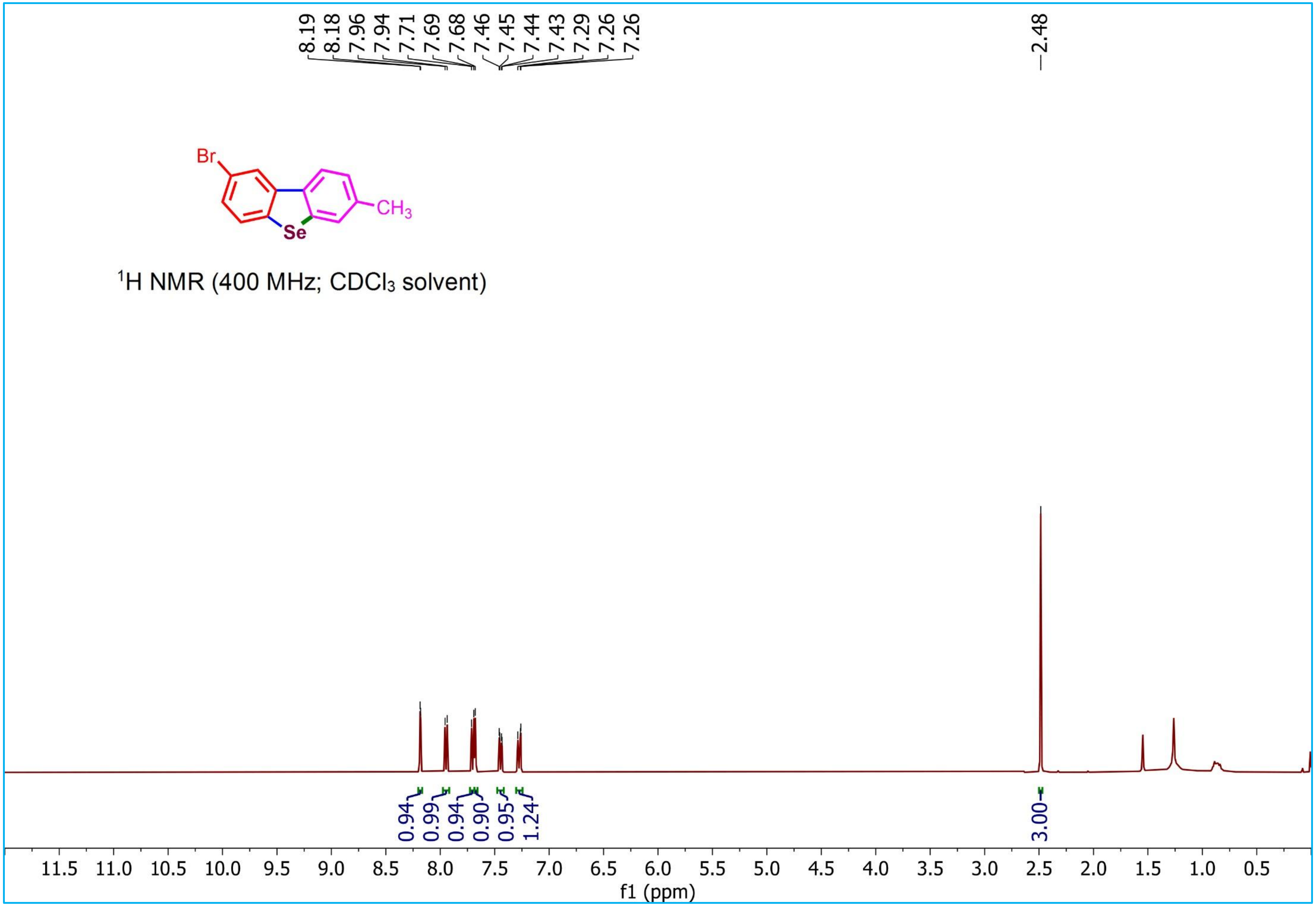

Figure S45. ^{1}H NMR spectrum of 2-bromo-7-methyldibenzo[b,d]selenophene (**2fb**)

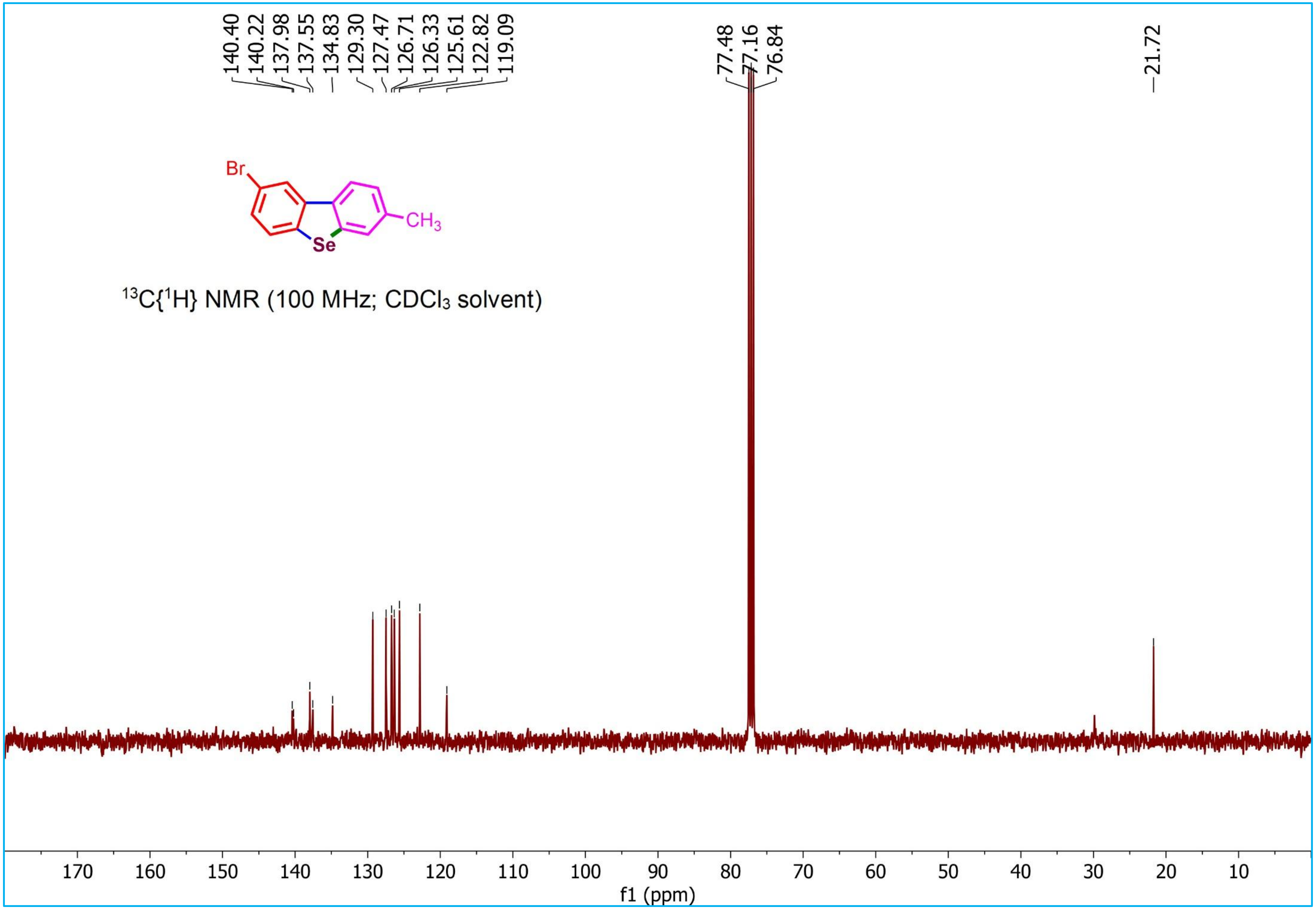

Figure S46. $^{13}C\{^1H\}$ NMR spectrum of 2-bromo-7-methyldibenzo[b,d]selenophene (**2fb**)

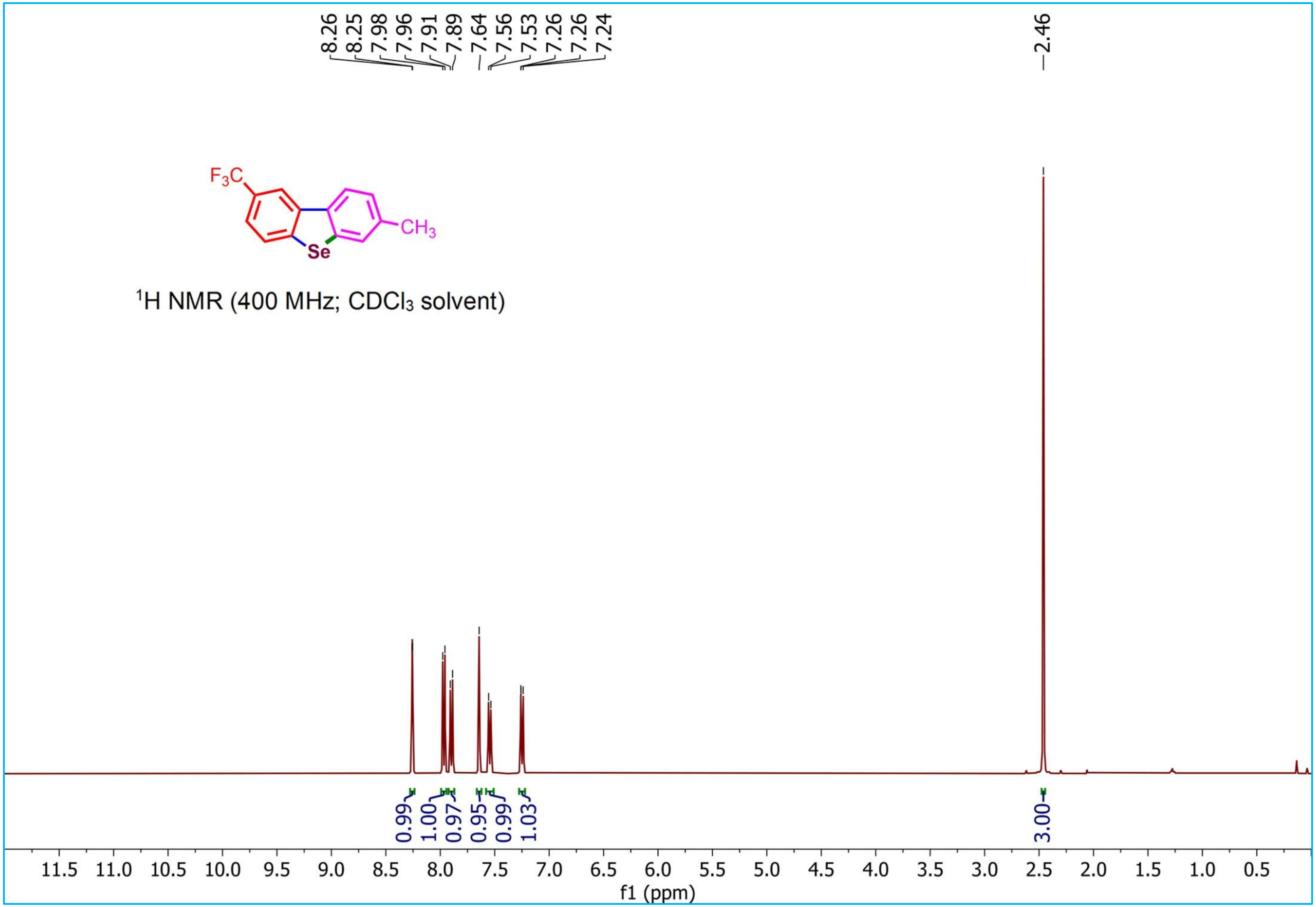

Figure S47. ^{1}H NMR spectrum of 7-methyl-2-(trifluoromethyl)dibenzo[b,d]selenophene (**2gb**)

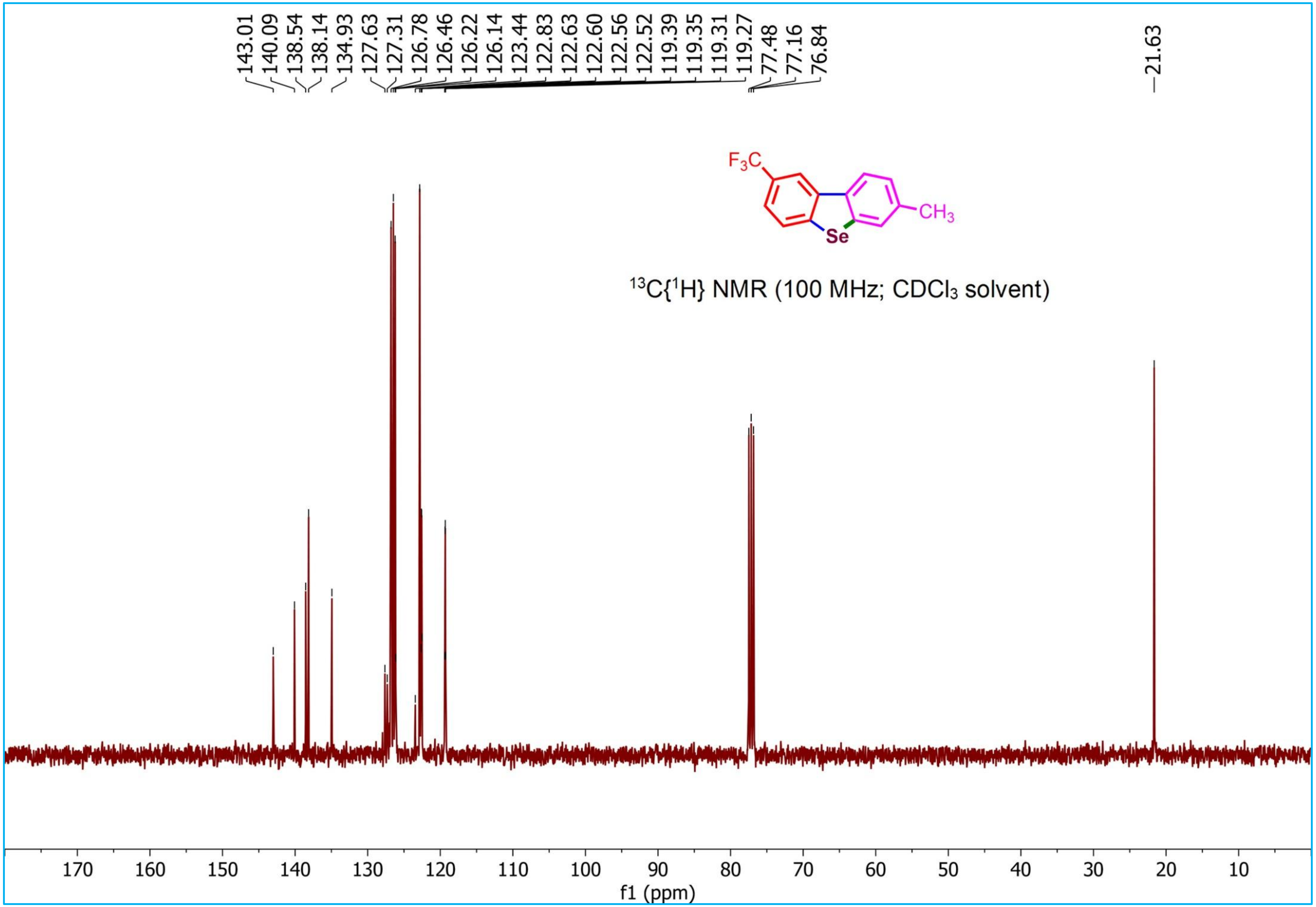

Figure S48. $^{13}C\{^{1}H\}$ NMR spectrum of 7-methyl-2-(trifluoromethyl)dibenzo[b,d]selenophene (**2gb**)

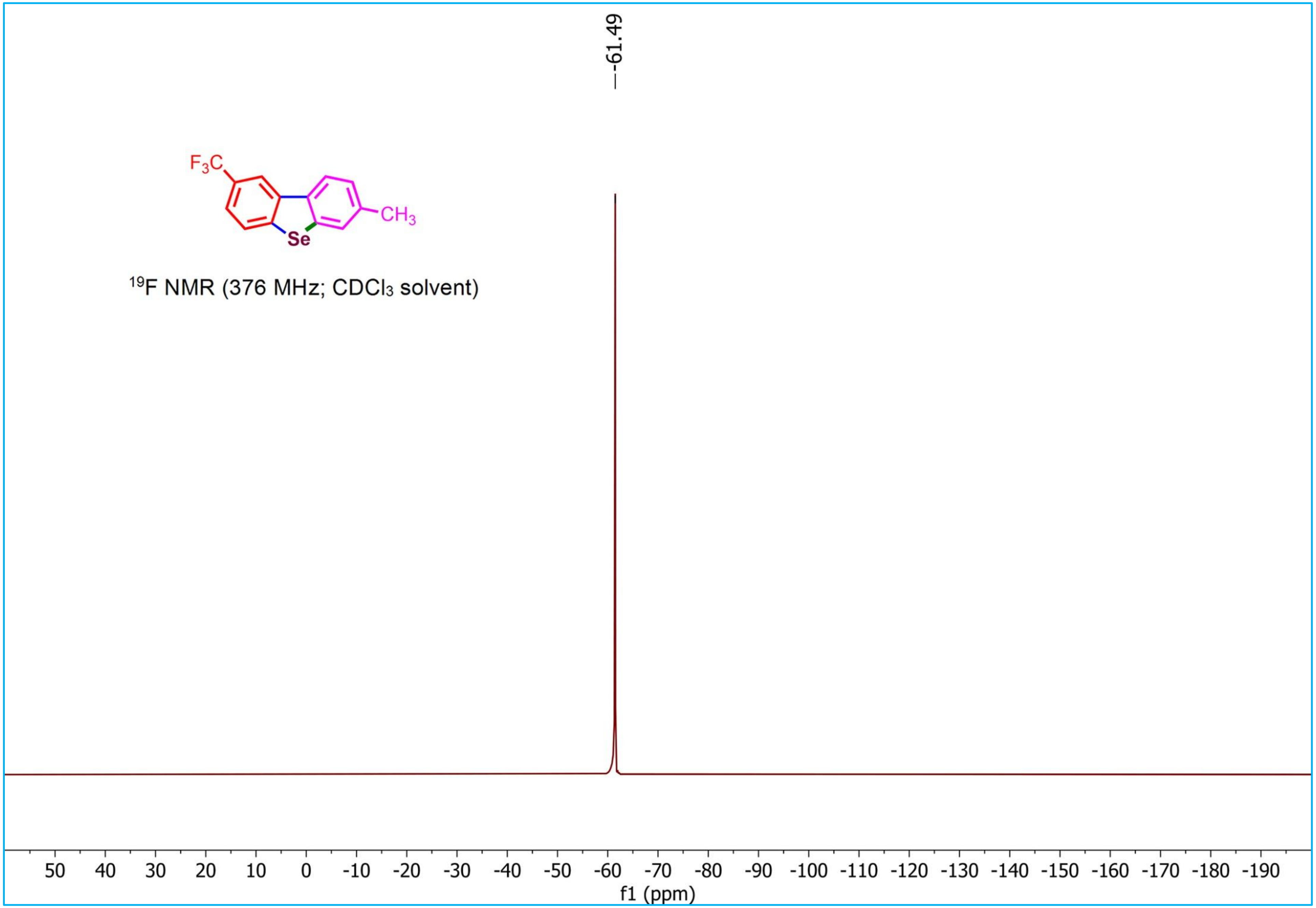

Figure S49. ^{19}F NMR spectrum of 7-methyl-2-(trifluoromethyl)dibenzo[b,d]selenophene (**2gb**)

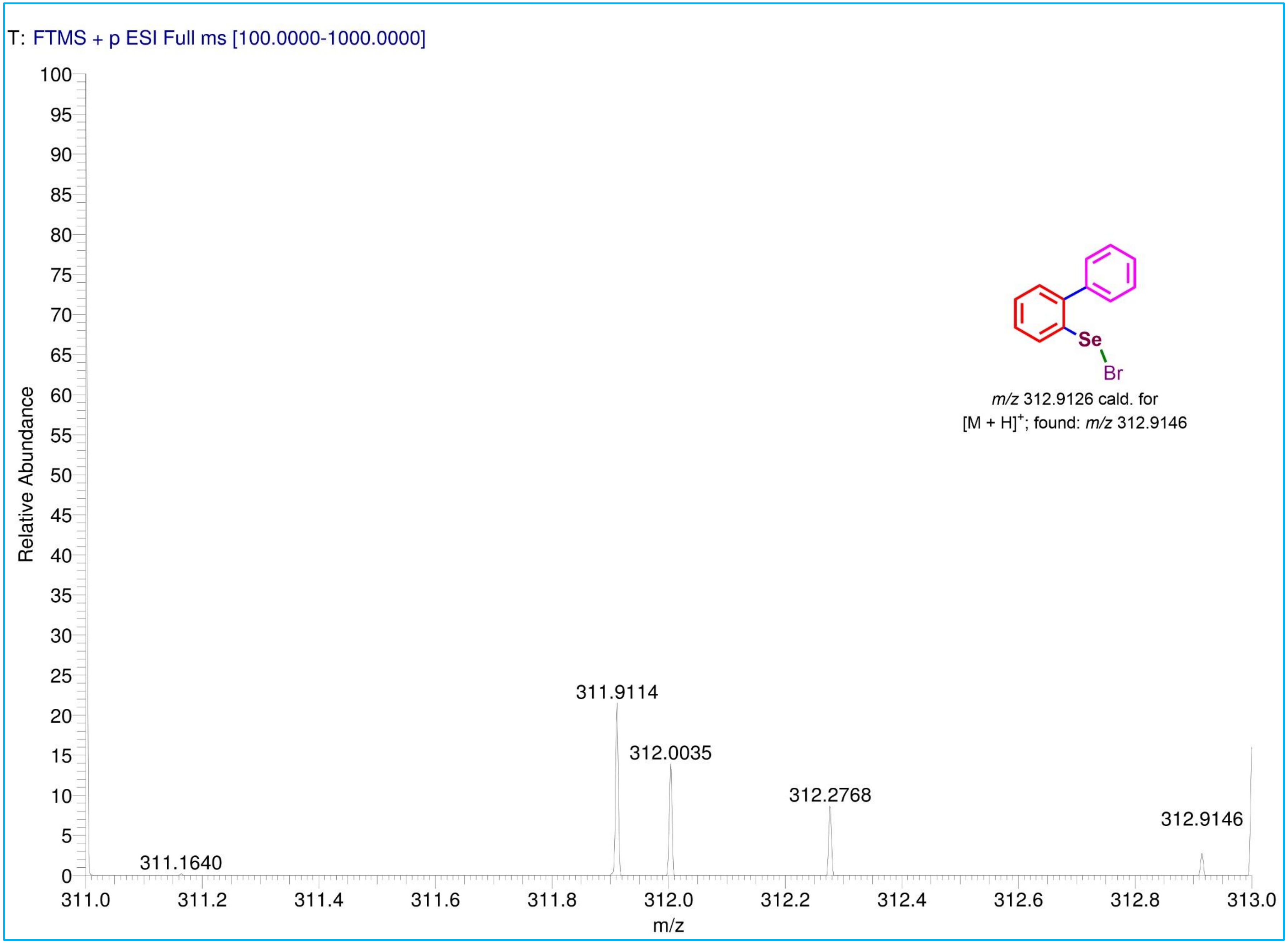

Figure S50. HRMS spectrum of intermediate **3**

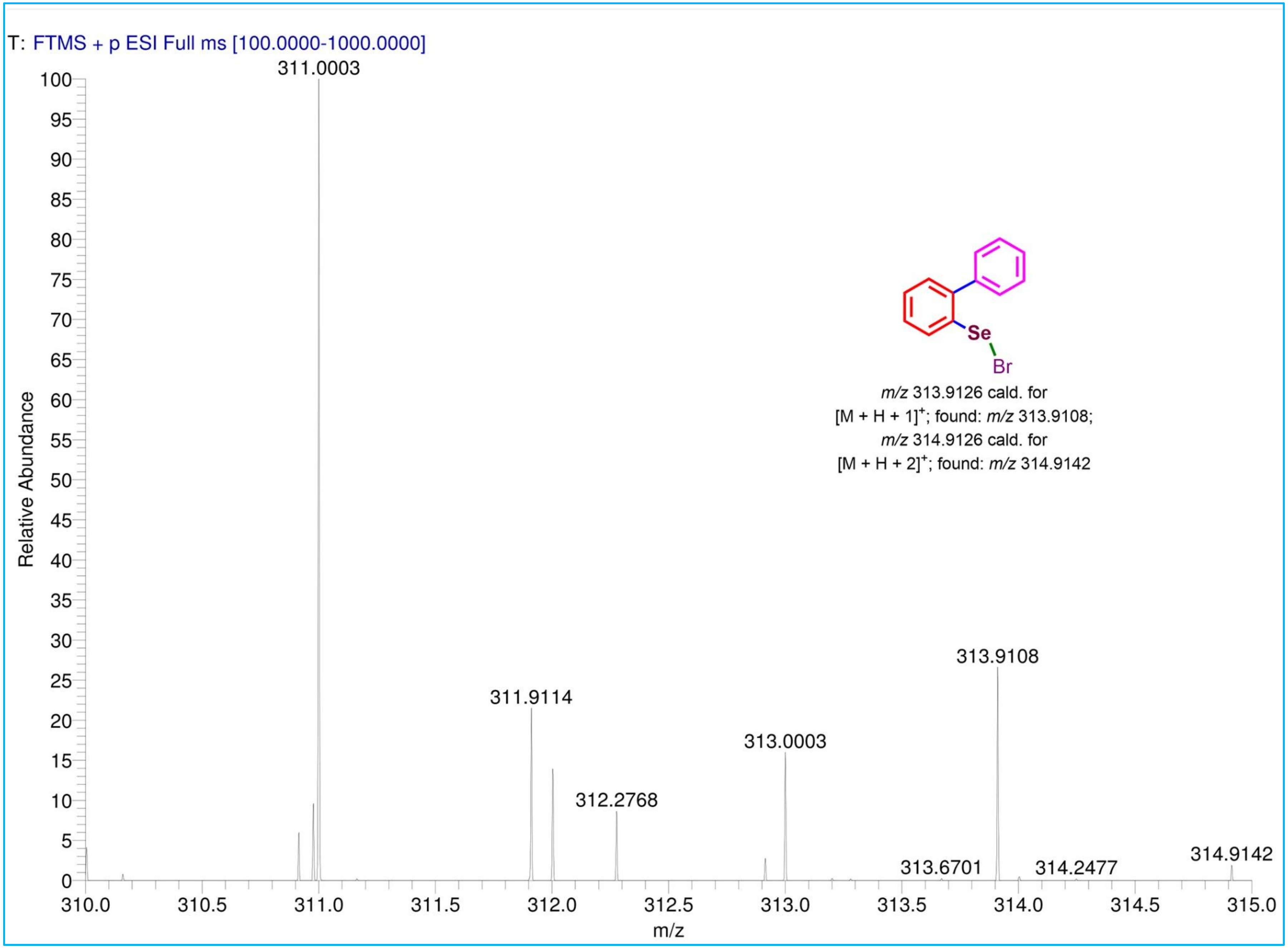

Figure S51. HRMS spectrum of intermediate **3**

9. References:

1. O.V. Dolomanov, L. J. Bourhis, R. J Gildea, J. A. K. Howard, H. Puschmann, *J. Appl. Cryst.*, 2009, **42**, 339-341.
2. G. M. Sheldrick, *Acta Cryst.*, 2008, **A64**, 112-122.
3. G. M. Sheldrick, *Acta Cryst.*, 2015, **C71**, 3-8.
